# m^6^A modification suppresses innate anti-tumour immunity in colorectal cancer by limiting alu-derived dsRNA accumulation

**DOI:** 10.1038/s41467-026-73211-z

**Published:** 2026-05-14

**Authors:** Yucheng Wang, Alice A. Daddi, Amir Hosseini, Kazuki Kato, Ehsan Khalili, Håvard T. Lindholm, Mengjie Li, Yadong Wang, David C. Michael, Catherine A. O’Brien, Daniel D. De Carvalho, Jan Rehwinkel, Parinaz Mehdipour

**Affiliations:** 1 Ludwig Institute for Cancer Research, Nuffield Department of Medicine, University of Oxford, Old Road Campus Research Building, Roosevelt Drive, Headington, Oxford, UK; 2 Mechanistic Immunology Research Unit, Institute of Integrated Research, Institute of Science Tokyo, Bunkyo-ku, Tokyo Japan; 3 Department of Pathology, Oslo University Hospital-Rikshospitalet, Oslo, Norway; 4 Princess Margaret Cancer Centre, University Health Network, Toronto, Ontario Canada; 5 Department of Medical Biophysics, University of Toronto, Toronto, Ontario Canada; 6 Department of Laboratory Medicine and Pathobiology, University of Toronto, Toronto, Ontario Canada; 7 Department of Physiology, University of Toronto, Toronto, Ontario Canada; 8 Department of Surgery, Toronto General Hospital, Toronto, Ontario Canada; 9 Medical Research Council Translational Immune Discovery Unit, Medical Research Council Weatherall Institute of Molecular Medicine, Radcliffe Department of Medicine, University of Oxford, Oxford, UK

**Keywords:** Immune evasion, RNA modification, Cancer therapy

## Abstract

How cancer cells evade immune detection despite expressing immunostimulatory retroelement (RE) transcripts remains unclear. In cancer, endogenous REs that escape epigenetic silencing are transcribed and can form double-stranded RNA (dsRNA), which activates innate immune responses through viral mimicry. However, RNA-level mechanisms can limit this effect. Here we show that the m^6^A RNA methyltransferase METTL3 acts as a key regulator of this suppression in colorectal cancer (CRC). Targeting METTL3 increases the accumulation of dsRNAs derived from both pre-existing and newly transcribed REs, amplifying immunostimulatory signalling and activating cell-intrinsic anti-tumour immunity. CRCs display variable sensitivity to METTL3 inhibition: tumours with high basal dsRNA and RNA methylation respond to METTL3 blockade alone, whereas those with low RNA methylation require combination therapy. Co-treatment with DNA methyltransferase inhibitors (DNMTis) restores immune activation in resistant tumours. Together, our findings identify METTL3 as an RNA-level immune checkpoint and suggest combined METTL3 and DNMT inhibition as a therapeutic strategy in CRC.

## Abstract (web summary)

Retroelement transcripts-derived double-stranded RNAs (dsRNAs) can trigger innate immune responses in cancer through a process known as viral mimicry. Here the authors show that METTL3, the writer of m6A modification, regulates the abundance of steady-state dsRNAs and it could be targeted together with DNA methyltransferases to enhance viral mimicry in colorectal cancer therapy.

## Introduction

Epigenetic modifications regulate gene expression, and these reversible modifications are crucial for mediating biological processes under normal physiological conditions. However, these modifications can be altered during tumourigenesis. While targeting epigenetic regulators in haematological malignancies shows promise, it is currently inefficient in treating solid tumours^1^. Traditionally, studies on the mechanisms of epigenetic therapies in cancer cells have relied on identifying transcriptionally altered genes, such as tumour suppressors and oncogenes^2–5^. However, the recent discovery of the viral mimicry phenomenon as a novel therapeutic vulnerability has shifted the paradigm of cancer therapies^6,7^.

Epigenetic therapies, such as DNMTis and histone methyltransferase targeting approaches, including SETDB1 inhibition, have been shown to reactivate not only epigenetically silenced genes but also REs within the human genome^6–8^. Beyond epigenetic regulation, RE activation is also controlled by non-epigenetic factors such as splicing^9^. The activation of REs generates RE-derived RNAs that form double-stranded (ds) structures, mimicking viral RNA. These endogenous dsRNAs are immunostimulatory, activating innate immune signalling and promoting anti-tumour immune responses. Consequently, cancer cells lose their fitness, and this cellular state is known as viral mimicry^6,7,10,11^.

Transposable elements are repetitive DNA sequences that play a significant role in the evolution and regulation of the human genome. Most transposable elements (TEs) are REs, such as Short interspersed nuclear elements (SINEs), Long interspersed nuclear elements (LINEs), and Long terminal repeats (LTRs), which move within the genome using a copy-and-paste mechanism that involves an RNA intermediate, similar to retroviruses. Under normal physiological conditions, various mechanisms, such as epigenetic modifications, typically keep REs silenced due to their potential genetic risks^12^. We and others have shown there is an additional layer of regulation for the activity of REs at the RNA level, mediated by RNA editase, ADAR1^13,14^. If REs evade histone and DNA silencing mechanisms and become transcribed, the resulting RE-derived RNAs, particularly those from the SINE family, which are immunostimulatory, are edited and destabilised by ADAR1. Our findings indicate that targeting ADAR1 could represent a promising strategy for enhancing cancer therapy^13–16^. However, despite its potential as a novel therapeutic target, no commercially available ADAR1 inhibitors currently exist^17^.

Another RNA modifier, which shows promise as a target for treating haematological malignancies, is RNA methyltransferase 3 (METTL3)^18,19^, the key catalytic component of the N6-methyladenosine (m^6^A) RNA modification complex. m^6^A is the most abundant mRNA modification, typically enriched near the stop codons and plays a crucial role in regulating mRNA stability, splicing, and translation^20–23^. Recent studies have demonstrated that m^6^A writer METTL3 plays a critical role in modulating immune homoeostasis. Hypomethylation of m^6^A RNA has been associated with the activation of interferon (IFN) responses through the stabilisation of IFNβ mRNA, conformational changes in the RNA sensor RIG-I, and the reactivation of IAP endogenous retroviral elements^24–26^. Evidence further suggests that during haematopoiesis, m^6^A RNA modifications on long endogenous transcripts regulate immune responses^27^. Recently, Guirguis and colleagues demonstrated that a METTL3 inhibitor, STM3006, activates a cell-intrinsic interferon response and that METTL3 inhibition synergises with anti-PDL1 therapy in triple-negative breast cancer cells in mice^28^. Furthermore, METTL3 inhibitors are currently being evaluated in clinical trials for the treatment of solid tumours, highlighting the therapeutic potential of targeting RNA methylation^29^. However, while previous studies have reported that CRC cells respond to METTL3 targeting^30,31^, the extent to which METTL3 dependency varies across CRCs and the mechanisms driving this differential vulnerability remain unclear.

CRC is the third most commonly diagnosed cancer worldwide and the second leading cause of cancer-related mortality^32^. Despite therapeutic advances, relapse rates remain high, and epigenetic therapies have shown limited benefit in CRC and other solid tumours. Furthermore, the rising incidence of CRC in younger adults highlights an urgent need to identify new therapeutic vulnerabilities^33^. Our study, therefore, centres on CRC to determine whether METTL3 inhibition could represent a viable therapeutic strategy in this clinically significant and therapeutically challenging disease.

In this study, we demonstrate that METTL3 suppresses viral mimicry responses and uncover the molecular mechanisms underlying CRC susceptibility to METTL3 targeting. Our findings show that TE expression does not necessarily correlate with interferon-stimulated gene (ISG) activation. Instead, METTL3 inhibition increases the accumulation of RE-derived immunostimulatory dsRNA, indicating that METTL3 regulates viral mimicry at the RNA level. Through systematic functional assays, we demonstrate that targeting METTL3 activates intrinsic innate immune responses in a subset of CRCs. Moreover, we find that METTL3 inhibition synergises with DNMTis to enhance viral mimicry, effectively eliminating cancer cells that are resistant to METTL3 targeting alone.

## Results

### Colorectal cancer cells show different sensitivity to *METTL3* targeting

**Fig. 1:**
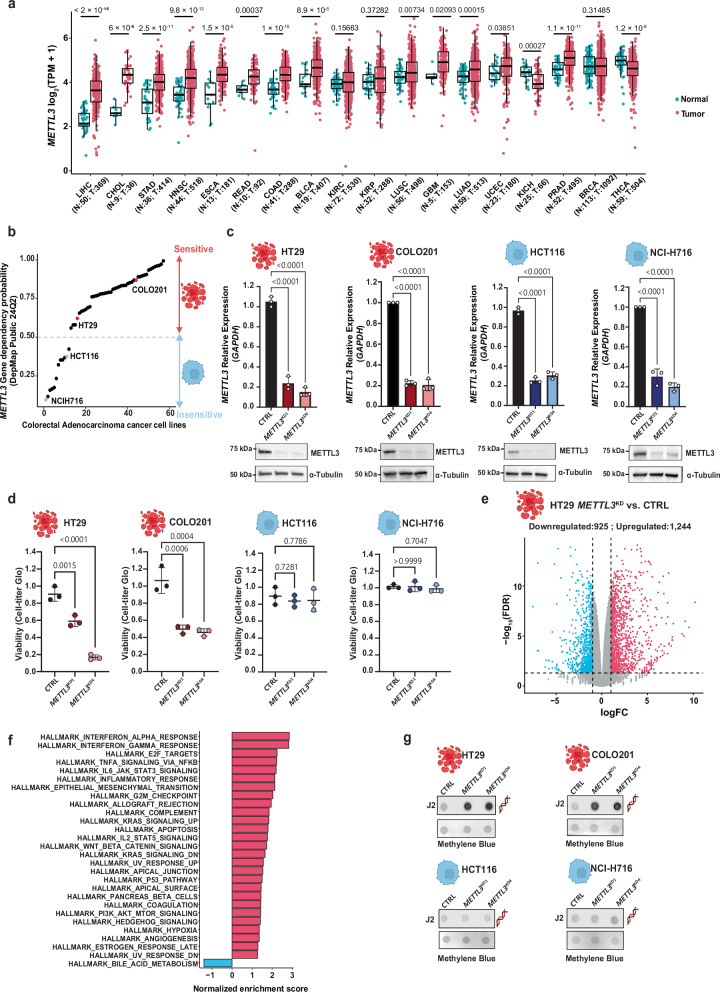
Colorectal cancer cells exhibit varying sensitivity to *METTL3* targeting. **a** *METTL3* expression in tumour (T) and normal (N) samples across 18 cancer types from TCGA. Significance was assessed using a two-sided Wilcoxon rank-sum test; sample numbers are indicated on the x-axis. Boxes represent the median (central line) and the interquartile range (IQR; 25th to 75th percentiles). Whiskers extend to 1.5 × IQR from the quartiles; observations beyond this range are not shown. **b** Scatter plot showing *METTL3* dependency in colorectal adenocarcinoma (COAD) cell lines based on DepMap Public 24Q2^36^. Cell lines with a sensitivity probability score <0.5 were classified as *METTL3*-insensitive, whereas those with a score > 0.5 were classified as *METTL3*-sensitive. Red icons denote sensitive lines; blue icons denote insensitive lines. Cell icons in this and other figures were created in BioRender. Mehdipour, P. (2026) https://BioRender.com/d7qjvyg. **c** qPCR (top) and immunoblot (bottom) analyses of *METTL3* knockdown (^KD^) efficiency using two different short hairpin RNAs (shRNAs; ^KD1, KD4^) in CRC cell lines. qPCR data are presented as mean ± SD (*n* = 3 independent experiments). Statistical significance was determined by Dunnett-corrected one-way ANOVA. A representative immunoblot from three independent experiments showing METTL3 protein levels is presented, with α-tubulin serving as a loading control. **d** Viability of *METTL3* wild-type (CTRL) and *METTL3*^KD^ CRC cells, as measured by the CellTiter-Glo luminescence assay. Data are presented as mean ± SD (*n* = 3 independent replicates). Statistical significance was determined by Dunnett-corrected one-way ANOVA. **e** Volcano plot showing changes in gene expression comparing *METTL3*^KD^ with CTRL cells. (Red, upregulated; grey, no significant change; blue, downregulated.) Statistical significance was defined as FDR < 0.05 and |logFC | ≥ 1. (n = 2 independent experiments). **f** Gene set enrichment analysis (GSEA) identified enriched MSigDB Human Hallmark gene sets in HT29 *METTL3*^KD^ compared with CTRL. Red colour indicates gene sets enriched in the *METTL3*^KD^ group, whereas blue colour indicates those enriched in the CTRL group. **g** Double-stranded RNA (dsRNA) dot blot analysis using total RNA from CRC cells. Normalised amounts of total RNA were spotted onto Hybond N^+^ membranes, immunoblotted with the J2 antibody, and visualised by methylene blue staining. Representative dot blots from three independent experiments. Source data are provided as a Source Data file.

To evaluate the *METTL3* gene expression profile, we first analysed transcriptome sequencing data from The Cancer Genome Atlas (TCGA) for normal and cancerous tissues across various cancer types (Fig. 1a). This analysis revealed that *METTL3* expression is elevated in cancerous tissues compared to corresponding normal tissues in the majority of cancers, including colorectal adenocarcinoma (COAD), consistent with previous reports^30,34,35^. Next, to broadly assess the dependency of CRCs on METTL3, we analysed publicly available genome-scale loss-of-function screening datasets (DepMap)^36^. This analysis identified *METTL3* as an essential gene in a subset of CRCs (Fig. 1b).

To further investigate the role of METTL3 in CRC cells, we established *METTL3*-knockdown (^KD^) HT29 and HCT116 cell lines using four distinct shRNAs (Supplementary Fig. 1a) and selected KD1 and KD4 for subsequent analyses. We also generated *METTL3*^KD^ COLO201 and NCI-H716 CRC cell lines, as well as two patient-derived xenograft (PDX) CRC models, POP92 and CSC73 (Fig. 1c and Supplementary Fig. 1b). Viability assays demonstrated that *METTL3*^KD^ significantly reduced the viability of HT29, COLO201, and POP92 cells, and impaired the colony-forming ability of HT29 cells. In contrast, *METTL3*^KD^ had no significant effect on the viability of HCT116, NCI-H716, or CSC73 cells, nor on the colony-forming ability of HCT116 cells (Fig. 1d and Supplementary Fig. 1c, d). Although CRC cells have been shown to respond to *METTL3* targeting^30,31^, our findings uncover an additional layer of complexity: CRC models vary substantially in their vulnerability to METTL3 inhibition.

### METTL3 depletion induces dsRNA-driven viral mimicry in a subset of CRC models

To investigate the effects of *METTL3* targeting in CRC, we performed RNA sequencing (RNA-seq) on control (CTRL) and *METTL3*^KD^ HT29, COLO201, HCT116, NCI-H716 and CSC73 cells. *METTL3*^KD^ resulted in differential expression of a substantial number of genes in HT29, COLO201, NCI-H716, and CSC73 cells (Fig. 1e and Supplementary Fig. 1e). In contrast, the transcriptomic effect in HCT116 cells was minimal, with only 45 genes downregulated and 34 upregulated (Supplementary Fig. 1e).

Next, we selected the top 200 upregulated differentially expressed genes in *METTL3*^KD^ HT29 cells relative to CTRL as a representative METTL3-sensitive CRC model and performed pathway and network enrichment analyses. Notably, type I interferon response and defence response to viruses emerged as the most enriched Gene Ontology (GO) terms and network modules in HT29 cells (Supplementary Figs. 1f, 2a). Similarly, Gene Set Enrichment Analysis (GSEA) revealed strong enrichment of the Interferon (IFN) alpha hallmark gene set in *METTL3*^KD^ HT29 cells compared with CTRL (Fig. 1f and Supplementary Fig. 2b).

Further analysis demonstrated that *METTL3*^*KD*^ in HT29, COLO201, and POP92 cells that are sensitive to *METTL3* depletion induced robust expression of interferon-stimulated genes (ISGs), which are typically driven by type I/III IFNs, whereas ISG levels remained largely unchanged in the *METTL3*-insensitive HCT116, NCI-H716, and CSC73 cells (Supplementary Fig. 2c, d). To assess whether basal ISG expression could predict CRC sensitivity to METTL3 loss, we quantified the average scaled expression of 38 ISGs (Supplementary Data 1)^37^ and compared these values with *METTL3* dependency probabilities from the DepMap dataset^36^. We found no significant correlation between basal ISG scores and METTL3 dependency across CRC cell lines (Supplementary Fig. 2e). Consistent with these observations, RNA-seq analysis showed that HT29 cells, despite being sensitive to *METTL3* targeting, display lower basal ISG expression than the insensitive HCT116 and CSC73 cells. By contrast, POP92 and COLO201 cells, which are also *METTL3*-sensitive, exhibit higher basal ISG levels (Supplementary Fig. 2f). Extending this analysis to other cancer types revealed that ISG scores correlated positively with *METTL3* dependency in ovarian cancer stem cells (OCSC) and small cell lung cancer (SCLC), but negatively in lung adenocarcinoma (LUAD) (Supplementary Fig. 2g), indicating a context-dependent relationship. Together, these analyses suggest that basal ISG expression is not a reliable biomarker for predicting CRC dependency on *METTL3*.

To determine whether ISG induction in *METTL3*^KD^ sensitive cells is mediated by the accumulation of endogenous dsRNAs, we performed RNA dot blot and immunofluorescence staining analysis using the J2 monoclonal antibody against dsRNA. These analysis indicated a significant increase in dsRNA levels in HT29, COLO201 and POP92 *METTL3*^KD^ cells compared with CTRL cells, whereas HCT116, NCI-H716 and CSC73 *METTL3*^*KD*^ cells showed no substantial changes in dsRNA levels (Fig. 1g and Supplementary Fig. 3a–e). Taken together, these results demonstrate that targeting *METTL3* reduces the viability of a subset of CRC cells by promoting the accumulation of endogenous dsRNA levels and activation of ISGs.

**Fig. 2:**
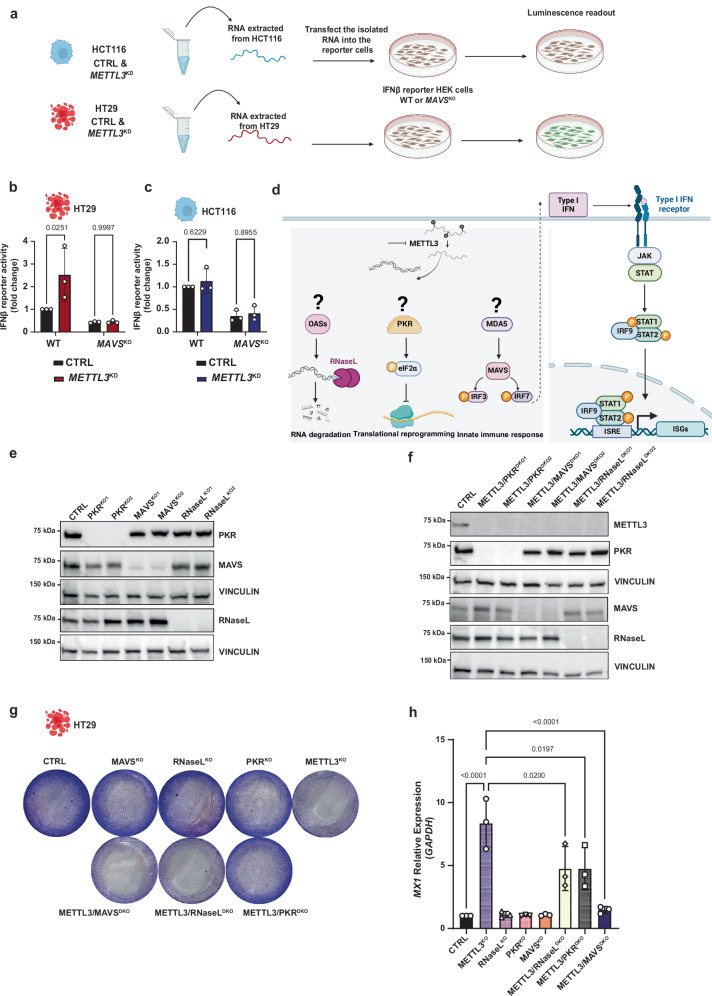
Loss of METTL3 in a subset of CRCs triggers IFN responses and promotes cancer cell death. **a** Schematic representation of the IFNβ reporter assay. P125-HEK WT and *MAVS*^KO^ reporter cells were transfected with RNA isolated from CTRL and *METTL3*^KD^ HCT116 or HT29 cells, and luciferase activity was measured using a luminescence assay. Created in BioRender. Mehdipour, P. (2026) https://BioRender.com/t07l048. **b c** Bar plot showing normalised IFNβ reporter activity (fold change) in p125-HEK WT and p125-HEK *MAVS*^KO^ reporter cells following stimulation with RNA isolated from HT29 (**b**) and HCT116 (**c**) CTRL and *METTL*3^KD^ cells. Data are presented as mean ± SD (*n* = 3 independent experiments). Statistical significance was determined by Sidak-corrected two-way ANOVA. **d** Schematic representation of pattern recognition receptors (PRRs) sensing dsRNA: OAS (2′–5′-Oligoadenylate Synthetase) activates RNase L to mediate RNA degradation; PKR induces translational reprogramming via eIF2α phosphorylation; and MDA5 promotes IFN production and ISG expression to drive antiviral responses. Created in BioRender. Mehdipour, P. (2026) https://BioRender.com/gqa7rn3. **e** Immunoblot analysis of METTL3, PKR (*EIF2AK2*), MAVS, and RNase L (*RNASEL)* in HT29 wild-type (CTRL), PKR^KO^, MAVS^KO^, and RNase L^KO^ cells. Vinculin was used as a loading control. Representative immunoblots from two independent experiments. **f** Immunoblot analysis of METTL3, PKR, MAVS, and RNase L in HT29 wild-type (CTRL) and METTL3-based double-knockout (DKO) cells. Vinculin was used as a loading control. Representative immunoblots from two independent experiments. **g** Proliferation of HT29 wild-type (CTRL), single-knockout (KO), and double-knockout (DKO) cells, as assessed by crystal violet staining. **h** Quantitative PCR (qPCR) analysis of *MX1* expression in HT29 cells. Values were normalised to *GAPDH* and are presented relative to the CTRL sample. Data are shown as mean ± SD (*n* = 3 independent experiments). Statistical significance was determined by Tukey-corrected one-way ANOVA. Source data are provided as a Source Data file.

To assess whether the induced endogenous dsRNA upon *METTL3*^KD^ could indeed activate an innate immune response, we performed an IFNβ promoter luciferase reporter assay. First, we isolated RNA from HT29 and HCT116 *METTL3*^KD^ and CTRL cells. Then HEK cells stably transduced with an IFNβ reporter were transfected with the isolated RNAs (Fig. 2a). We observed that IFNβ promoter activity was increased upon transfection of reporter cells with RNA isolated from METTL3-deficient HT29 cells (Fig. 2b), but not with RNA from HCT116 cells (Fig. 2c), suggesting that dsRNAs accumulating upon targeting *METTL3* induce IFNβ and innate immune signalling in *METTL3*-sensitive (HT29) cells but not in insensitive cells. This phenotype was rescued in Mitochondrial anti-viral signalling protein knock-out (*MAVS* ^KO^) reporter cells (Fig. 2b, c). To further investigate the Pattern Recognition Receptors (PRRs) involved in ISG induction and cell death in HT29 CRCs upon *METTL3* targeting (Fig. 2d), we generated HT29 cells with KO of key PRRs. Specifically, we generated HT29 cells with KO of *MAVS*, which mediates anti-viral responses initiated by Retinoic acid-inducible gene I (RIG-I) and Melanoma differentiation-associated gene 5 (MDA5), as well as KO of Protein Kinase R (PKR, *EIF2AK2*) and the endonuclease RNase L, either as single knockouts (Fig. 2e) or in combination with *METTL3*^KO^ (Fig. 2f). Our results showed that PKR^KO^ significantly rescued the loss of cell viability caused by METTL3 depletion in HT29 cells (Fig. 2g), while *MAVS*^KO^ rescued ISG induction following *METTL3*^KO^ (Fig. 2h). These data demonstrate that PRR-mediated sensing of dsRNA activates MAVS-dependent ISG induction and that PKR is required for *METTL3*^KD^-induced cell death in *METTL3*-sensitive HT29 cells.

### dsRNA-forming capacity determines METTL3 dependency in CRCs

**Fig. 3:**
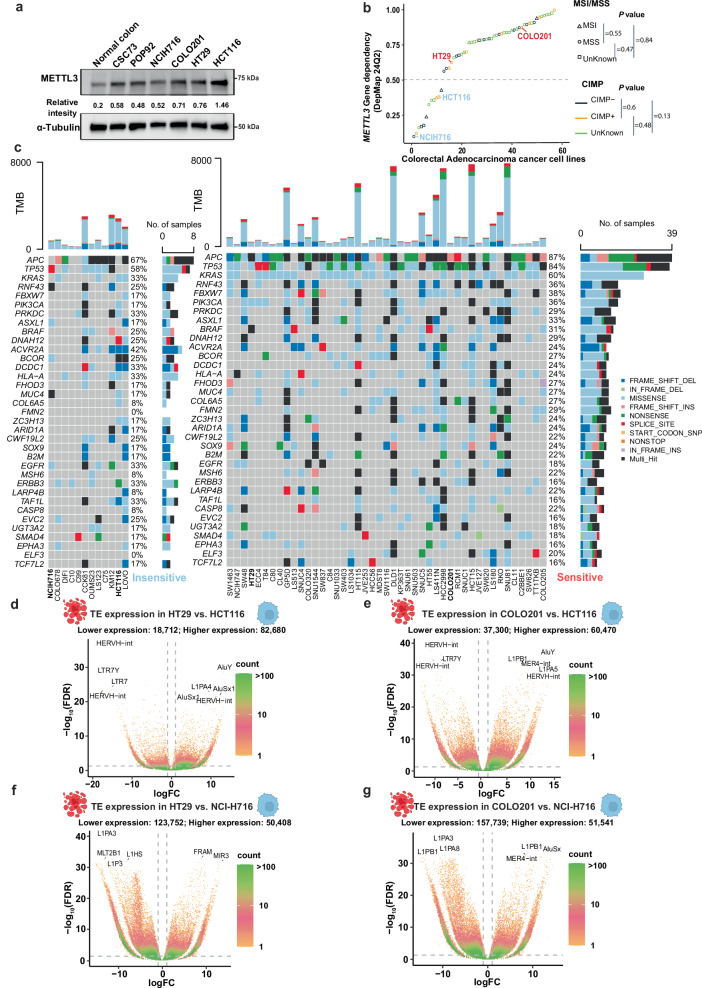
CRC dependency on *METTL3* is independent of molecular subgroups and basal TE expression levels. **a** Immunoblot analysis of METTL3 protein levels in normal colon cells, CSC73 and POP92 PDX CRC cells, and CRC cell lines (NCI-H716, COLO201, HT29, and HCT116). α-Tubulin was used as a loading control. Relative METTL3 intensity, normalised to α-tubulin in each lane, was quantified using Image Lab software. Representative immunoblots from three independent experiments. **b** Scatter plot showing the dependency of colorectal adenocarcinoma (COAD) cell lines on *METTL3*, based on data from DepMap Public 24Q2^36^. Cell lines are colour-coded according to CpG island methylator phenotype (CIMP) status^88^ and shaped by microsatellite status: microsatellite stable (MSS) or microsatellite instable (MSI). *P*-values were determined by a two-sided Wilcoxon rank-sum test. **c** Oncoplots comparing mutation profiles of 35 genes between *METTL3*-targeting sensitive (dependency score > 0.5) and *METTL3*-insensitive (dependency score < 0.5) colorectal cancer cell lines. The 35 genes were selected based on a mutation frequency ≥ 15% across colorectal cancer cell lines and their inclusion among the 131 significantly mutated genes in colorectal adenocarcinoma identified by the Broad Institute TCGA MutSigCV analysis^91^. **d**–**g** Volcano plots showing changes in transposable element (TE) expression based on total RNA sequencing comparisons between (**d**) HT29 and HCT116, (**e**) COLO201 and HCT116, (**f**) HT29 and NCI-H716, and (**g**) COLO201 and NCI-H716 cells. Colours indicate TE counts, and dotted lines denote the thresholds (FDR < 0.05 and |logFC | ≥ 1) used to define differentially expressed TEs. The data represent two independent experiments. Source data are provided as a Source Data file.

To investigate the basis of variable sensitivity to *METTL3* targeting in CRC, we assessed METTL3 protein levels in CRC models and normal colon cells. Consistent with the elevated *METTL3* transcription observed in cancerous tissues compared to normal tissues from CRC patients in the TCGA dataset (Fig. 1a), we detected higher METTL3 protein levels in CRC cells compared with normal colon cells (Fig. 3a). However, quantification of METTL3 protein levels relative to the loading control revealed no significant correlation with CRC cell sensitivity to *METTL3* targeting (Fig. 3a).

CRC samples can be classified into distinct subgroups based on the CpG island methylator phenotype (CIMP), an epigenetic signature characterised by hypermethylation of CpG islands in promoter regions of specific genes, categorising them as CIMP-positive or CIMP-negative^38^. CRCs are also commonly classified based on microsatellite status as microsatellite stable (MSS) and microsatellite instability (MSI) subtypes. MSS CRCs exhibit stable microsatellite sequences, whereas MSI CRCs harbour mutations in the DNA mismatch repair system^39,40^. We found no significant correlation between METTL3 dependency and either CIMP status or microsatellite status (MSS/MSI) (Fig. 3b).

We next analysed METTL3 dependency across the four consensus molecular subtypes (CMS) of CRC^41^ (Supplementary Fig. 4a and Supplementary Data 2) and evaluated its relationship with the mutational profiles of CRCs^36^ (Fig. 3c). Although *METTL3*-sensitive cell lines tended to exhibit a higher overall mutation burden, no significant association was observed between *METTL3* sensitivity and CMS classification or mutation status. Together, these findings suggest that CRC sensitivity to *METTL3* targeting is independent of these established molecular features.

To explore potential determinants of differential *METTL3* sensitivity, we examined basal TE expression by RNA-seq in *METTL3*-sensitive (HT29, COLO201, POP92) and *METTL3*-insensitive (HCT116, NCI-H716, CSC73) CRC models. Basal TE expression varied substantially across the models: HT29 and COLO201 displayed higher TE expression than HCT116, whereas NCI-H716 showed even higher TE levels than both HT29 and COLO201 (Fig. 3d–g). Similarly, CSC73 displayed higher TE expression than POP92 PDX CRCs (Supplementary Fig. 4b). SINEs represented the major class of retroelements with elevated expression in *METTL3*-sensitive models compared with insensitive ones. Additional differentially expressed REs included LINEs, LTRs, small RNAs, and other transposable element families (Supplementary Fig. 5a, b). Notably, higher expressed REs in *METTL3*-sensitive cells were particularly enriched within intronic and 3′UTR regions (Supplementary Fig. 5c, d). Furthermore, analysis of Alu subfamilies^42^ revealed that *METTL3*-sensitive cells were enriched for AluS and AluJ subfamilies and depleted of the evolutionarily younger AluY subfamily (Supplementary Fig. 5e, f).

Upon *METTL3*^KD^, HT29, COLO201, and HCT116 cells showed approximately equal numbers of upregulated and downregulated TEs, whereas NCI-H716 displayed a distinct pattern with a greater proportion of upregulated REs (Supplementary Fig. 5g–j). These findings indicate that the impact of m⁶A-dependent post-transcriptional regulation on global TE expression varies across CRC models and is independent of their METTL3 dependency.

Collectively, our findings demonstrate that basal TE expression alone does not account for CRC sensitivity to METTL3 loss. Although two *METTL3*-sensitive models (HT29 and COLO201) express elevated TE levels, one *METTL3*-insensitive model (NCI-H716) shows comparably high TE expression yet fails to form dsRNA upon *METTL3*^KD^. This indicates that factors beyond TE expression, specifically m⁶A levels and dsRNA-forming capacity, determine CRC responses to METTL3 inhibition. Thus, while TE expression varies across CRC models, it is not a reliable determinant of METTL3 dependency.

### METTL3 loss induces transcriptional activation of ISGs in m⁶A-high CRC cells

**Fig. 4:**
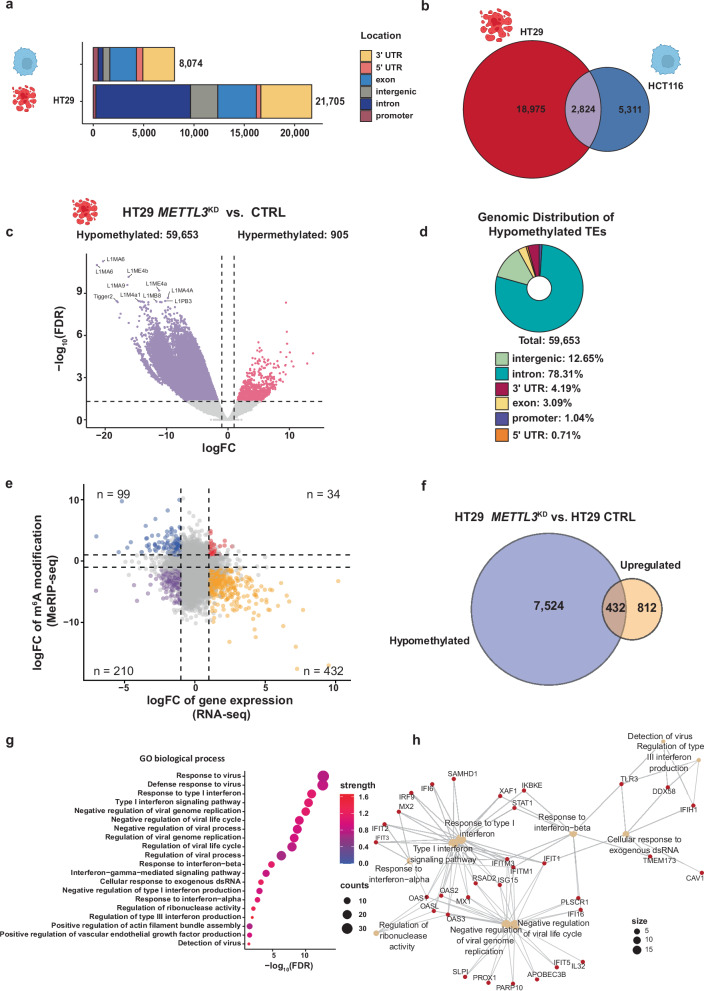
Loss of METTL3 in sensitive CRCs with high basal RNA methylation induces IFN-associated gene expression. **a** Stacked bar plot showing the total number of identified m^6^A peaks and their genomic distribution in HT29 and HCT116 CRC cell lines. **b** Venn diagram showing the number of identified m^6^A peaks based on MeRIP-seq in HT29 (red) and HCT116 (blue) cells. **c** Volcano plot showing changes in transposable element m^6^A RNA modification levels comparing *METTL3*^KD^ with CTRL cells in HT29 cells. (Red, hypermethylated; grey: no significant change; purple: hypomethylated). The data represent three independent experiments. **d** Donut plot showing the genomic distribution of m^6^A-hypomethylated transposable elements comparing *METTL3*^KD^ with CTRL cells in HT29 cells. **e** Scatter plot comparing gene expression changes (RNA sequencing, x-axis) with m^6^A modification changes (MeRIP-seq, y-axis) between *METTL3*^KD^ and CTRL HT29 cells. (Blue, hypermethylated and downregulated genes [*n* = 99]; purple, hypomethylated and downregulated genes [*n* = 210]; red, hypermethylated and upregulated genes [*n* = 34]; orange, hypomethylated and upregulated genes [*n* = 432]; grey, no significant changes). **f** Venn diagram showing the overlap between hypomethylated genes identified by MeRIP-seq and upregulated genes identified by RNA sequencing. **g** Enrichment analysis showing the top 20 Gene Ontology (GO) biological process terms derived from the 432 hypomethylated and upregulated genes in HT29 *METTL3*^KD^ compared with CTRL cells. The size of each GO term corresponds to the number of associated genes, while the colour represents the enrichment strength. **h** Gene–concept network illustrating the top 10 enriched pathways among the 432 hypomethylated and upregulated genes in HT29 *METTL3*^KD^ compared with CTRL cells. Source data are provided as a Source Data file.

To further investigate the mechanisms underlying the variable sensitivity of CRC cells to *METTL3* targeting, we assessed global m⁶A modification levels following targeting *METTL3* across all CRC models using dot-blot analysis on total RNA using an anti-m^6^A antibody. METTL3 inhibition significantly reduced global m^6^A modifications across all assessed CRC models (Supplementary Fig. 5k), excluding the possibility of compensatory METTL3 catalytic activity as a cause of differential sensitivity. To quantify and map the m^6^A modifications, we performed methylation RNA Immunoprecipitation sequencing (MeRIP-Seq) on total RNA from HCT116 and HT29 cells (Supplementary Fig. 5l). In both cell lines, m⁶A peaks were enriched for the canonical DRACH motif (Supplementary Fig. 5m). Notably, HT29 cells exhibited a substantially higher number of m⁶A peaks compared to HCT116 cells, with 21,705 peaks identified in HT29 versus 8,074 in HCT116 (Fig. 4a, b). Consistent with the elevated expression of intronic REs in HT29 relative to HCT116 (Supplementary Fig. 5c), a large fraction of HT29 m⁶A peaks (43.23%, corresponding to 9390 peaks) mapped to intronic regions (Fig. 4a).

To explore the role of m^6^A modification in regulating the immunostimulatory activity of TE-derived RNAs, we assessed the changes in m^6^A methylation on TE transcripts following *METTL3* targeting. Consistent with the global reduction in m⁶A levels, we identified 59,653 hypomethylated TE transcripts upon *METTL3*^KD^ (Fig. 4c). The majority of hypomethylated TEs were LINEs, SINEs and LTRs, predominantly located within intronic regions (78.31%) (Fig. 4d and Supplementary Fig. 6a).

Since m^6^A modification has been associated with promoting RNA decay^22,43^, we investigated whether the increased abundance of ISG transcripts in METTL3-deficient cells might reflect altered RNA stability following loss of m^6^A. By integrating RNA-seq and MeRIP-seq datasets, we identified 432 genes in HT29 cells that were both upregulated and hypomethylated upon *METTL3* depletion (Fig. 4e, f). GO and network analysis revealed that these genes were enriched for antiviral responses and Type I IFN signalling (Fig. 4g, h). These findings suggest that the loss of m^6^A may contribute to the elevated expression of ISGs in METTL3-deficient cells by enhancing transcript stability.

**Fig. 5:**
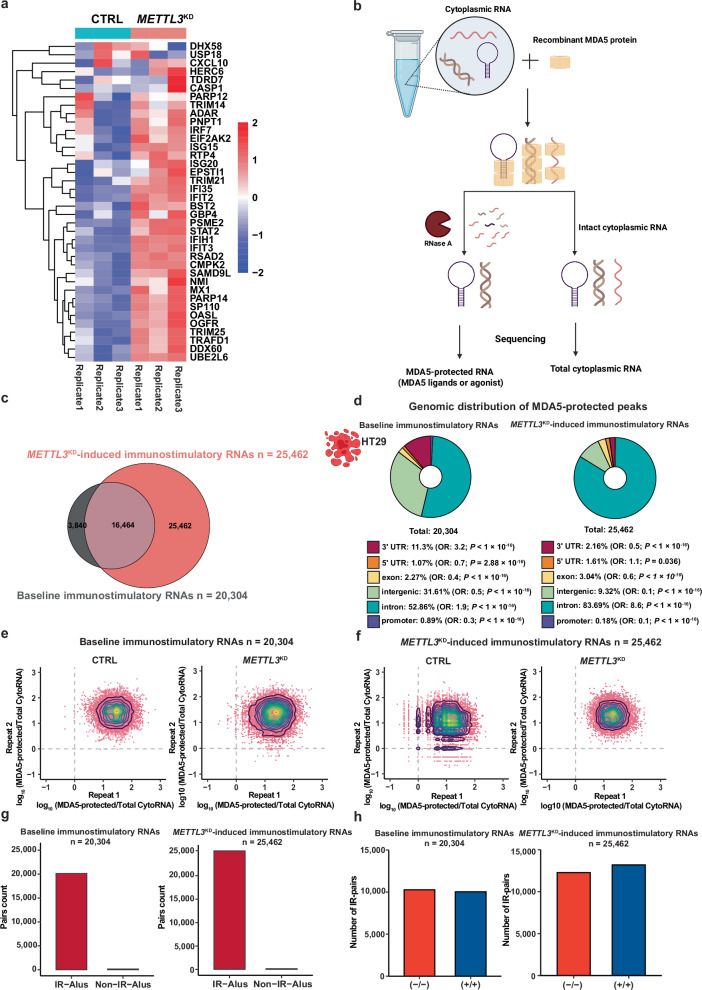
METTL3 depletion induces the accumulation of immunostimulatory dsRNA derived from IR-Alu repeats. **a** Heatmap showing H3K4me3 occupancy at the transcription start site (TSS) of ISG38, based on CUT&RUN analysis (Z score), using a colour scale ranging from dark blue (low occupancy) to red (high occupancy) in HT29 *METTL3*^KD^ compared with CTRL cells. The data represent three independent experiments. **b** Schematic representation of the RNase A/MDA5 protection assay. To identify the ligand of MDA5, total cytoplasmic RNA was purified from CTRL or *METTL3*^KD^ HT29 cells and incubated with recombinant MDA5-Δ2CARD protein. Samples were either left intact or digested with RNase A. RNA purified from both conditions was then processed for library preparation and RNA sequencing. Created in BioRender. Mehdipour, P. (2026) https://BioRender.com/c4n9bbi. **c** Venn diagram showing the number of immunostimulatory RNAs at baseline (*n* = 20,304) and under *METTL3*^KD^-induced conditions (*n* = 25,462). **d** Genomic distribution of baseline immunostimulatory RNAs (*n* = 20,304) and *METTL3*^KD^-induced immunostimulatory RNAs (*n* = 25,462). The odds ratio (OR) indicates enrichment or depletion within each genomic category relative to the distribution of all IR-Alu pairs in the human genome. Statistical significance was determined using a two-sided Fisher’s exact test. **e**,** f** Scatter plots showing the log₁₀-transformed fold change of MDA5-protected relative to total cytoplasmic RNA (cytoRNA) for each repeat within an IR-Alu pair. The y-axis represents the fold change of repeat 1, and the x-axis represents the fold change of repeat 2 within each pair. Data are shown for baseline immunostimulatory RNAs (*n* = 20,304) (**e**) and *METTL3*^KD^-induced immunostimulatory RNAs (*n* = 25,462) (**f**). **g** Bar plot showing the number of baseline immunostimulatory RNAs and *METTL3*^KD^-induced immunostimulatory RNAs derived from IR-Alu or non–IR-Alu elements. **h** Transcriptional orientation of IR-Alu pairs identified as immunostimulatory at baseline (*n *= 20,304) and under *METTL3*^KD^ conditions (*n* = 25,462). ( − /−, red) indicates both repeats are on the antisense strand, whereas (+/+, blue) indicates both repeats are on the sense strand. Source data are provided as a Source Data file.

To directly assess whether ISG expression was transcriptionally induced, we performed genome-wide CUT&RUN (cleavage under targets and release using nuclease)^44^ to measure the active promoter mark histone 3 lysine 4 trimethylation (H3K4me3). CUT&RUN analysis revealed altered changes in H3K4me3 occupancy for numerous genes in *METTL3*^KD^ relative to CTRL, with 2779 genes showing increased, and 50 genes showing decreased H3K4me3 deposition at their transcription start sites (TSS) (Supplementary Fig. 6b, c). In particular, the majority of the 38 ISGs upregulated in *METTL3*^KD^ HT29 cells also showed increased H3K4me3 occupancy at their TSS (Fig. 5a and Supplementary Fig. 6d), consistent with transcriptional activation. Moreover, *MAVS*^KO^ completely abolished ISG induction following *METTL3*^KO^ (Fig. 2h), demonstrating that METTL3 loss activates ISG transcription through MAVS-dependent innate immune signalling rather than through changes in RNA stability. Collectively, these data support a model in which *METTL3* depletion induces antiviral transcriptional programmes, highlighting the central role of transcriptional regulation in the observed anti-tumour immune gene-expression signature.

### METTL3 depletion induces IR-Alu immunostimulatory dsRNAs

To directly characterise endogenous immunostimulatory dsRNAs induced by *METTL3*^KD^, we performed an RNaseA/MDA5 protection assay^13,45^, followed by RNA-seq using cytoplasmic RNA from HT29 cells (Fig. 5b). This assay identified immunostimulatory dsRNAs protected by recombinant MDA5 protein. In HT29 cells, 92% and 94% of MDA5-protected regions in CTRL and *METTL3*^KD^, respectively, overlapped with repeat elements (Supplementary Fig. 7a). SINEs, particularly Alu elements, constituted the majority of MDA5-protected regions, and *METTL3*^KD^ resulted in a 1.7-fold increase in the total number of MDA5-protected repeat elements (73,134 vs. 42,418) (Supplementary Fig. 7b).

To explore how REs generate immunostimulatory dsRNA, we searched for repeat pairs within 3 kb that could form complementary structures. This analysis identified 20,304 repeat pairs capable of forming dsRNAs in CTRL cells, most of which (81%) overlapped with the 41,926 pairs detected in *METTL3*^KD^ cells. We categorised the 20,304 repeat pairs as baseline immunostimulatory RNAs and the 25,462 repeat pairs uniquely detected in *METTL3*^KD^ as *METTL3*^KD^-induced immunostimulatory RNAs (Fig. 5c). The baseline immunostimulatory RNAs were distributed across intronic and intergenic regions, whereas 84% of *METTL3*^KD^-induced immunostimulatory RNAs were localised to intronic regions (Fig. 5d). Although the expression of baseline immunostimulatory RNA was comparable between CTRL and *METTL3*^KD^ conditions, *METTL3*^KD^ significantly enhanced MDA5 protection of immunostimulatory dsRNAs without necessarily increasing their transcription (Fig. 5e, f and Supplementary Figs. 7c, d, 8a–d). Importantly, nearly all repeat pairs in both conditions corresponded to IR-Alus (Fig. 5g), and both baseline IR-Alus and *METTL3*^KD^-induced IR-Alus were enriched for the AluS subfamilies (Supplementary Fig. 8e). These immunostimulatory dsRNAs were predominantly unidirectionally transcribed in either sense (+/+) or antisense (-/-) orientations (Fig. 5h), a configuration consistent with the formation of intramolecular stem-loop structures. Together, these findings suggest that METTL3 inhibition enhances the formation or persistence of IR-Alu–derived dsRNA structures in CRC cells.

**Fig. 6:**
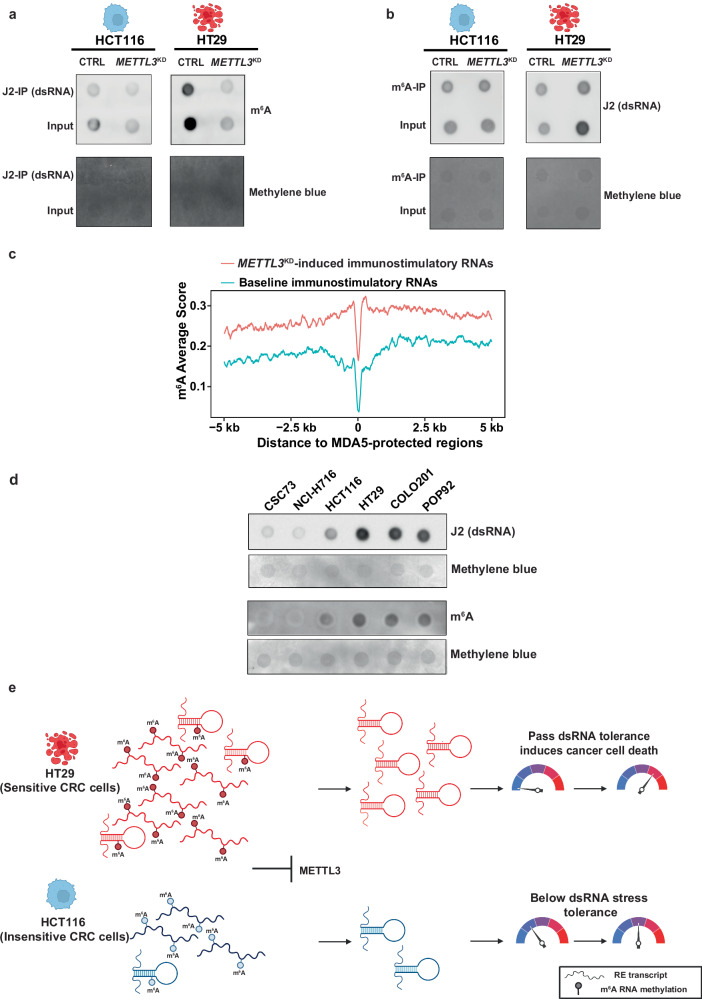
Basal m⁶A and dsRNA levels determine METTL3-dependent viral mimicry responses in CRC. **a** Dot blot analysis showing m^6^A levels in total RNA (input) isolated from HCT116 and HT29 CTRL and *METTL3*^KD^ cells, and in RNA immunoprecipitated (IP) with an anti-J2 antibody (dsRNA). Methylene blue staining was used as a loading control. Representative dot blots from three independent experiments. **b** Dot blot analysis showing dsRNA levels in total RNA (input) isolated from HCT116 and HT29 CTRL and *METTL3*^KD^ cells and in RNA immunoprecipitated (IP) with an anti-m^6^A antibody. Methylene blue staining was used as a loading control. **c** Average m^6^A methylation levels flanking ( − 5 kb to + 5 kb; upstream and downstream) baseline MDA5-protected regions (cyan) and *METTL3*^KD^-induced MDA5-protected regions (salmon). **d** Dot blot analysis showing double-stranded RNA (dsRNA; detected with the J2 antibody) and m⁶A levels in total RNA isolated from the indicated CRC cell lines and patient-derived xenografts (PDXs). Methylene blue staining was used as a loading control. Representative dot blots from two independent experiments. **e** Schematic representation of the proposed model based on our findings. *METTL3*-sensitive CRC cells exhibit higher basal levels of dsRNA and increased m⁶A modification. Upon *METTL3* targeting, loss of m⁶A promotes the accumulation of immunostimulatory dsRNA, enabling cells to surpass the dsRNA stress tolerance threshold and trigger viral mimicry–associated cancer cell death. In contrast, *METTL3*-insensitive CRC cells display lower basal dsRNA levels and reduced m⁶A modification. Following *METTL3* targeting, dsRNA accumulation induces sublethal interferon (IFN) responses that remain below the dsRNA stress tolerance threshold, thereby failing to activate viral mimicry responses or induce cancer cell death. Created in BioRender. Mehdipour, P. (2026) https://BioRender.com/z6j69x4. Source data are provided as a Source Data file.

Of note, higher basal TE expression in CRC cells does not always correlate with increased ISG expression, as seen in HT29 and NCI-H716 cells (Supplementary Fig. 2f). This suggests that expressed REs do not necessarily form immunostimulatory dsRNA structures. Our results suggest that this phenomenon depends, at least in part, on METTL3 activity, whereby METTL3-mediated m⁶A modifications suppress the formation of dsRNA from transcriptionally active REs. To test this hypothesis, we measured relative m⁶A methylation levels within dsRNA by dot blot analysis of total RNA (input) and RNA immunoprecipitated with the J2 dsRNA antibody (J2-IP) (Fig. 6a). In both HT29 and HCT116 cells, we observed reduced m⁶A levels in J2-IP samples compared to total RNA, indicating that dsRNAs in the total RNA pool are relatively hypomethylated. This observation was further supported by analysis of dsRNA abundance in total RNA compared to RNA immunoprecipitated with an anti-m⁶A antibody (m⁶A-IP). In HT29 cells, *METTL3*^KD^ markedly increased dsRNA abundance in total RNA, whereas this induction was substantially attenuated in the m⁶A-methylated fraction (Fig. 6b). These observations suggest that transcripts containing m⁶A modifications are less likely to form or maintain dsRNA structures, and that loss of m⁶A increases the availability of RNAs that can fold into dsRNA.

Consistently, mapping m⁶A density across MDA5-protected regions revealed that *METTL3*^KD^-induced MDA5-bound repeat-derived dsRNAs arise from genomic loci that are normally enriched for m⁶A (Fig. 6c), demonstrating increased abundance and/or accessibility of long dsRNA species. These effects occur without detectable changes in RNA half-life for the selected repeat-derived transcripts, as measured by Actinomycin D chase followed by RT–qPCR (Supplementary Fig. 9a), indicating that these changes are unlikely to result from altered RNA stability.

Although RNAfold did not predict global minimum-free-energy differences between methylated and unmethylated sequences for the selected sequences (Supplementary Fig. 9b–d), this is consistent with prior evidence that m⁶A primarily influences local and kinetic RNA folding rather than global thermodynamics. NMR and biochemical studies have shown that m⁶A weakens base stacking and destabilises local duplex structures^46–48^, supporting the idea that loss of m⁶A favours the formation or persistence of dsRNA conformations. The stem-loop structures inferred from the RNase A/MDA5 protection assay are, consistent with intramolecular pairing of convergently oriented IR-Alu elements.

Dot blot analysis further showed higher basal dsRNA and m⁶A levels in *METTL3*-sensitive compared with *METTL3*-insensitive CRC models (Fig. 6d). Collectively, these findings support a model in which *METTL3*-sensitive CRCs, characterised by higher m⁶A deposition and capacity to generate RE-derived dsRNAs, accumulate higher levels of dsRNA following *METTL3*^KD^, thereby triggering viral mimicry and cancer cell death. In contrast, *METTL3*-insensitive CRCs, despite sometimes exhibiting high basal TE expression, display low m⁶A levels and fail to generate any or sufficient dsRNA upon *METTL3*^KD^ to surpass the threshold required for antiviral activation, and therefore do not undergo viral mimicry–mediated cytotoxicity (Fig. 6e).

### DNMT inhibition combined with METTL3 depletion overcomes resistance and reduces CRC tumour burden in vivo

Given that a subset of CRCs are insensitive to METTL3 targeting due to reduced m⁶A modification and contain lower basal levels of dsRNAs, thereby failing to accumulate sufficient immunostimulatory dsRNAs to surpass the dsRNA stress threshold (Fig. 6e), we treated *METTL3*-insensitive HCT116 cells with Decitabine (DAC), a DNMTi known to induce dsRNAs and viral mimicry responses in CRCs^6,13^. The rationale for this combination is supported by multiple studies demonstrating that DNMTi treatment induces endogenous dsRNA transcription and triggers viral mimicry across diverse cancer types^6,7,49^. In addition, early clinical studies have shown that combining DNMT inhibition with immune-checkpoint blockade, through viral mimicry, can enhance therapeutic responses in patients with refractory cancers, including reports of high complete-response rates in immunotherapy-resistant NK/T-cell lymphoma^50^.

**Fig. 7:**
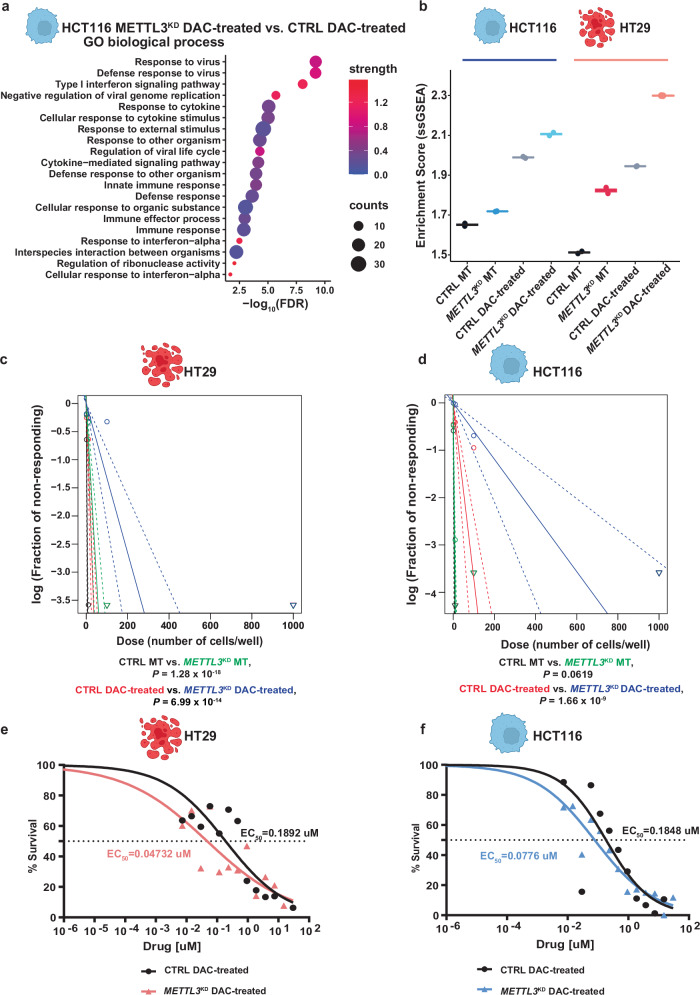
Combined DNMT inhibition and *METTL3* targeting in *METTL3*-insensitive CRC cells induces ISG expression and reduces cancer-initiating cell frequency. **a** Enrichment analysis showing the top 20 Gene Ontology (GO) biological process terms derived from the 178 significantly upregulated genes in DAC-treated HCT116 *METTL3*^KD^ cells compared with DAC-treated CTRL cells. The size of each GO term corresponds to the number of associated genes, while the colour represents the enrichment strength. **b** ISG38 single-sample GSEA (ssGSEA) scores in HT29 and HCT116 cells under the following conditions: control mock-treated (CTRL MT), *METTL3*^KD^ mock-treated *(METTL3*^KD^ MT), control DAC-treated (CTRL DAC-treated), and *METTL3*^KD^ DAC-treated (*METTL3*^KD^ DAC-treated) (*n* = 2 biological replicates). **c** Cancer-initiating cell (CIC) frequency determined by in vitro limiting dilution assay (LDA) of HT29 CRC cells under the indicated conditions. CTRL MT vs *METTL3*^KD^ MT, *P* = 1.28 × 10⁻¹⁸; CTRL DAC-treated vs *METTL3*^KD^ DAC-treated, *P* = 6.99 × 10⁻¹⁴. Statistical significance was determined using a two-sided chi-square test. **d** Cancer-initiating cell (CIC) frequency determined by in vitro limiting dilution assay (LDA) of HCT116 CRC cells under the indicated conditions. CTRL MT vs *METTL3*^KD^ MT, *P* = 0.0619; CTRL DAC-treated vs *METTL3*^KD^ DAC-treated, *P* = 1.66 × 10⁻⁹. Statistical significance was determined using a two-sided chi-square test. **e** Survival of CTRL (wild-type; black) and *METTL3*^KD^ (salmon) HT29 CRC cells following treatment with decitabine (DAC). Luminescence signals were normalised, and dose–response curves and EC50 values were calculated using nonlinear regression. **f** Survival of CTRL (wild-type; black) and *METTL3*^KD^ (blue) HCT116 CRC cells following treatment with decitabine (DAC). Luminescence signals were normalised, and dose–response curves and EC50 values were calculated using nonlinear regression. Source data are provided as a Source Data file.

Combining METTL3 depletion with DAC treatment significantly upregulated 178 genes in HCT116 cells, with the top enriched GO biological processes related to antiviral pathways and type I interferon signalling (Fig. 7a and Supplementary Fig. 10a). RNA-seq further revealed that this combination enhanced induction of the ISG38 signature in both *METTL3*-insensitive HCT116 and *METTL3*-sensitive HT29 cells (Fig. 7b), a finding that was further validated by qPCR analysis of representative ISGs, including *IRF7*, *ISG15*, and *MX1* (Supplementary Fig. 10b, c).

Consistent with these transcriptomic changes, CellTiter-Glo and colony-formation assays demonstrated that DAC treatment enhanced the anti-tumour effects of METTL3 inhibition in both *METTL3*-sensitive and *METTL3*-insensitive CRC models (Supplementary Fig. 10d–h), suggesting that DNMT inhibition can overcome resistance to *METTL3* targeting.

We next investigated whether this combination affects cancer-initiating cell (CIC) frequency. In HT29 cells, *METTL3* targeting alone reduced CIC frequency, and the combination with DAC further enhanced this effect. In contrast, CIC frequency in HCT116 cells was unaffected by *METTL3*^KD^ alone but was significantly reduced when METTL3 inhibition was combined with DAC (Fig. 7c, d). *METTL3*^KD^ also decreased the half-maximal effective concentration (EC50) of DAC by 4-fold in HT29 cells and 2.3-fold in HCT116 cells (Fig. 7e, f).

**Fig. 8:**
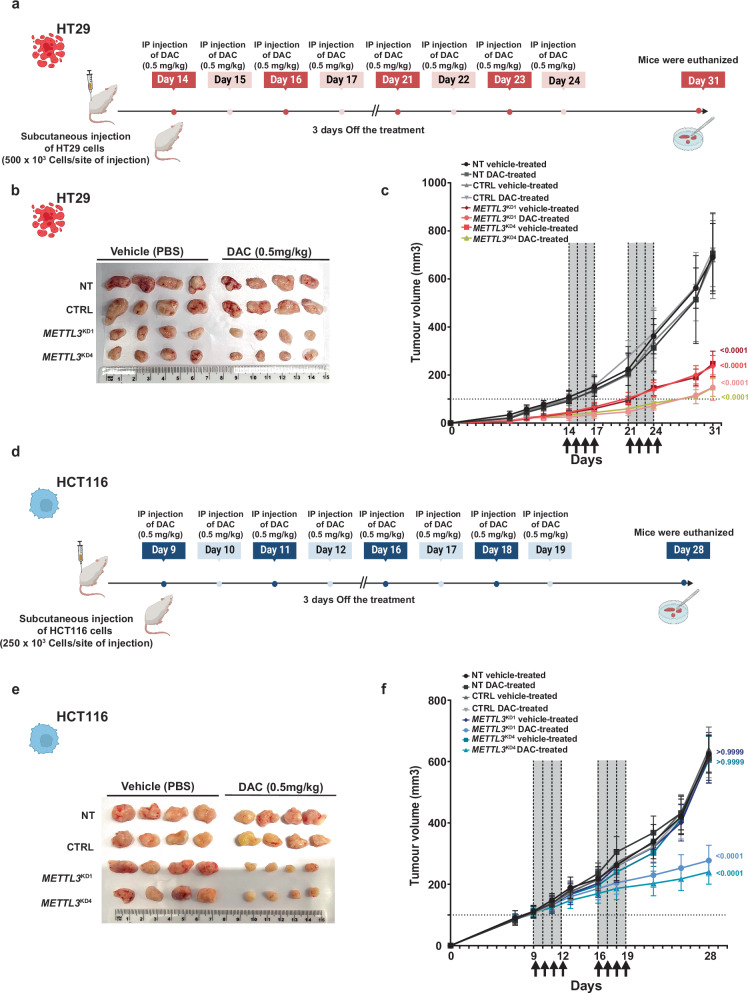
DNMT inhibition enhances the anti-tumour efficacy of *METTL3* targeting. **a** Schematic representation of the in vivo experimental design. Non-transduced (NT), control-transduced (CTRL), *METTL3*^KD1^, and *METTL3*^KD4^ HT29 CRC cells were injected subcutaneously into the flanks of NSG mice (*n* = 14 tumours for NT and CTRL vehicle-treated groups; *n* = 16 tumours for all other groups). Mice were treated on the indicated days with 0.5 mg kg⁻¹ decitabine (DAC) by intraperitoneal injection or vehicle control (PBS) for four consecutive days, followed by a three-day break and a second four-day treatment cycle. Created in BioRender. Mehdipour, P. (2026) https://BioRender.com/jzltuec. **b** HT29 tumours dissected from vehicle- and DAC-treated cohorts on day 7 after the final treatment are shown for each condition (*n* = 4). Representative tumours are shown. **c** HT29 tumour volume was measured at the indicated time points. Dashed lines indicate treatment days. *P-*values denote comparisons at the final time point between *METTL3*^KD1^ or *METTL3*^KD4^ and NT/CTRL within vehicle-treated groups, and between *METTL3*^KD1^ or *METTL3*^KD4^ and NT/CTRL within DAC-treated groups. Data are presented as mean ± SD. Statistical significance was determined by two-way ANOVA with Sidak’s multiple-comparisons correction. **d** Schematic representation of the in vivo experimental design. NT, CTRL, *METTL3*^KD1^, and *METTL3*^KD4^ HCT116 CRC cells were injected subcutaneously into the flanks of NSG mice (*n* = 16 tumours per group). Mice were treated on the indicated days with 0.5 mg kg⁻¹ DAC by intraperitoneal injection or vehicle control (PBS) for four consecutive days, followed by a three-day break and a second four-day treatment cycle. Created in BioRender. Mehdipour, P. (2026) https://BioRender.com/71bv37i. **e** HCT116 tumours dissected from vehicle- and DAC-treated cohorts on day 9 after the final treatment are shown for each condition (*n* = 4). **f** HCT116 tumour volume was measured at the indicated time points. Dashed lines indicate treatment days. *P-*values denote comparisons at the final time point between *METTL3*^KD1^ or *METTL3*^KD4^ and NT/CTRL within vehicle-treated groups, and between *METTL3*^KD1^ or *METTL3*^KD4^ and NT/CTRL within DAC-treated groups. Data are presented as mean ± SD. Statistical significance was determined by two-way ANOVA with Sidak’s multiple-comparisons correction. Source data are provided as a Source Data file.

To further evaluate the therapeutic potential of *METTL3* targeting alone or in combination with DAC in vivo, we injected non-transduced (NT), control (CTRL) and *METTL3*^KD^, HT29 and HCT116 cells into the flanks of immunodeficient NSG mice. Once tumours in the CTRL groups reached approximately 100 mm³, mice were treated with DAC (0.5 mg/kg) in two cycles of 4 days, with a 3-day break between cycles (Fig. 8a, d). Low-dose DAC treatment alone had no significant effect on tumour growth in either models, consistent with clinical trial results for DAC in solid tumours^51,52^. In *METTL3*-sensitive HT29 cells, *METTL3*^KD^ alone demonstrated significant anti-tumour effects (Fig. 8b, c and Supplementary Fig. 11a). In contrast, *METTL3*^KD^ HCT116 cells grew similarly to CTRL cells in vehicle-treated mice (Fig. 8e, f and Supplementary Fig. 11b). Importantly, DAC treatment in combination with *METTL3*^KD^ in HCT116 cells exhibited significant anti-tumour activity. Together, these findings suggest that cancer therapies such as DAC, which increase the accumulation of endogenous dsRNAs, can sensitise CRCs to *METTL3* targeting, highlighting a potential combinatorial approach for more effective CRC treatment.

In summary, our findings indicate that METTL3 targeting induces viral mimicry-driven tumour cell killing in a subset of CRCs characterised by high m⁶A deposition and the capacity to generate dsRNA, whereas CRCs with limited dsRNA-forming ability are resistant. However, increasing endogenous dsRNA levels through DNMT inhibition can sensitise these resistant models to METTL3 targeting, highlighting a promising combinatorial strategy for CRC therapy.

## Discussion

While the role of METTL3 has been examined in CRC^30,31,53,54^, its contribution to innate immune regulation and viral mimicry has remained unclear. Here, we identify a potential mechanism by which CRC cells evade antiviral immunity and demonstrate that *METTL3* dependency varies widely across CRC models. In *METTL3*-sensitive CRCs, METTL3 inhibition triggers robust antiviral signalling and tumour suppression in vivo. In contrast, *METTL3*-insensitive CRCs mount only minimal or sub-lethal type I IFN responses, necessitating additional elevation of endogenous dsRNA, such as through DNMT inhibition, to restore viral mimicry activation and cell death.

A key determinant of this differential sensitivity is the m⁶A-dependent capacity of retroelement-derived transcripts to form dsRNA. *METTL3*-sensitive CRCs are characterised by high global m⁶A deposition and preserved dsRNA-forming competence, enabling METTL3 loss to promote accumulation of dsRNAs derived largely from intronic IR-Alu elements. These elements are normally maintained in a non-immunogenic state through extensive m⁶A methylation; METTL3 depletion allows these transcripts to fold or persist as dsRNA, activating MAVS, inducing ISGs, and triggering PKR-dependent cell death. Conversely, *METTL3*-insensitive CRCs, including those with high basal TE expression, exhibit low m⁶A deposition and limited dsRNA-forming capacity, highlighting that transcriptional output alone does not dictate METTL3 sensitivity.

Mechanistically, our findings suggest how m⁶A influences the structural fate of IR-Alu–containing transcripts. Biochemical and structural studies have shown that m⁶A destabilises RNA duplexes by disrupting base stacking and altering annealing kinetics^46,55,56^, providing a biophysical rationale for how loss of m⁶A may promote the formation or persistence of IR-Alu–derived dsRNAs. Although global RNA structure-mapping approaches such as DMS-seq or SHAPE-seq could further refine this model^57,58^, these methods cannot readily distinguish immunostimulatory IR-Alu dsRNAs from other structured RNAs. In this context, our RNase A/MDA5 protection assay is particularly informative, as it selectively enriches dsRNAs capable of engaging innate immune sensors.

We note that our data resolve steady-state endogenous dsRNA abundance and associated signalling outputs, but do not partition the upstream kinetic contributions of transcript production versus decay. Given established m⁶A reader–mediated pathways, including YTHDF- and IGF2BP-dependent mechanisms that regulate RNA stability and turnover, altered decay kinetics may plausibly contribute to the observed dsRNA changes^22,43,59^; however, our focus is on the biologically decisive endpoint, whether repeat-derived dsRNAs surpass an abundance and structural threshold sufficient to engage innate immune sensing and downstream effectors in CRC.

Our data further indicate that ISG induction following METTL3 loss is driven primarily by the sensing of accumulated dsRNA rather than by altered stability of immune transcripts. Increased H3K4me3 occupancy at ISG promoters, the immunostimulatory activity of RNA from METTL3-depleted cells, and the loss of ISG induction in MAVS-deficient cells collectively demonstrate that dsRNA-mediated antiviral signalling is the dominant pathway. Although METTL3 participates in additional RNA-metabolic processes that may also contribute to the observed phenotype, the substantial rescue of cytotoxicity in PKR/METTL3 double-knockout cells highlights viral mimicry as the principal driver of cell death.

Gao et al. previously demonstrated that METTL3 loss during murine fetal haematopoiesis leads to the accumulation of dsRNAs derived predominantly from protein-coding transcripts^27^. While these findings highlight a conserved role for METTL3 in limiting dsRNA formation, our results identify a mechanistically distinct source of immunogenic dsRNA in human CRC. Through integration of m⁶A profiling with RNase A/MDA5 protection assays, we show that IR-Alu elements constitute the primary MDA5-engaging dsRNAs activated upon METTL3 loss, an outcome likely influenced by both the differing biological contexts and the primate-specific expansion of Alu repeats. We further demonstrate substantial heterogeneity across CRC models in their capacity to generate dsRNA and show that DNMT inhibition, a known activator of retroelement dsRNA, synergises with METTL3 targeting to overcome resistance.

Consistent with our findings, a recent study by Li et al. reported that METTL3 deficiency in mammary epithelial cells leads to enrichment of retroelement-derived transcripts, including Alu, LINE, and ERV RNAs, and elicits a type I interferon response^60^. This observation in an orthogonal epithelial model supports a conserved role for METTL3 in restraining repeat-derived immunostimulatory RNA across tissues. Our work extends these findings by identifying IR-Alu elements as the predominant source of MDA5-engaging dsRNA in human CRC and by demonstrating that *METTL3* dependency is governed not simply by retroelement expression, but by the m⁶A-dependent structural competence of repeat-derived transcripts to form dsRNA. Together, these studies highlight repeat-derived RNA as a recurrent substrate of METTL3-mediated immune evasion, while underscoring context-specific differences in the nature and immunogenic potential of these transcripts.

These mechanistic insights have direct therapeutic implications. METTL3 inhibitors, such as the clinical-stage compound STC-15 (NCT05584111), are currently being evaluated for the treatment of solid tumours^29^. Our results suggest that their effectiveness in CRC will depend on the basal m⁶A landscape and dsRNA-forming potential of IR-Alu transcripts. To overcome this challenge and extend the therapeutic potential of METTL3 inhibitors for the treatment of solid tumours, our findings provide a rationale for combining METTL3 targeting with DNMT inhibitors and, potentially, other approaches that elevate immunostimulatory dsRNA levels, including pharmacologic inhibition of CDK4/6, LSD1, or G9a, as well as genetic perturbation of retroelement-silencing factors such as SETDB1 or the exonuclease XRN1^10,11,49,61–64^. However, the therapeutic potential of these additional combinations needs to be further explored and experimentally validated.

Finally, our findings provide a unifying model in which CRC susceptibility to *METTL3* targeting is determined not by retroelement expression alone, but by the m⁶A-dependent structural competence of RE-derived transcripts to form immunostimulatory dsRNA. CRCs with high global m⁶A harbour a substantial pool of IR-Alu transcripts poised for dsRNA formation upon METTL3 loss, whereas low-m⁶A CRCs lack this capacity and remain resistant. Although these conclusions are supported by cell-line and PDX models, further evaluation in primary CRC specimens will be essential to establish clinical relevance and to develop biomarkers that predict response to METTL3-targeted therapies, alone or in combination with DNMT inhibition.

## Methods

### Cell lines and culture conditions

HT29 cells (HTB-38™) were kindly given to us by the group of Prof. Colin Goding at the Ludwig Institute for Cancer Research, Oxford, HCT116 (CCL-247™), COLO201(CCL-224™), NCI-H716 (CCL-251™) and CCD 841 CoN Normal colon cells (CRL-1790™) were purchased from ATCC. HT29 and HCT116 human colorectal cancer cell lines were cultured in McCoy’s 5 A medium supplemented with penicillin-streptomycin (1%), and Fetal-Bovine-serum (10%). COLO201 and NCI-H716 human colorectal cancer cell lines were cultured in RPMI-1640 medium supplemented with penicillin-streptomycin (1%) and Fetal-Bovine-serum (10%). CCD 841 CoN (Normal colon) cells were cultured in ATCC-EMEM, supplemented with penicillin-streptomycin (1%), and Fetal-Bovine-serum (10%).

HT29 cells were authenticated by Eurofins Genomics using PCR-based single-locus technology. Sixteen independent STR loci (D8S1179, D21S11, D7S820, CSF1PO, D3S1358, TH01, D13S317, D16S539, D2S1338, AMEL, D5S818, FGA, D19S433, vWA, TPOX and D18S51) were analysed. Cells tested negative for mycoplasma contamination. In addition, ATCC performs STR profiling for cell line authentication and conducts mycoplasma testing prior to distribution.

A complete list of reagents and kits used in this study, including supplier information and catalogue numbers, is provided in Supplementary Data 3.

### Patient-derived xenograft colorectal cancer cells

Human colorectal tumour specimens were collected at the time of surgical resection following informed patient consent under protocols approved by the Research Ethics Board of the University Health Network (Toronto, Canada). Patient-derived xenograft (PDX) models of colorectal cancer were generously provided by Dr. Catherine O’Brien’s laboratory. These xenografts were generated by subcutaneous implantation of tumour cells into the flanks of 6–8-week-old NOD-SCID mice^65^. Tumour-derived cells were maintained as spheroids in DMEM/F-12 medium supplemented with penicillin-streptomycin (1%) (Thermo Fisher), 1x nonessential amino acids, 1 mM sodium pyruvate, N2 supplement, NeuroCult SM1 Neuronal Supplement, heparin (4 μg ml−1), 0.2% lipid mixture, epidermal growth factor (EGF; 20 ng ml−1) and basic fibroblast growth factor (bFGF; 10 ng ml−1). Cells were authenticated using short tandem repeat profiling and tested negative for mycoplasma.

### Pharmacological treatment

The DNA methyltransferase inhibitor, Decitabine (DAC; 5-Aza-2’-deoxycytidine) was reconstituted in PBS as 10 mM stocks and stored at − 80 °C. For in vitro experiments, cells were seeded 24 hours before treatment at 50,000 cells/mL and then treated with Decitabine (300 nM) or mock-treated. Cells were kept in culture for 5 days. Cells were then collected and used for downstream analysis.

### Generation of *METTL3* knock-down cells

Lentiviral vectors containing shRNA (^KD1/KD2/KD3/KD4^) targeting human *METTL3* with turbo green fluorescent protein (TurboGFP) as CTRL were generated from pLKO1 Plasmids purchased from Sigma-Aldrich. The shRNA sequences are shown in Supplementary Data 4. The viral particles were produced by calcium phosphate transfection^66^ and concentrated using polyethylene glycol (PEG). In brief, *METTL3*^KD^ and wild-type (CTRL) CRC cells were generated by the transduction of shRNA-based lentiviral particles. CRCs were seeded into 12-well plates (100,000 cells per mL) and transduced with 30 μL of 200x concentrated viruses. 72 h after the transduction, GFP^+^ cells were sorted using a Sony MA900 sorter or selected with puromycin at 2 µg ml−1, and knock-down of METTL3 was confirmed at mRNA and protein levels by qPCR and western blot, respectively.

### RNA extraction and qPCR

CTRL (TGFP) or/and *METTL3*^KD1/2/3/4^ colorectal cancer cells were treated with Decitabine (300 nM) or mock-treated (MT). 5 days post-treatment, the cells were collected, washed in PBS and used for RNA extraction. Total RNA was extracted with the RNeasy Mini Kit following the manufacturer’s protocol. Briefly, after adding RLT buffer and 70% ethanol to the cell pellets, in-column DNase I digestion was performed using the QIAGEN RNase-Free DNase Set (catalogue 79256). Total RNA was quantified using a Nanodrop 2000 spectrophotometer, and samples with A260/A280 ratios ≥ 1.8 were considered suitable for downstream applications. For RNA sequencing, 500 ng of total RNA was used for library preparation. For cDNA synthesis, 500 ng to 1 µg of RNA was reverse-transcribed using SuperScript IV VILO cDNA Synthesis Kit according to the manufacturer’s instructions. Quantitative PCR (qPCR) was carried out in triplicate using 10–20 ng of cDNA per 20 µL reaction, consisting of 5 µL cDNA template, 10 µL SYBR Select Master Mix, and 0.2 µM each of forward and reverse primers. Amplification was performed on a StepOnePlus Real-Time PCR System (Applied Biosystems). Relative mRNA expression levels were normalised to GAPDH and expressed relative to the control group. Primer sequences are provided in Supplementary Data 5.

### Library preparation for total RNA sequencing

Total RNA-sequencing libraries were prepared at the Oxford Genomics Centre (OGC) or by GENEWIZ® (Azenta Life Sciences). For samples processed at OGC, RNA concentration was measured using the RiboGreen assay (Invitrogen) on a FLUOstar OPTIMA plate reader (BMG Labtech), and RNA integrity was assessed using the Agilent 2200 or 4200 TapeStation with RNA ScreenTape. RNA integrity numbers (RIN) ranged from 5.5 to 10. One hundred nanograms of total RNA was used as input for library preparation. Ribosomal RNA was depleted using the NEBNext rRNA Depletion Kit (Human/Mouse/Rat; New England Biolabs), and strand-specific libraries were generated using the NEBNext Ultra II Directional RNA Library Prep Kit for Illumina. Libraries were amplified (14 cycles), quantified, normalised, pooled, and prepared for sequencing according to standard protocols. Paired-end sequencing was performed on an Illumina NovaSeq 6000 platform (Illumina; NovaSeq 6000 S4 reagent kit, 300 cycles). Samples processed by GENEWIZ® (Azenta Life Sciences) were prepared using a strand-specific ribodepletion workflow with the NEBNext rRNA Depletion Kit (Human/Mouse/Rat) and were sequenced on an Illumina NovaSeq X platform according to the provider’s standard protocols.

### RNA-sequencing analysis

All sequencing FASTQ files were trimmed using the Trim_Galore programme (v0.6.2), a wrapper around Cutadapt, with default parameters^67^. The trimmed FASTQ files were then aligned to the human genome (hg38) using the STAR aligner programme (v2.7.9a)^68^ unless otherwise specified. Duplicates were marked and removed using the Picard programme (v3.0.0)^69^. A Gene Transfer Format (GTF) file containing annotations for human reference genes was downloaded from the UCSC Genome Browser as gencode.v21.annotation_GRCh38.gtf.

### Differential gene expression analysis

This GTF file for gene annotation was used with the featureCounts command of the subread tools (v1.6.4)^70^ to quantify the number of reads assigned to each gene. Genes with read counts less than 10 across all samples are removed from downstream analysis. edgeR (v3.40.2)^71^ was used to identify differentially expressed genes (DEG) in pairwise comparisons, DEGs are called genes with an absolute fold change greater than 2 and FDR less than 0.05.

The top 200 most significantly upregulated genes were submitted to the STRING online website (https://string-db.org/; v11.5) to get enriched terms from Gene Ontology and Reactome Pathways. In addition, Gene Set Enrichment Analysis (GSEA) was performed within GSEA software; v4.3.2) to obtain significantly enriched gene set signatures. The top 10 most enriched GO terms and their associated genes were visualised as networks generated using Cytoscape (v3.10.0)^72^.

Single Sample Gene Set Enrichment Analysis (ssGSEA) were performed using the GSVA Bioconductor package (v1.46.0)^73^ to obtain the enrichment score for the 38 core interferon-stimulated genes defined by Liu and colleagues^37^.

### Differential repeat elements expression analysis

Read counts of REs were quantified by applying the SQulRE pipeline^74^. Then the read counts matrix was analysed with the edgeR package to search for differential expressed REs. For each sample, its library size was determined as the total reads mapped to the human genome. REs with read counts less than 10 across all samples are removed from downstream analysis. Significant differently expressed REs are called as absolute fold change greater than 2 and FDR less than 0.05. The genomic distribution of differentially expressed REs was analysed with the GenomicDistributions (v3.17) bioconductor package^75^.

### Western blot

Cells were lysed in buffer containing 0.1% SDS, 400 mM NaCl, 1 mM EDTA, 50 mM Tris-HCl, 1% Triton X-100 and Halt protease inhibitor cocktail (Thermo Fisher Scientific). Whole-cell lysates were clarified, and protein concentrations were quantified using a BCA assay (Promega). Equal amounts of protein (40-50 µg) were denatured at 95 °C for 5 min and were loaded and resolved on 4–20% Mini-PROTEAN TGX gels. Proteins were then transferred to Trans-Blot Turbo Midi nitrocellulose membranes (Bio-Rad). Membranes were blocked in 5% BSA and incubatedwith primary antibodies against METTL3 (D2I6O, #96391S, Cell Signalling Technology; 1:1000 dilution), PKR (EPR19374, ab184257, Abcam; 1:2000 dilution), RNase L (E-9, sc-74405, Santa Cruz; 1:1000 dilution), MAVS (ab89825, Abcam; 1:1000 dilution), α-tubulin (T9026, Sigma-Aldrich; 1:3000 dilution) or vinculin (#700062, Invitrogen; 1:3000 dilution). Following incubation with primary antibodies, membranes were incubated with HRP-conjugated secondary antibodies: anti-rabbit IgG (#7074S, Cell Signalling Technology; 1:5000 dilution) or anti-mouse IgG (#7076S, Cell Signalling Technology; 1:5000 dilution). Blots were developed using Clarity or ClarityMax Western ECL substrate (Bio-Rad) according to the manufacturer’s instructions.

### MDA5-protection assay

To identify the primary MDA5 ligand in colorectal cancer cells that were either wild-type (transduced with control pLKO1 plasmid) or *METTL3* Knock-downed (^KD^), we performed an RNase A/MDA5 protection assay followed by RNA sequencing as previously described^13,45^. Briefly, cytosolic RNA was isolated from HT29 colorectal cancer cell lines using the QIAGEN RNeasy Mini Kit. Purified cytosolic RNA was pre-incubated with recombinant MDA5-Δ2CARD protein (150 nM; kindly provided by Dr. Kazuki Kato) at room temperature for 10 minutes in buffer containing 20 mM HEPES (pH 7.5), 50 mM NaCl, 2 mM MgCl₂, and 2 mM DTT. RNase A was then added to a final concentration of 2 ng/µL, and samples were incubated for an additional 5 min at room temperature. Digestion was terminated by the addition of TRIzol reagent, and RNA was purified using the Zymo Direct-zol RNA Miniprep Kit according to the manufacturer’s instructions. To eliminate small RNA fragments ( < 100 bp) generated during RNase A treatment, samples were further purified using the QIAGEN RNeasy Mini Kit. The resulting RNA was subsequently used for library preparation.

### Library preparation and sequencing of MDA5-protected RNA

cDNA libraries were generated by the Weatherall Institute of Molecular Medicine (WIMM) Single Cell Genomics Facility using 100 ng of input RNA and the KAPA RNA HyperPrep with RiboErase Library Preparation Kit (Roche), following the manufacturer’s guidelines with minor modifications. For RNA fragmentation, total cytosolic RNA samples were incubated at 94 °C for 8 min, whereas MDA5-protected RNA samples were fragmented at 65 °C for 1 min. Adaptor ligation was performed using 1.5 µM KAPA Unique Dual-Indexed Adaptors. Library amplification was carried out on a C1000 Touch Thermal Cycler (Bio-Rad). Initial denaturation was performed at 98 °C for 45 s, followed by 11 PCR cycles for total cytosolic RNA libraries or 16 cycles for RNaseA, digested MDA5-protected RNA libraries (98 °C for 15 s, 60 °C for 30 s, and 72 °C for 30 s). A final extension step was conducted at 72 °C for 1 min. Amplified libraries were purified using a 1 × KAPA Pure Bead cleanup and eluted in 20 µL of 10 mM Tris-HCl (pH 8.0). Library quality and fragment size distribution were assessed using the Agilent D5000 ScreenTape assay on the 2200 TapeStation system. Libraries derived from total cytosolic RNA and MDA5-protected RNA were pooled and sequenced at 6 pM using paired-end 150 bp reads on an Illumina NovaSeq X Plus platform (Novogene).

### MDA5 protection assay analysis

A Gene Transfer Format (GTF) file containing whole-genome repeat element loci, annotated by RepeatMasker, was downloaded from the UCSC Genome Browser table. This GTF file was used with the featureCounts command to quantify the reads count for each repeat element locus from the bam files. Reads that were not assigned to repeat loci were considered non-repeat reads. Read counts were then aggregated for each repeat class, and the percentage of reads for each class was calculated as the ratio of the repeat class counts to the total reads in the BAM file.

### Identification of MDA5-protected regions

MDA5-protected regions were identified as sequencing signal peaks from RNaseA-treated samples against total cytoplasmic RNA samples. For each sample, total BAM files were split into forward strand aligned and reverse strand aligned bam files, and then peaks were called from strand-specific BAM files using MACS2 (v2.2.6)^76^ with the following parameters: -q 0.01 –keep-dup all –nomodel. Peaks identified from the forward and reverse strands were combined for downstream analysis. Heatmaps visualising signals in the peak regions were generated using deepTools (v3.5.3)^77^. When comparing peaks identified from two groups, those sharing at least 20% of their regions were classified as common peaks, while the rest were designated as group-specific peaks.

### Identification of MDA5-protected inverted repeats

We first located all repeat element loci that overlapped with at least 30% of the MDA5-protected regions identified previously. Next, to identify nearby repeat element pairs capable of complementing each other to form double-stranded structures, we focused on pairs separated by less than 3 kb. Specifically, the distance between the end of the upstream repeat and the start of the downstream repeat needed to fall within this cutoff. Within the defined distance threshold, each repeat element could form multiple potential pairs. To evaluate whether these pairs could form reverse complementary double-stranded structures, we performed local alignment in a reverse complement orientation using the Smith–Waterman algorithm implemented in the exonerate tool (v2.2.0)^78^ with default parameters. Two penalty scores were applied: a gap-opening penalty of 12 and a gap- extension penalty of 4, which are the programme’s default values. Pairs with an alignment score below 500 were discarded. Finally, repeat pairs separated by less than 3 kb and with a reverse complement score greater than 500 were classified as inverted repeats (IRs). The number of IRs identified in each group was counted, and their intersections were compared. The genomic distribution of MDA5 protected regions and inverted repeats were analysed with the GenomicDistributions Bioconductor package^75^.

### Colony forming assay and crystal violet

Cell numbers were counted using a TC20 automated cell counter (Bio-Rad). For crystal violet assays, cells were seeded in 6-well plates at densities of 1,000–1,500 cells per well for colony formation assays and 1.0 × 10⁵–1.5 × 10⁵ cells per well for proliferation assays. Cells were cultured until control wells approached confluence. At the endpoint, wells were rinsed twice with ice-cold PBS, fixed in ice-cold methanol for 10 minutes on ice, and subsequently stained with 0.5% crystal violet prepared in 25% methanol for 10 min at room temperature. Excess stain was removed by washing the plates thoroughly (at least six times) with distilled water. Plates were air-dried, and images were captured using a digital camera.

### Generation of double-knockout (DKO) cell lines

*METTL3* single-knockout (KO) HT29 cells were generated using a custom-designed sgRNA (Invitrogen, #A35534) targeting human *METTL3* (sequence: CUUAGAUCUACGGAAUCCAG). Cells were seeded at 5 × 10⁵ cells per well in 6-well plates and transfected the following day with Cas9 protein (Invitrogen, #A36498) and sgRNA using Cas9 PLUS Reagent and Lipofectamine CRISPRMAX (Invitrogen, CMAX00003) in Opti-MEM, according to the manufacturer’s instructions. After 48 h, single cells were seeded into 96-well plates to derive monoclonal clones. Knockout efficiency was validated by immunoblotting.

For the generation of double-knockout (DKO) HT29 cells, a validated *METTL3* KO clone was further edited using IDT predesigned sgRNAs (sequences provided in Supplementary Data 6). Ribonucleoprotein (RNP) complexes were assembled by combining Alt-R™ CRISPR–Cas9 crRNA with Alt-R™ CRISPR–Cas9 tracrRNA (IDT, #1072533) and Alt-R™ S.p. HiFi Cas9 Nuclease V3 (#1081061), according to the manufacturer’s instructions. RNP complexes were delivered by electroporation using pulse code EH115 with the Lonza P3 Primary Cell 4D-Nucleofector™ X Kit S (Lonza, #V4XP-3032). After 48 h, single cells were sorted into 96-well plates to obtain monoclonal DKO clones. Successful gene disruption was confirmed by immunoblotting.

PKR (*EIF2AK2*), *MAVS* and *RNaseL* knockout HT29 cells were generated using IDT predesigned sgRNAs and the same RNP electroporation strategy described above.

### In vitro limiting dilution Assay (LDA)

For in vitro limiting dilution assays (LDAs), HT29 and HCT116 control (CTRL) or *METTL3* knockdown (*METTL3*^KD^) cells were treated with decitabine (300 nM) or vehicle control (PBS) on day 0 and maintained in culture for 5 days. On day 5, cells were harvested, washed to remove residual drug, dissociated into single-cell suspensions, and plated in 96-well plates at serial dilutions (1, 10, 100, or 1000 cells per well). For each cell density, a minimum of 18 wells was seeded, and for lower cell numbers, at least 72 wells were plated. After 4 weeks of culture, wells were scored for colony formation based on the presence or absence of colonies. Cancer-initiating cell (CIC) frequency was calculated using extreme limiting dilution analysis (ELDA) software (http://bioinf.wehi.edu.au/software/elda/index.html) provided by the Walter and Eliza Hall Institute^79^.

### In vivo experiment

All mouse procedures were carried out in accordance with UK Animals (Scientific Procedures) Act 1986 and University of Oxford Animal Welfare and Ethical Review Body approval under Project license (PPL) number PP7109477. Mice were maintained under specific pathogen-free conditions at the Centre for Human Genetics Animal Facility at an ambient temperature of 19–23 °C, with 45–65% relative humidity and a 12-hour light/dark cycle. Six- to eight-week-old male and female NOD.Cg-Prkdc^scid^ Il2rg^tm1Wjl^/SzJ (NSG) mice (Charles River Laboratories, strain 614) were injected subcutaneously with 5 × 10^5^ HT29 cells or 2.5 × 10^5^ HCT116 colorectal cancer cells. Decitabine (Sigma-Aldrich) was dissolved in phosphate-buffered saline (PBS) and administered once tumours in the control cohort reached approximately 100 mm³. Mice received decitabine at 0.5 mg/kg via intraperitoneal injection once daily for four consecutive days, followed by a 3-day break and a further four consecutive days of treatment. Control animals received intraperitoneal injections of PBS according to the same schedule. Tumour growth was monitored until tumours in the vehicle-treated group reached the maximum permitted size, with no tumours exceeding 1500 mm³.

### IFNβ-promoter reporter assay

IFNβ promoter reporter HEK293 cells (p125-HEK), including wild-type (WT) and *MAVS* knockout (*MAVS*^KO^) cells, were kindly provided by Prof. Jan Rehwinkel’s laboratory and were originally established as previously described^80^. For luciferase assays, cells were seeded into 96-well plates (Corning) at a density of 1 × 10^4^–2 × 10^4^ cells per well. The following day, cells were transfected with 100 ng of total RNA isolated from HT29 or HCT116 cells using 0.2 μL Lipofectamine 2000 per well. IFNβ promoter activity was measured 48 hours post-transfection using the One-Glo luciferase assay system (Promega).

### MeRIP-seq (Methylation RNA immunoprecipitation- sequencing)

MeRIP was performed according to the manufacturer’s instructions (Magna MeRIP Kit, Merck, Cat# 17-10499). Briefly, 300 µg of total RNA was extracted using the RNeasy Mini Kit and fragmented to approximately 100 nucleotides by heating at 94 °C for 4 min. Immunoprecipitation was performed using Magna ChIP Protein A/G Magnetic Beads (Cat# CS203152) and an anti-m^6^A antibody (Cat# MABE1006). The RNA–antibody–bead mixture was incubated for 1 h at room temperature to facilitate binding. After immunoprecipitation, RNA was eluted using the provided elution buffer and purified with the RNeasy Mini Kit. For library preparation, 100 ng of input and immunoprecipitated (IP) RNA were used. Libraries were generated using the NEBNext® Ultra™ II Directional RNA Library Prep Kit for Illumina® (Cat. #E7760L) in combination with NEBNext® Multiplex Oligos for Illumina® (Index Primer Set 1; Cat. #E7335S/L). The resulting cDNA libraries were sequenced using a paired-end 100-bp run configuration on the NextSeq 2000 platform.

### MeRIP analysis

In the summary of the trimming process for FASTQ files from MeRIP samples, we observed that in several samples, the first read from our paired-end sequencing exhibited lower sequencing quality. To minimise bias, only the second sequencing reads were used for downstream analysis. The second read FASTQ files were first aligned to the human ribosomal RNA sequence using the HISAT2 aligner programme (v2.2.1)^81^, reads that did not align to the ribosomal RNA were then re-aligned to the human hg38 genome. Duplicates were marked and subsequently removed using the Picard programme. m^6^A peaks were identified from BAM files of IP samples compared to Input samples using MACS2 with the parameters: -p 0.000001 –nomodel –keep-dup all. m^6^A enriched motifs were identified from the top 2000 most significant m^6^A peaks using the find Motifs.pl script from the HOMER software (v4.9.1)^82^.

The IP and Input signals for each gene were quantified using featureCounts and normalised to their respective sequencing library depths, the m^6^A level of each gene was calculated as the ratio of the IP signal to the Input signal for that gene. edgeR was used to identify differentially m6A methylated genes (DMGs) with consideration of both IP signals and Input signals. DMGs were identified as genes with an absolute fold change in m^6^A methylation levels greater than 2 and a false discovery rate (FDR) of less than 0.05. The IP and Input signals for each repeat element locus were quantified using the SQulRE pipeline and normalised to their respective sequencing library depths. A similar method used to identify DMGs was applied to detect differentially m6A methylated repeat element loci. The average m^6^A methylation levels of induced or baseline MDA5-protected IRs and their flanking 50 kb regions were calculated using the computeMatrix function from deepTools.

### dsRNA Detection by J2 Dot Blot

Total RNA was extracted from HT29, HCT116, COLO201, NCI-H716, POP92, and CSC73 cell lines using the RNeasy Mini Kit. Equal volumes and amounts of RNA (3 μL; 500 ng–1 μg) were spotted onto a Hybond N^+^ membrane (GE Healthcare, Cat# RPN119B), air-dried, and UV crosslinked twice using a Stratalinker (Stratagene). The membrane was blocked with 5% milk in PBS-T (0.1% Tween-20) for 1 h at room temperature and subsequently incubated overnight at 4 °C with anti-dsRNA monoclonal antibody J2 (mouse IgG2a, κ chain; Jena Bioscience, Cat# RNT-SCI-10010500-JEN) at a 1:500 dilution. The next day, membranes were washed three times with PBS-T and incubated with HRP-conjugated anti-mouse IgG secondary antibody (Cell Signalling Technology, Cat# 7076S; 1:5000 dilution in 5% milk) for 1 h at room temperature. After three additional washes with PBS-T, signals were detected using an enhanced chemiluminescence (ECL) reagent and imaged on a Bio-Rad ChemiDoc system. Membranes were then stained with methylene blue to verify equal RNA loading and imaged using the colorimetric detection setting of the ChemiDoc system.

### m^6^A Detection by RNA Dot Blot

Dot blot analysis of m^6^A levels was performed as previously described^83^. Total RNA was extracted from HT29, HCT116, COLO201, and NCI-H716 cell lines, as well as from POP92 and CSC73 PDX samples, using the RNeasy Mini Kit. RNA samples were denatured at 95 °C for 3 min in a heat block to disrupt secondary structures and immediately chilled on ice to prevent re-annealing. Equal volumes and amounts of RNA (3 μL; 500 ng–1 μg) were spotted onto a Hybond N^+^ membrane (GE Healthcare, Cat# RPN119B), air-dried, and UV crosslinked twice using a Stratalinker (Stratagene). The membrane was blocked with 5% milk in PBS-T (0.1% Tween-20) for 1 h at room temperature and incubated overnight at 4 °C with anti-m^6^A antibody (Synaptic Systems [SYSY], Cat# 202 003; 1:250 dilution). The following day, membranes were washed three times with PBS-T and incubated with HRP-conjugated anti-rabbit IgG secondary antibody (Cell Signalling Technology, Cat# 7074S; 1:5000 dilution in 5% milk) for 1 h at room temperature. After three additional washes with PBS-T, signals were detected using Clarity Max Western ECL Substrate (Bio-Rad, Cat# 1705062) or Clarity Western ECL Substrate (Bio-Rad, Cat# 1705061) and imaged on a Bio-Rad ChemiDoc system. Membranes were subsequently stained with methylene blue to verify equal RNA loading and imaged using the colorimetric detection mode of the ChemiDoc system.

### m⁶A and dsRNA immunoprecipitation assays

m⁶A RNA immunoprecipitation (MeRIP) was performed using the Magna MeRIP kit (Merck), according to the manufacturer’s instructions, followed by dot-blot analysis. Briefly, total RNA was isolated from five 15 cm dishes of HT29 and/or HCT116 cells (CTRL or *METTL3*^KD^) using the Qiagen RNeasy Midi Kit and fragmented prior to immunoprecipitation. Immunoprecipitation was performed using an anti-m⁶A antibody bound to Protein A/G magnetic beads. RNA recovered from input and immunoprecipitated (IP) fractions was spotted onto a Hybond N^+^ membrane (GE Healthcare, Cat# RPN119B and UV crosslinked using a Stratalinker. Membranes were blocked and probed with anti-dsRNA antibody J2 to detect dsRNA in m⁶A input and IP samples. For dsRNA immunoprecipitation (dsRNA-IP), IP was performed using the anti-dsRNA J2 antibody, and recovered RNA was analysed by dot blot using an anti-m⁶A antibody. Blocking, incubation with the appropriate HRP-conjugated secondary antibodies, washing steps, and signal detection using ECL substrate (Clarity or Clarity Max, Bio-Rad) were performed as described above for dot blot analysis.

### Cell viability analysis by CellTiter-Glo

HT29 and HCT116 colorectal cancer (CRC) cells were seeded in opaque 96-well plates at a density of 2,500–5,000 cells per well in 50 μL of culture medium. After 24 h, cells were treated with increasing concentrations of Decitabine (0.029, 0.05, 0.11, 0.23, 0.46, 0.93, 1.87, 3.75, 7.5, 15, and 30 μM). Mock-treated cells and medium-only wells were included as controls. Following 5 days of treatment, plates were equilibrated to room temperature for 30 min before the addition of CellTiter-Glo® Luminescent Cell Viability Assay reagent (Promega, Cat# G7572) at an equal volume (100 μL per well). Plates were shaken on an orbital shaker for 2 min and incubated at room temperature for an additional 10 min to stabilise the luminescent signal. Luminescence was measured using a FLUOSTAR plate reader. Signals were normalised to control wells, and dose–response curves were generated to calculate EC50 values using nonlinear regression analysis.

### Immunofluorescence staining and microscopy

Glass coverslips were placed in a 24-well plate, briefly treated with 100% ethanol (1 mL per well) for 5 min at room temperature, and rinsed with phosphate-buffered saline (PBS). CRC cells were then plated onto the coverslips at a concentration of 1 × 10⁵ cells/mL and cultured overnight at 37 °C to permit adherence. The next day, cells were gently washed with PBS and fixed in pre-chilled methanol at − 20 °C for 15 min. Fixed cells were washed three times with PBS and incubated in blocking solution (1% bovine serum albumin in PBS) for 1 h at 37 °C to reduce nonspecific binding. For the detection of dsRNA, cells were incubated overnight at 4 °C with J2 primary antibody diluted 1:500 in blocking buffer. Following primary antibody incubation, coverslips were washed three times in PBS (10 minutes each) and then exposed to Alexa Fluor 647–conjugated anti-mouse IgG (H + L) F(ab′)₂ secondary antibody (Cell Signalling Technology, Cat# 4410S) at a 1:1000 dilution in 1% BSA for 1 h at room temperature. Samples were protected from light during and after secondary antibody incubation. Excess antibody was removed by three additional 10-minute washes in PBS. For nuclear staining, cells were incubated with Hoechst 33342 (Invitrogen™, Thermo Fisher Scientific, Cat# H1399; 1:2000 dilution in PBS) for 5 min at room temperature, followed by PBS washes. Coverslips were mounted onto glass slides using ProLong Gold Antifade Mountant (Thermo Fisher Scientific, Cat# P36930), with the cell-coated surface facing the mounting medium. Confocal images were acquired using a Zeiss LSM980 microscope equipped with a 40 × oil-immersion objective. Fluorescence intensity was quantified using ImageJ software. Corrected total cell fluorescence (CTCF) was calculated as:

CTCF = Integrated Density − (Area of selected cell × Mean background fluorescence). The J2 antibody specifically recognises double-stranded RNA species of at least 40 base pairs in length.

### CUT&RUN for H3K4me3 Profiling

CUT&RUN experiments were carried out in CTRL and *METTL3*^KD^ colorectal cancer (CRC) cells using the CUT&RUN Assay Kit (Cell Signalling Technology, Cat# 86652) according to the manufacturer’s protocol. For each condition, 1 × 10⁶ cells were immobilised on Concanavalin A–coated magnetic beads and incubated overnight at 4 °C with anti-H3K4me3 antibody (Cell Signalling Technology, Cat# 9751; 1:50 dilution). Following antibody binding, bead-bound cells were washed in digitonin-containing buffer prior to the addition of Protein A–micrococcal nuclease (pA-MNase), which was incubated for 1 h at 4 °C. Chromatin digestion was initiated by adding 3 µL of 100 mM CaCl₂ and incubating the samples on ice for 30 min. The reaction was quenched by the addition of a stop buffer containing EDTA and EGTA. Released chromatin fragments were collected in the supernatant after centrifugation, and DNA was purified using the QIAGEN MinElute Purification Kit. Purified DNA was then used for next-generation sequencing library preparation with the NEBNext Ultra II DNA Library Prep Kit according to the manufacturer’s instructions. Libraries were sequenced on an Illumina NextSeq 2000 platform to generate paired-end reads.

### CUT&RUN analysis

The trimmed fastq files were aligned to the human hg38 genome using the Bowtie2 aligner programme (v.2.3.4.2)^84^ with parameters set as: -I 10 -X 700 –local –very-sensitive- local –no-mixed –no-discordant –no-unal. Duplicates were marked and then removed using picard programme. To perform spike-in normalisation, the same fastq files were aligned to the sacCer3 yeast genome using Bowtie2 with the parameters setting as: -I 10 -X 700 –local –very- sensitive-local –no-mixed –no-discordant –no-unal –no-overlap –no-dovetail. The spike-in normalisation factor was calculated as the ratio between 20,000 and the total reads aligned to the yeast genome. Genome coverage of each sample was generated from the alignment files using the genomecov command in the bedtools suite (v.2.28.0)^85^ with the scale parameter setting as the spike-in normalisation factor to normalise the library. Peaks were called from Human genome aligned BAM files using MACS2 with the following parameters: -q 0.01 -B –SPMR –keep-dup all. IgG samples served as controls. Heatmaps visualising signals in peak regions were produced using deeptools.

H3K4me3 occupancy within 3000 nucleotides upstream and downstream of the transcription start site (TSS) was quantified using featureCounts with a custom- generated BED file. edgeR was used to identify differentially occupied genes (DOGs) for H3K4me3 in pairwise comparisons. DOGs were defined as genes with an absolute fold change greater than 2 and a false discovery rate (FDR) below 0.05. The average occupancy of H3K4me3 in induced or baseline MDA5-protected IRs and their flanking 50 kb regions was calculated separately using the computeMatrix function from deepTools.

### RNA stability assay

HT29 CTRL and *METTL3*^KD^ cells were seeded at a density of 5 × 10⁴ cells/mL in 100 mm culture dishes. After 24 h, one dish from each condition was collected to serve as the 0-hour time point. The remaining dishes were treated with either DMSO (vehicle control) or actinomycin D (5 µg/mL; Sigma-Aldrich, Cat# A9415-2MG) to inhibit transcription, and cells were collected every 2 h following treatment. Total RNA was extracted using the RNeasy Mini Kit (Qiagen) with on-column DNase I digestion according to the manufacturer’s instructions. cDNA was synthesised from purified RNA, and transcript levels were quantified by qPCR. To determine the half-life of RNA transcripts, the expression levels of the examined RNAs were first normalised to the corresponding *GAPDH* expression at each time point. The relative amount of RNA remaining was then calculated by dividing the normalised RNA level at each time point by the level at the 0 h time point. The degradation curves for each RNA were generated by fitting the equation ${{\rm{y}}}={e}^{-k({decay})x}$ to the relative RNA-remaining values across the measured time points. The half-life was subsequently calculated using the formula ${t}_{1/2}=\frac{{\mathrm{ln}}(2)}{k({decay})}$.

### RNA secondary structure prediction

To predict the secondary structures of IR-Alu transcripts, the raw RNA sequences, including the upstream and downstream Alu elements as well as the connecting regions, were submitted to the RNAfold WebServer (http://rna.tbi.univie.ac.at/cgi-bin/RNAWebSuite/RNAfold.cgi)^86^ using the default settings, except that energy corrections for modified bases were enabled. The predicted minimum free energy (MFE) secondary structures were then downloaded, and the corresponding MFE values were extracted.

To examine the effect of m⁶A modification on RNA secondary structure, all adenosines within the m⁶A consensus motif (DRACH) in the raw IR-Alu RNA sequences were substituted with 6 and submitted to the RNAfold WebServer using the same settings. The predicted MFE structures were downloaded, and the MFE values were similarly extracted.

### Public data

Gene expression data for samples from the TCGA dataset were retrieved from the UCSC Toil RNA-seq Recompute Compendium^87^. The *METTL3* knockout gene effect in cancer cell lines, quantified as dependency probability, was obtained from DepMap (v24Q2) (https://depmap.org/portal)^36^. Microsatellite instability status of colorectal cancers (CRCs) was also acquired from DepMap, while the CpG Island Methylator Phenotype status of CRCs was obtained from ref. ^88^.

Consensus molecular subtyping (CMS) of CRCs was conducted using the SSP method in the CMSclassifier R package (v1.0.0)^41^. The input gene expression data, downloaded from DepMap, allowed the classification of samples into CMS1, CMS2, CMS3, CMS4, or non-consensus groups. Notably, no CRC samples were assigned to the CMS4 subtype.

The association between CRC genetic mutations and the effects of the *METTL3* gene was visualised using the oncoplot function from the maftools R package (v2.18.0)^89^. The input data for this analysis, including somatic mutation profiles and *METTL3* gene effect scores for each CRC, were obtained from DepMap. A summary of colorectal cancer cell lines analysed in this study, including their METTL3 dependency probability, CIMP status, MSS/MSI status, and KRAS mutation status, is provided in Supplementary Data 7.

### Statistical analysis & illustration

The number of samples used in each experiment is provided in the figure legends. GraphPad Prism (v11.0.0) was used for statistical analyses and data visualisation, and schematic illustrations were created with BioRender.com. Statistical analyses of sequencing and publicly available datasets were performed in R (v4.3.2). Significance was assessed using two-sided Wilcoxon rank-sum tests, two-sided Student’s t-tests, or other methods as specified in the relevant sections. The Benjamini–Hochberg method was applied to adjust for multiple hypothesis testing where appropriate.

### Reporting summary

Further information on research design is available in the Nature Portfolio Reporting Summary linked to this article.

## Supplementary information

Supplementary Information

Description of Additional Supplementary Files

Supplementary Data 1-7

Reporting Summary

Transparent Peer Review file

## Source data

Source Data

## Data Availability

All raw and processed sequencing data generated in this study have been deposited in the Gene Expression Omnibus (GEO) database under the accession number GSE289982. Source data are provided in this paper.

## Code availability

All scripts used for the analyses included in this study are publicly available in the GitHub repository at https://github.com/yiluyucheng/m6A_dsRNA, and are also cited as reference^90^.
