# Supplementary material for: m6A modification suppresses innate anti-tumour immunity in colorectal cancer by limiting alu-derived dsRNA accumulation
Source: Nat Commun. 2026 May 14;17:6428. doi: 10.1038/s41467-026-73211-z (PMC13377058; doi:10.1038/s41467-026-73211-z)
Supplement: Supplementary file 1 — Supplementary Information [file 41467_2026_73211_MOESM1_ESM.pdf]

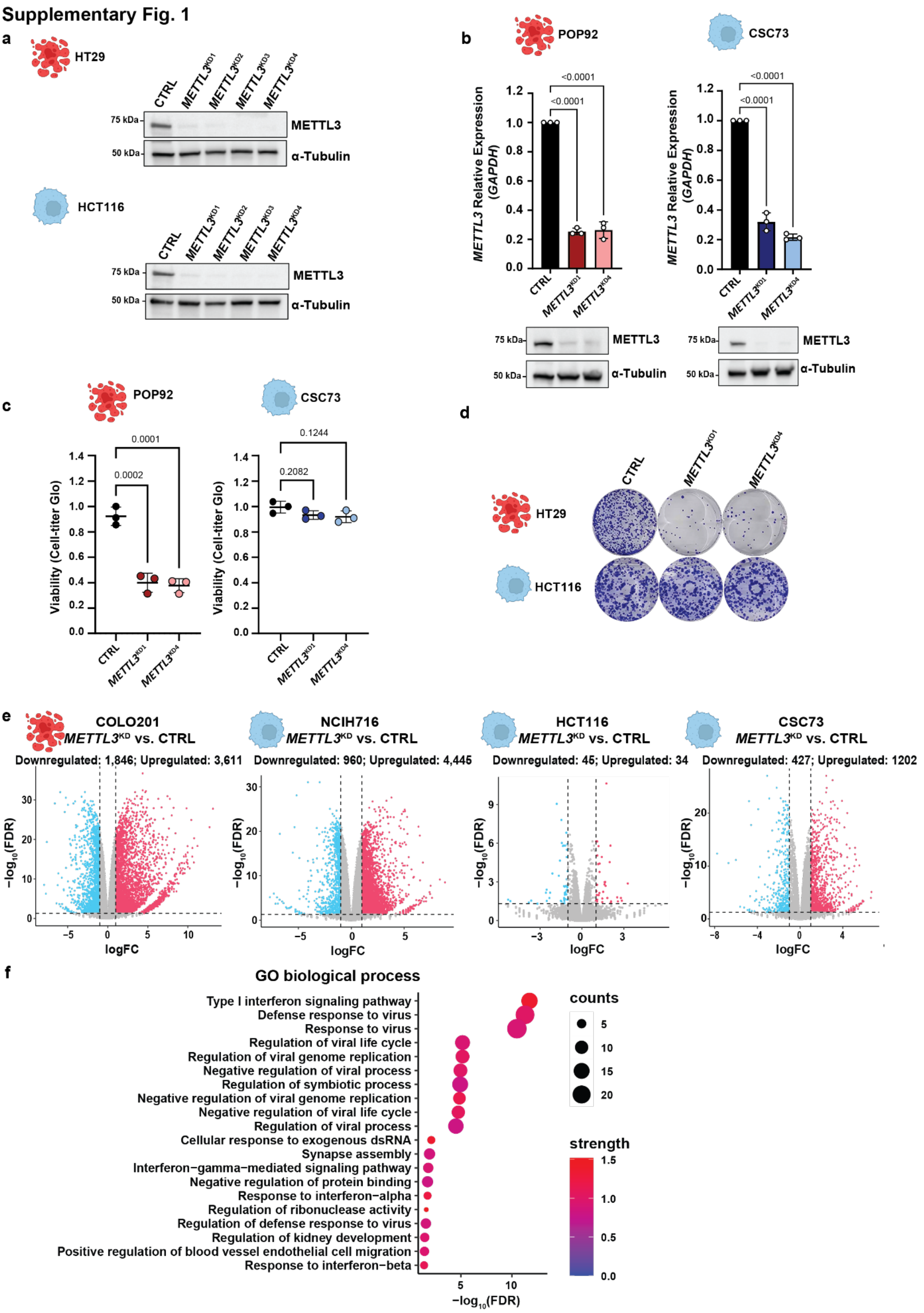
Supplementary Fig. 1
a
HT29
CTRL
METTL3KD1
METTL3KD2
METTL3KD3
METTL3KD4
75 kDa
METTL3
50 kDa
α-Tubulin
HCT116
b
POP92
CSC73
METTL3 Relative Expression (GAPDH)
<0.0001
c
Viability (Cell-titer Glo)
0.0001
0.0002
0.1244
0.2082
d
e
COLO201 METTL3KD vs. CTRL
Downregulated: 1,846; Upregulated: 3,611
NCIH716 METTL3KD vs. CTRL
Downregulated: 960; Upregulated: 4,445
HCT116 METTL3KD vs. CTRL
Downregulated: 45; Upregulated: 34
CSC73 METTL3KD vs. CTRL
Downregulated: 427; Upregulated: 1202
−log10(FDR)
logFC
f
GO biological process
Type I interferon signaling pathway
Defense response to virus
Response to virus
Regulation of viral life cycle
Regulation of viral genome replication
Negative regulation of viral process
Regulation of symbiotic process
Negative regulation of viral genome replication
Negative regulation of viral life cycle
Regulation of viral process
Cellular response to exogenous dsRNA
Synapse assembly
Interferon−gamma−mediated signaling pathway
Negative regulation of protein binding
Response to interferon−alpha
Regulation of ribonuclease activity
Regulation of defense response to virus
Regulation of kidney development
Positive regulation of blood vessel endothelial cell migration
Response to interferon−beta
counts
strength
−log10(FDR)

Supplementary Fig. 1. Selective vulnerability of CRCs to METTL3 depletion.

a. Immunoblot analysis of *METTL3* knockdown (^{KD}) efficiency using four different short hairpin RNAs (shRNAs; $^{KD1–KD4}$) in HT29 (red) and HCT116 (blue) colorectal cancer (CRC) cell lines, with α-tubulin as the loading control. Representative immunoblot from three independent experiments.

b. Quantitative PCR (qPCR; top) and immunoblot (bottom) analyses of *METTL3*^{KD} efficiency using two different short hairpin RNAs (shRNAs; ^{KD1} and ^{KD4}) in CSC73 and POP92 patient-derived xenograft (PDX) colorectal cancer (CRC) cells. qPCR data are presented as mean ± SD (n = 3 independent experiments). Statistical significance was determined by one-way ANOVA with Dunnett's multiple-comparisons correction. A representative immunoblot from three independent experiments is shown for METTL3 protein levels, with α-tubulin as the loading control.

c. Viability of *METTL3* wild-type (CTRL) and *METTL3*^{KD} CSC73 and POP92 PDX CRC cells, as measured by the CellTiter-Glo luminescence assay. Data are presented as mean ± SD (n = 3 independent experiments). Statistical significance was determined by one-way ANOVA with Dunnett's multiple-comparisons correction.

d. Colony-forming ability of HT29 wild-type (CTRL) and *METTL3*^{KD} cells (top) and HCT116 cells (bottom), as assessed by crystal violet staining.

e. Volcano plot showing differential gene expression between *METTL3*^{KD} and CTRL cells in COLO201, NCI-H716, HCT116, and CSC73 models. Red indicates upregulated genes; blue indicates downregulated genes; grey indicates no significant change. Data represent two independent experiments.

f. Gene Ontology (GO) biological process enrichment analysis showing the top 20 significantly enriched terms (false_discovery_rate (FDR) < 0.05) derived from the 200 most upregulated genes in HT29 *METTL3*^{KD} cells compared to CTRL. Dot size corresponds to the number of genes associated with each term, and colour indicates the enrichment strength.

Source data are provided as a Source Data file.

Supplementary Fig. 2

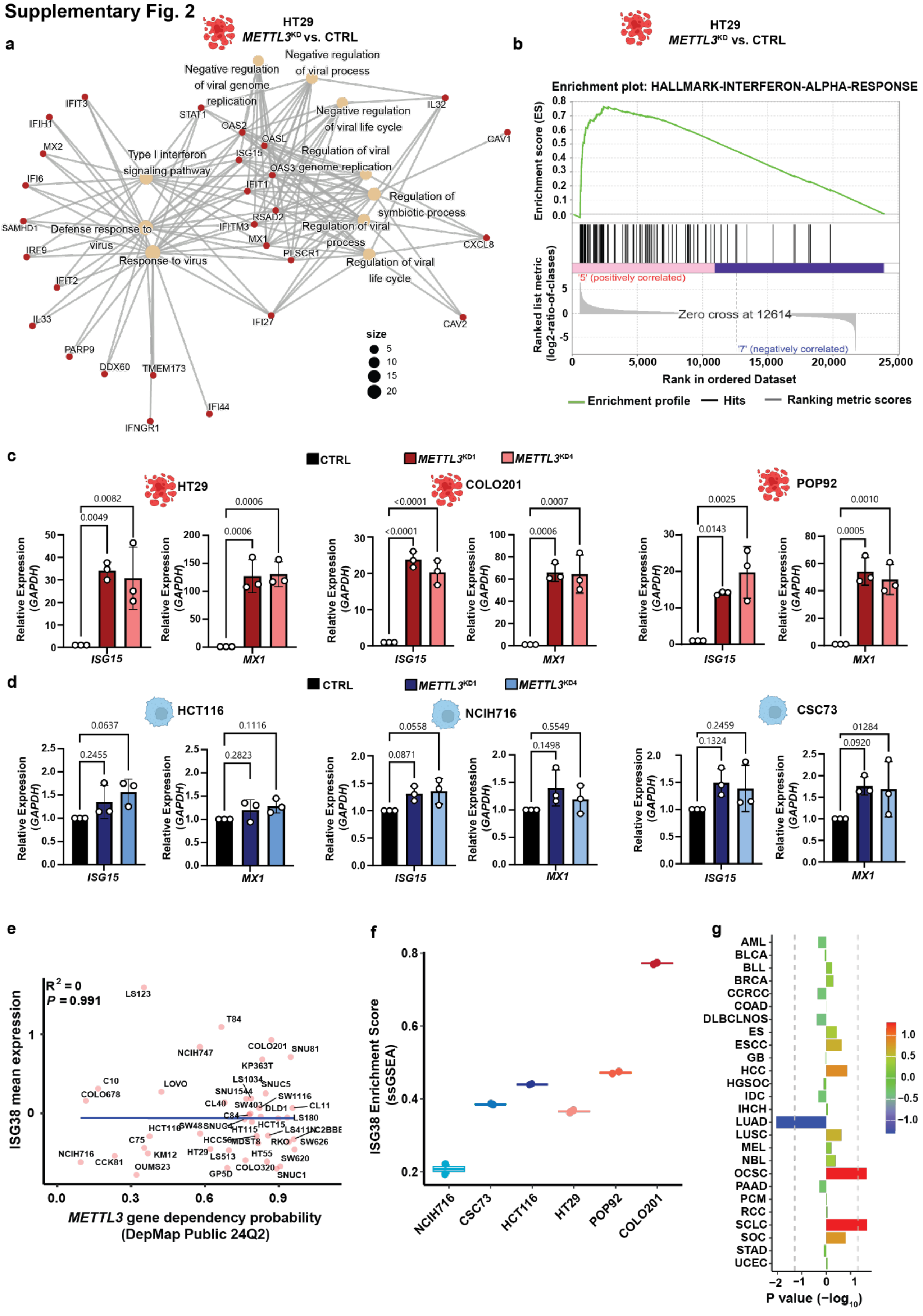

Supplementary Fig. 2. Characterization of CRC sensitivity to METTL3 depletion.

a. Gene–concept network illustrating the top 10 enriched pathways and their associated upregulated genes in HT29 *METTL3*^{KD} cells compared to CTRL.

b. Gene set enrichment analysis (GSEA) showing enrichment of the interferon-α response signature in HT29 *METTL3*^{KD} cells compared to CTRL.

c, d. Quantitative PCR (qPCR) analysis of interferon-stimulated genes (ISGs; *ISG15* and *MX1*) in CTRL and *METTL3*^{KD} HT29, COLO201, and POP92 (**c**) and HCT116, NCI-H617 and CSC73 (**d**) colorectal cancer (CRC) cells. qPCR data are presented as mean ± SD (n = 3 independent experiments). Statistical significance was determined by one-way ANOVA with Dunnett's multiple-comparisons correction.

e. Scatter plot showing the correlation between ISG38 mean expression and *METTL3* gene dependency probability in colorectal cancer cell lines. The blue line represents the linear regression between ISG38 mean expression and *METTL3* gene dependency probability.

f. Basal ISG38 single-sample gene set enrichment analysis (ssGSEA) scores in NCI-H716, CSC73, HCT116, HT29, COLO201, and POP92 colorectal cancer (CRC) cells. Data represent two independent experiments.

g. Bar plot showing the significance of the linear correlation between *METTL3* gene dependency probability (DepMap 24Q2) and ISG38 mean expression across cell lines from 20 cancer types. Bar height and colour correspond to the –log10(*P* value), with negative values indicating a negative association. Horizontal dashed lines denote the significance thresholds at ±log10(0.05).

Source data are provided as a Source Data file.

Supplementary Fig. 3

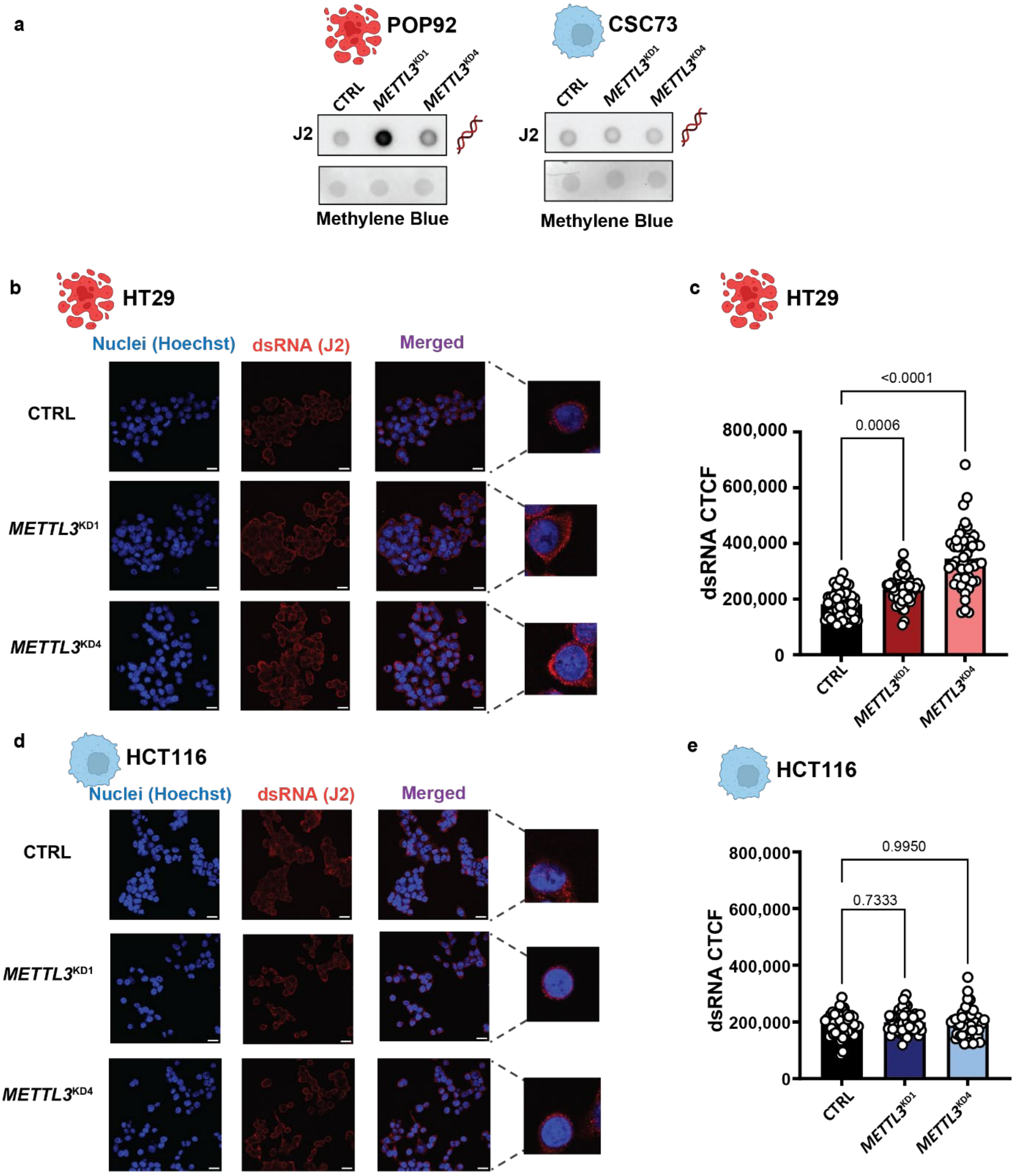

Supplementary Fig. 3. METTL3 loss induces accumulation of dsRNAs in a subset of CRCs.

a. Dot blot analysis of double-stranded RNA (dsRNA), detected using the anti-J2 antibody, in total RNA isolated from the indicated patient-derived xenograft (PDX) colorectal cancer (CRC) samples. Representative dot blots from three independent experiments. The dsRNA icon was Created in BioRender. Mehdipour, P. (2026) https://BioRender.com/nouh2yx.

b. Representative confocal microscopy images from two independent experiments of CTRL and *METTL3*^{KD} HT29 cells. Nuclei were stained with Hoechst 33342 (blue), and dsRNA was detected using the J2 antibody (red). Scale bars, 20 μm.

c. dsRNA quantification by ImageJ in HT29 CTRL and *METTL3*^{KD} cells. Data are presented as mean ± SD (n = 50 randomly sampled regions from two independent experiments). Statistical significance was determined by ordinary one-way ANOVA with Dunnett's multiple-comparisons correction.

d. Representative confocal microscopy images from two independent experiments of CTRL and *METTL3*^{KD} HCT116 cells. Nuclei were stained with Hoechst 33342 (blue), and dsRNA was detected using the J2 antibody (red). Scale bars, 20 μm.

e. dsRNA quantification by ImageJ in HCT116 CTRL and *METTL3*^{KD} cells. Data are presented as mean ± SD (n = 50 randomly sampled regions from two independent experiments). Statistical significance was determined by ordinary one-way ANOVA with Dunnett's multiple-comparisons correction.

Source data are provided as a Source Data file.

Supplementary Fig. 4

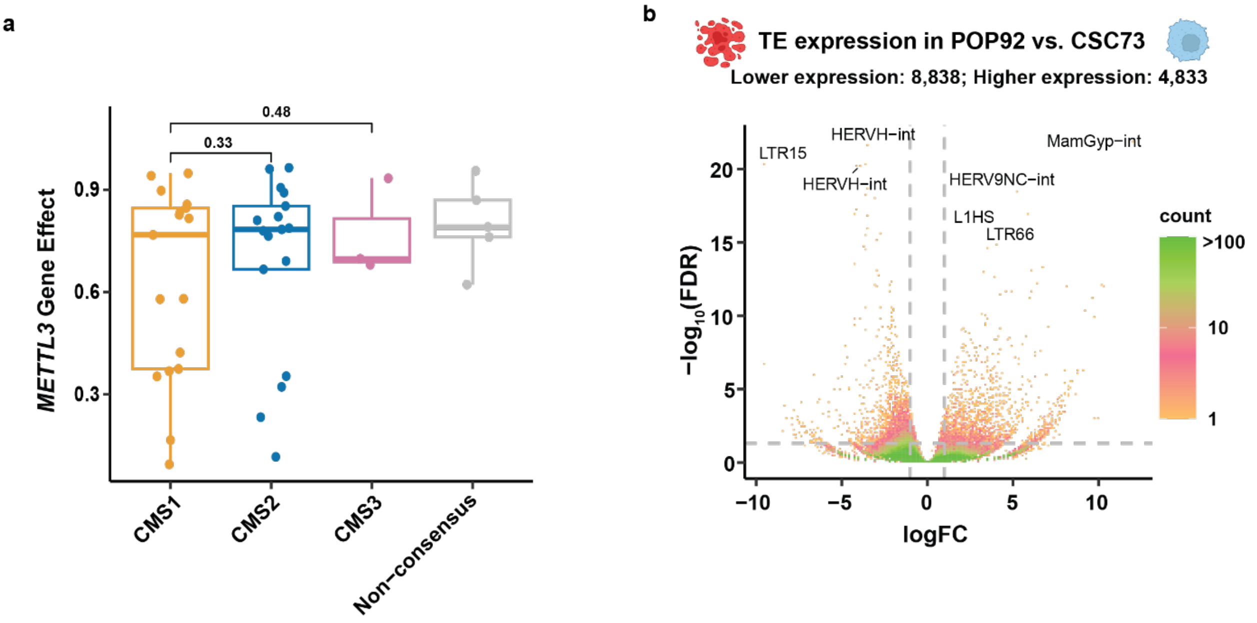

Supplementary Fig. 4. *METTL3* dependency is independent of colorectal cancer consensus molecular subtypes.

a. Box plot showing *METTL3* dependency in colon adenocarcinoma (COAD) cell lines stratified by consensus molecular subtypes (CMS). Statistical significance was determined by a two-sided Wilcoxon rank-sum test. Boxes represent the median (centre line) and the interquartile range (IQR; 25th-75th percentiles). Whiskers extend to 1.5 × IQR from the quartiles; observations beyond this range are not shown.

b. Volcano plots showing differential transposable element (TE) expression based on total RNA-sequencing comparing POP92 and CSC73 patient-derived xenograft (PDX) colorectal cancer (CRC) cells. Colour indicates TE density, and dotted lines denote the significance thresholds (false discovery rate (FDR) < 0.05 and |log2FC| ≥ 1) used to define differentially expressed TEs. Data represent two independent experiments.

Supplementary Fig. 5

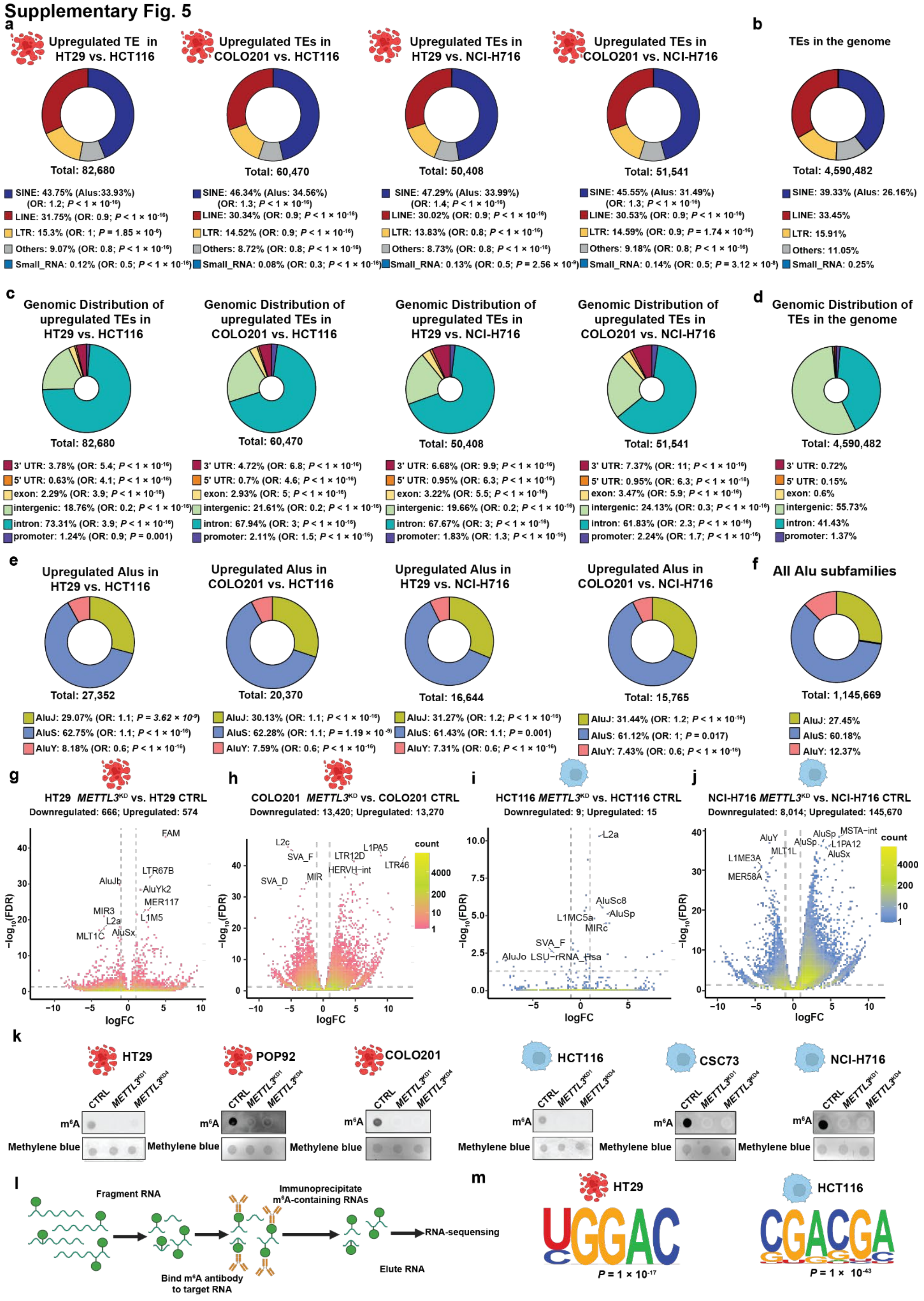

Supplementary Fig. 5. Differential response of CRCs to *METTL3* targeting is not associated with compensation of global m^{6}A levels.

a-d. Class representation (**a**) and genomic representation (**c**) of transposable elements (TEs) with higher expression in METTL3-sensitive compared to METTL3-insensitive CRC cells. The odds ratio (OR) indicates enrichment or depletion for each category relative to the distribution of TEs annotated in the human genome (**b,d**). Statistical significance was determined by two-sided Fisher's exact test.

e,f. Distribution of Alu subfamilies upregulated in METTL3-sensitive compared to METTL3-insensitive CRC cells and relative to all Alu elements annotated in the human genome (n = 1,145,669 Alu elements). The odds ratio (OR) indicates enrichment or depletion of each Alu subfamily (AluS, AluJ, and AluY) in the indicated CRC cells compared to the genome-wide Alu subfamily distribution. Statistical significance was determined by two-sided Fisher's exact test.

g-j. Volcano plots showing differential transposable element (TE) expression between *METTL3*^{KD} and CTRL cells in HT29 (**g**), COLO201 (**h**), HCT116 (**i**), and NCI-H716 (**j**) colorectal cancer (CRC) cells. Colour indicates TE density, and dotted lines denote the significance thresholds (false discovery rate (FDR) < 0.05 and |log2FC| ≥ 1) used to define differentially expressed TEs. Data represent two independent experiments.

k. m^{6}A dot blot analysis of total RNA isolated from the indicated colorectal cancer (CRC) cells. Equal amounts of total RNA from each CRC model were spotted onto Hybond N+ membranes, probed with an anti-m^{6}A antibody, and visualized by methylene blue staining. Representative dot blots from three independent experiments.

l. Schematic representation of m^{6}A RNA immunoprecipitation sequencing (MeRIP-seq). Created in BioRender. Mehdipour, P. (2026) https://BioRender.com/92bm54e.

m. Top enriched motif identified within m^{6}A peaks in HT29 and HCT116 cells. *P* values were calculated using a binomial test.

Source data are provided as a Source Data file.

Supplementary Fig. 6

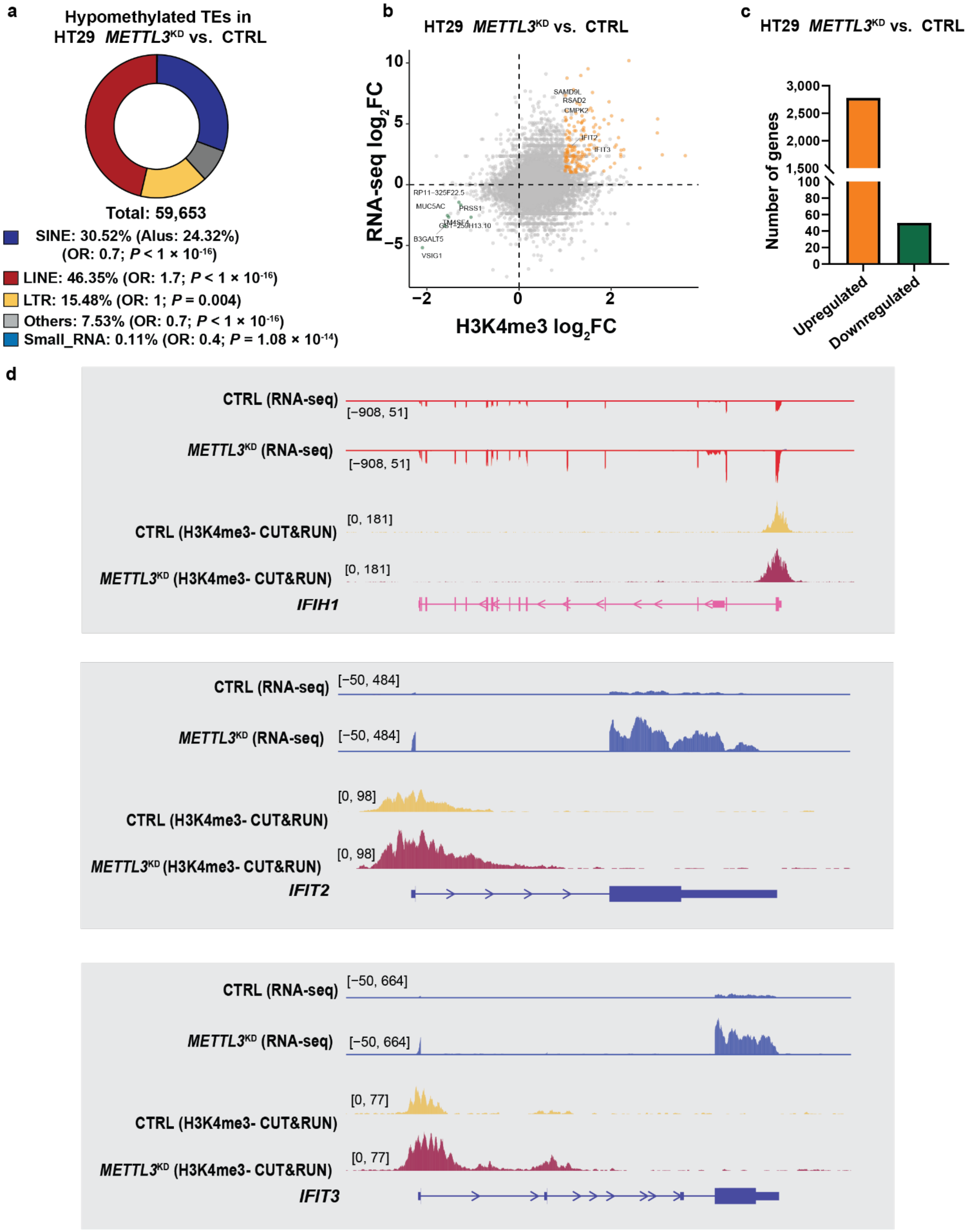

Supplementary Fig. 6. ISG upregulation following *METTL3* targeting is mediated by increased transcription.

a. Class representation of hypomethylated transposable elements (TEs) in *METTL3*^{KD} compared to CTRL HT29 cells. The odds ratio (OR) indicates enrichment or depletion of each category relative to the distribution of TEs annotated in the human genome. Statistical significance was determined by a two-sided Fisher's exact test.

b. Scatter plot comparing gene expression changes (RNA-seq; y axis) with changes in H3K4me3 occupancy at transcription start sites (CUT&RUN; x axis). Orange indicates genes that are upregulated with increased H3K4me3 occupancy; green indicates genes that are downregulated with reduced occupancy; grey indicates all others.

c. Bar plot showing the number of genes with significantly increased (orange) or decreased (green) H3K4me3 occupancy at their transcription start sites in *METTL3*^{KD} compared to CTRL HT29 cells. Significance was defined as false discovery rate (FDR) < 0.05 and |log2FC| ≥ 1.

d. Genomic tracks showing three representative interferon-stimulated genes (ISGs) with increased expression and elevated H3K4me3 occupancy at their transcription start sites in *METTL3*^{KD} compared to CTRL HT29 cells. For RNA-seq tracks, dark slate blue indicates sense transcription and red indicates antisense transcription. For H3K4me3 tracks, yellow denotes CTRL occupancy and dark red denotes *METTL3*^{KD} occupancy.

Source data are provided as a Source Data file.

Supplementary Fig. 7

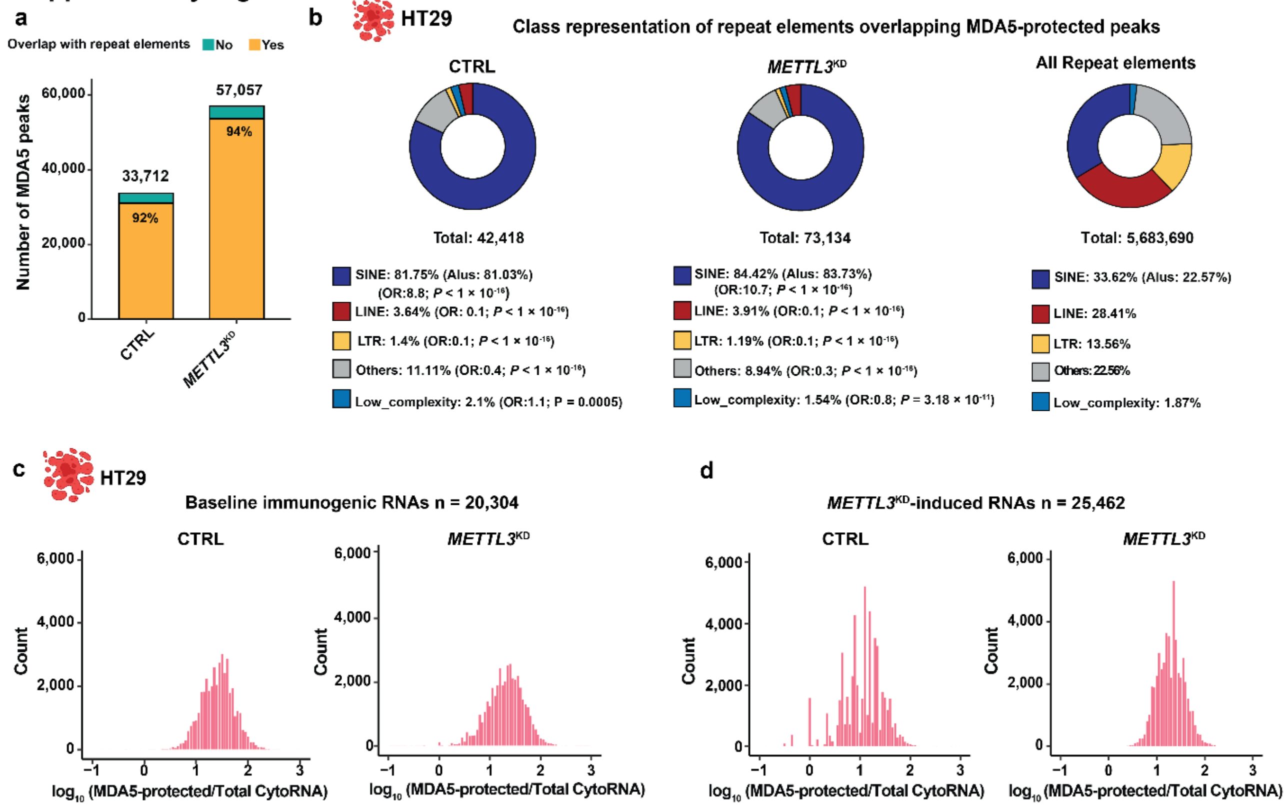

Supplementary Fig. 7. Loss of METTL3 promotes the formation of immunostimulatory dsRNAs derived from endogenous IR-Alus.

a. Stacked bar plot showing the number of MDA5-protected regions in *METTL3*^{KD} and CTRL HT29 cells. Percentage values within the bars indicate the proportion of MDA5-protected regions overlapping at least one repeat element.

b. Donut plots showing the class distribution of repeat elements overlapping MDA5-protected regions in CTRL and *METTL3*^{KD} HT29 cells.

c,d. Counts of expressed repeat elements shown as the $\log_{10}$-transformed fold change of MDA5-protected RNA relative to total cytoplasmic RNA (CytoRNA) expression in CTRL (left) and *METTL3*^{KD} (right) HT29 cells for baseline immunostimulatory RNA (n = 20,304) (**c**) and *METTL3*^{KD}-induced immunostimulatory RNA (n = 25,462) (**d**).

Source data are provided as a Source Data file.

Supplementary Fig. 8

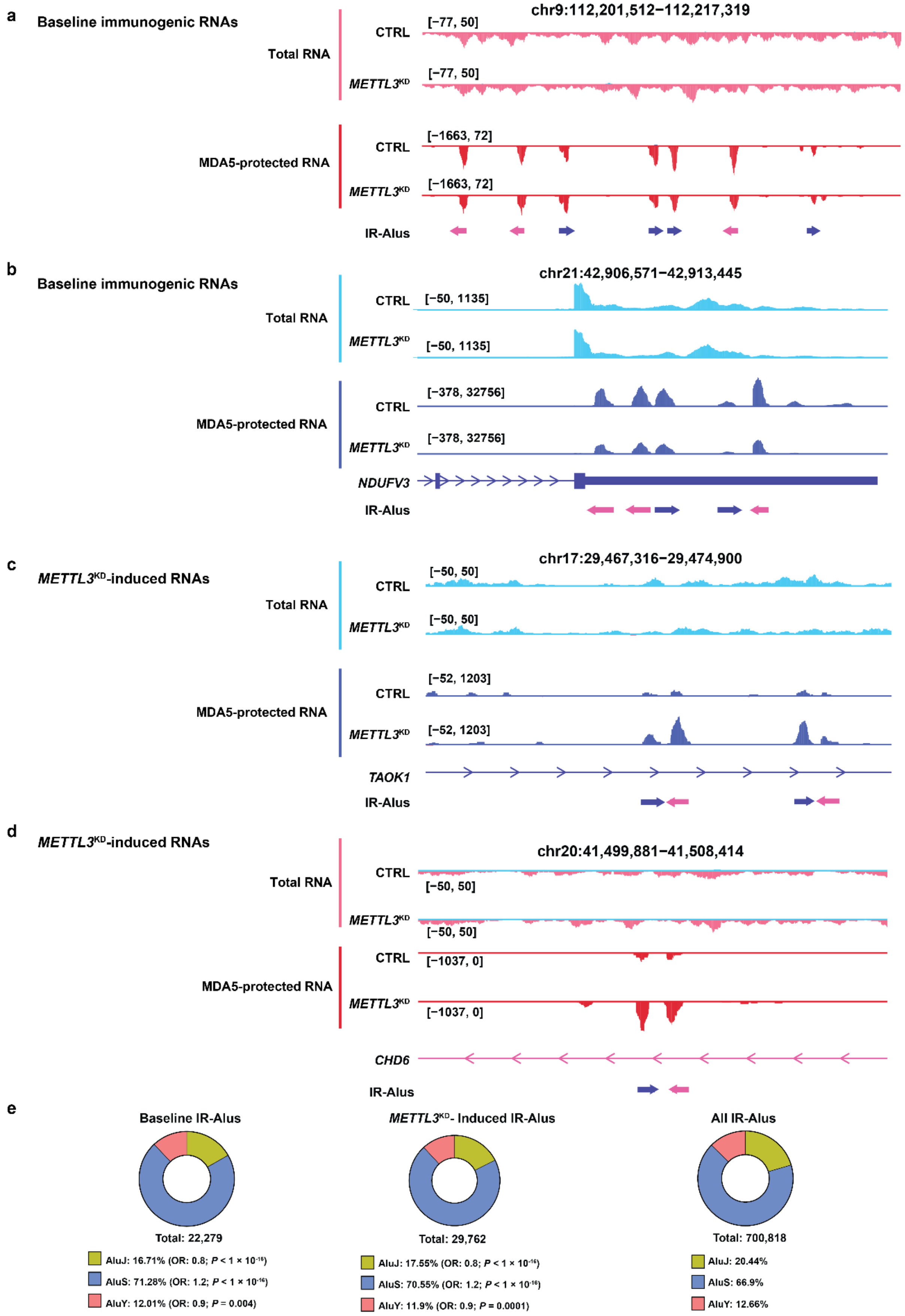

Supplementary Fig. 8. Representative inverted-repeat Alus contributing to dsRNA accumulation upon METTL3 depletion and their subfamily distribution.

a-d. Representative genomic tracks showing baseline (**a,b**) and *METTL3*^{KD}-induced (**c,d**) immunostimulatory inverted-repeat Alus located within intronic regions. The top two tracks show total cytoplasmic RNA, with sense (sky blue) and antisense (pink) transcription indicated. The bottom two tracks show RNase A–digested RNA, with sense (dark blue) and antisense (red) transcription indicated.

e. Distribution of Alu subfamilies upregulated in HT29 baseline IR-Alus and *METTL3*^{KD}-induced IR-Alus relative to all Alu elements annotated in the human genome (n = 1,145,669 Alu elements). The odds ratio (OR) indicates enrichment or depletion of each Alu subfamily (AluS, AluJ, and AluY) compared to the genome-wide Alu subfamily distribution. Statistical significance was determined by a two-sided Fisher's exact test.

Source data are provided as a Source Data file.

Supplementary Fig. 9

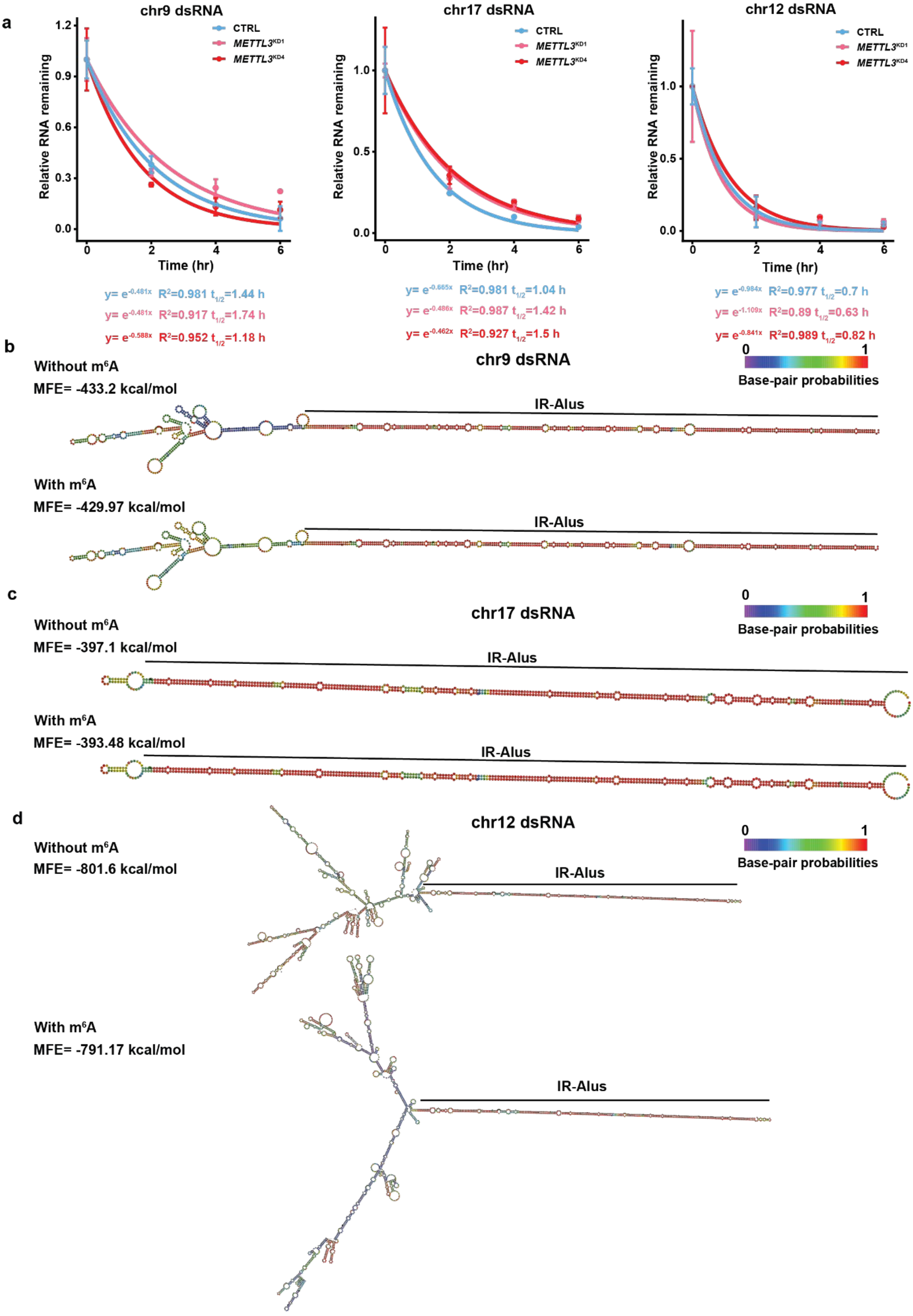

Supplementary Fig. 9.Actinomycin D chase analysis reveals no detectable change in stability of selected repeat-derived RNAs following METTL3 depletion.

a. HT29 CTRL and *METTL3*^{KD} cells were treated with actinomycin D, and RNA was extracted at the indicated time points for RT–qPCR analysis. Transcript decay was modelled using a first-order exponential fit ($y = e^{-kx}$). Fits with $R^2 \geq 0.95$ were considered robust. RNA half-lives were calculated as $t_{1/2} = \ln(2)/k$. n = 2 technical replicates.

b-d. Representative predicted dsRNA structures formed by IR-Alu pairs, shown either in their unmethylated form or with all adenosines substituted to mimic m^6A-modified positions. RNAfold-predicted secondary structures and corresponding minimum free energy (MFE) values are shown for IR-Alu loci on chromosomes 9, 17, and 12, respectively.

Source data are provided as a Source Data file.

Supplementary Fig. 10

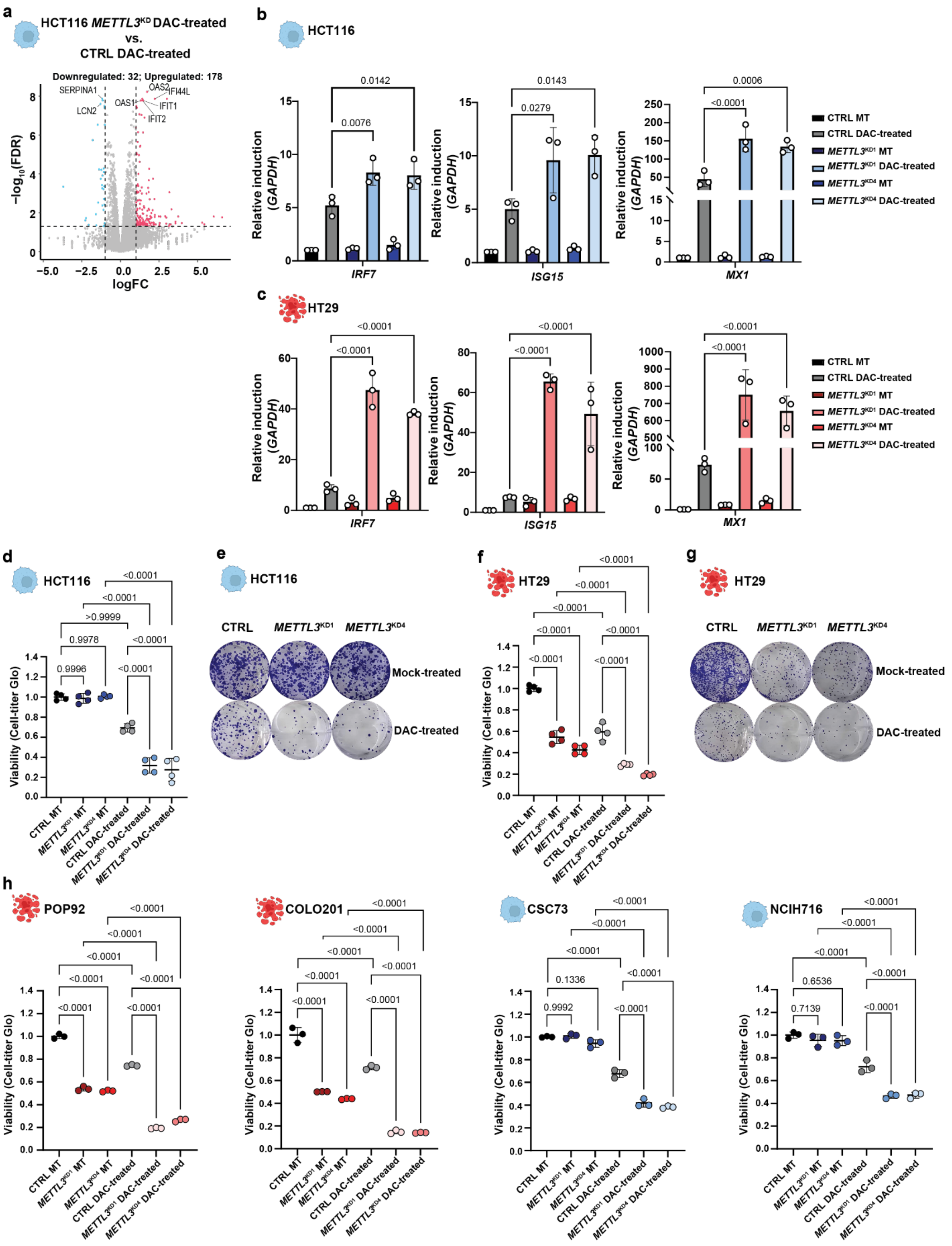

Supplementary Fig. 10. DNMT inhibition enhances ISG induction and reduces viability upon *METTL3* targeting in CRC cells

a. Volcano plot showing differential gene expression between *METTL3*^{KD} combined with DAC treatment and DAC treatment alone in HCT116 cells. Red indicates upregulated genes; blue indicates downregulated genes; grey indicates no significant change. Significance was defined as false discovery rate (FDR) < 0.05 and |log2FC| ≥ 1. Data represent two independent experiments

b,c. Quantitative PCR (qPCR) analysis of interferon-stimulated genes (ISGs; *IRF7*, *ISG15*, and *MX1*) in *METTL3* knockdown (^{KD1} and ^{KD4}) mock-treated (MT) and DAC-treated HCT116 (**b**) and HT29 (**c**) colorectal cancer (CRC) cells. qPCR data are presented as mean ± SD (n = 3 independent experiments). Statistical significance was determined by one-way ANOVA with Tukey's multiple-comparisons correction.

d. Viability of *METTL3* wild-type (CTRL) and *METTL3*^{KD} HCT116 colorectal cancer cells, as measured by the CellTiter-Glo luminescence assay. Data are presented as mean ± SD (n = 4 independent experiments). Statistical significance was determined by one-way ANOVA with Tukey's multiple-comparisons correction.

e. Colony-forming ability of HCT116 wild-type (CTRL) and *METTL3*^{KD} cells following mock treatment (top) or DAC treatment (bottom), as assessed by crystal violet staining.

f. Viability of *METTL3* wild-type (CTRL) and *METTL3*^{KD} HT29 colorectal cancer cells, as measured by the CellTiter-Glo luminescence assay. Data are presented as mean ± SD (n = 4 biological replicates). Statistical significance was determined by one-way ANOVA with Tukey's multiple-comparisons correction.

g. Colony-forming ability of HT29 wild-type (CTRL) and *METTL3*^{KD} cells following mock treatment (top) or DAC treatment (bottom), as assessed by crystal violet staining.

h. Viability of *METTL3* wild-type (CTRL) and *METTL3*^{KD} POP92, COLO201, CSC73, and NCI-H716 colorectal cancer cells, as measured by the CellTiter-Glo luminescence assay. Data are presented as mean ± SD (n = 3 independent experiments). Statistical significance was determined by one-way ANOVA with Tukey's multiple-comparisons correction.

Source data are provided as a Source Data file.

Supplementary Fig. 11

a

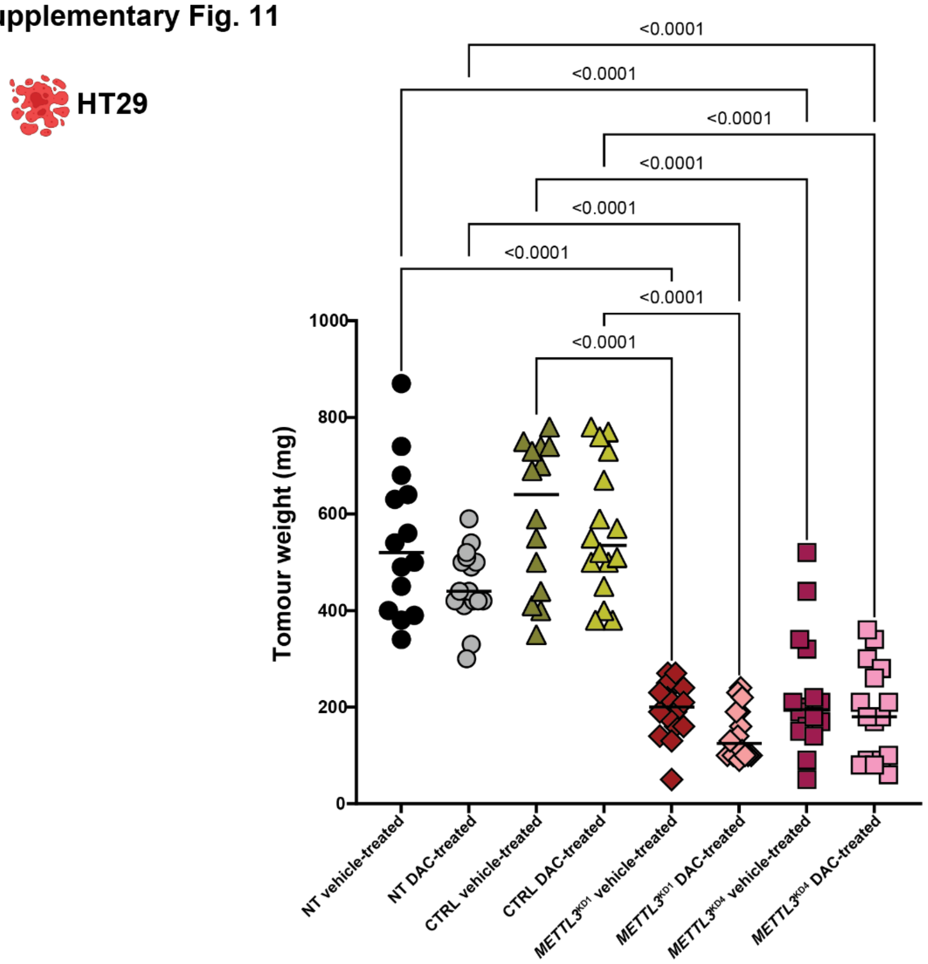

b

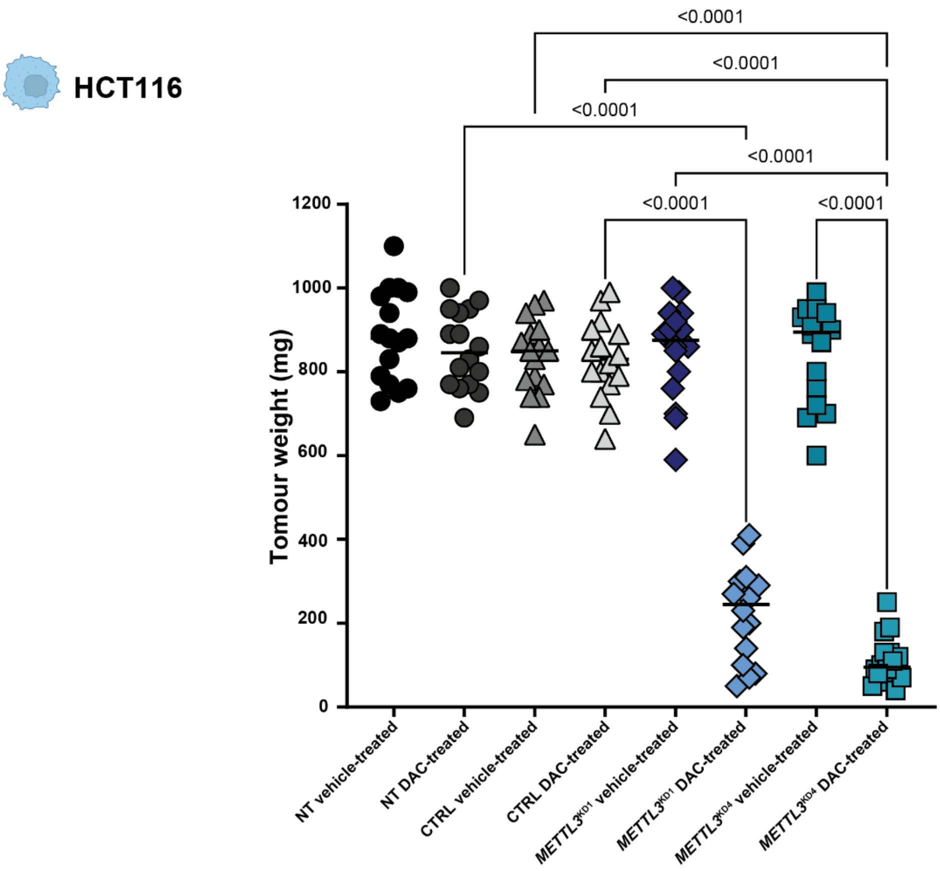

Supplementary Fig. 11. Combined DNMT inhibition and *METTL3* targeting suppress CRC tumour growth.

a. HT29 colorectal cancer (CRC) tumour weight (mg) in NSG mice for each group at the time of dissection (day 7 after the final treatment). Data are presented as mean ± SD. Statistical significance was determined by ordinary one-way ANOVA with Tukey's multiple-comparisons correction. n = 14 tumours for NT and CTRL vehicle-treated groups; n = 16 tumours for all other groups.

b. HCT116 colorectal cancer (CRC) tumour weight (mg) in NSG mice for each group at the time of dissection (day 9 after the final treatment). Data are presented as mean ± SD. Statistical significance was determined by ordinary one-way ANOVA with Tukey's multiple-comparisons correction. n = 16 tumours per group.

Source data are provided as a Source Data file.

Supplementary Fig. 12

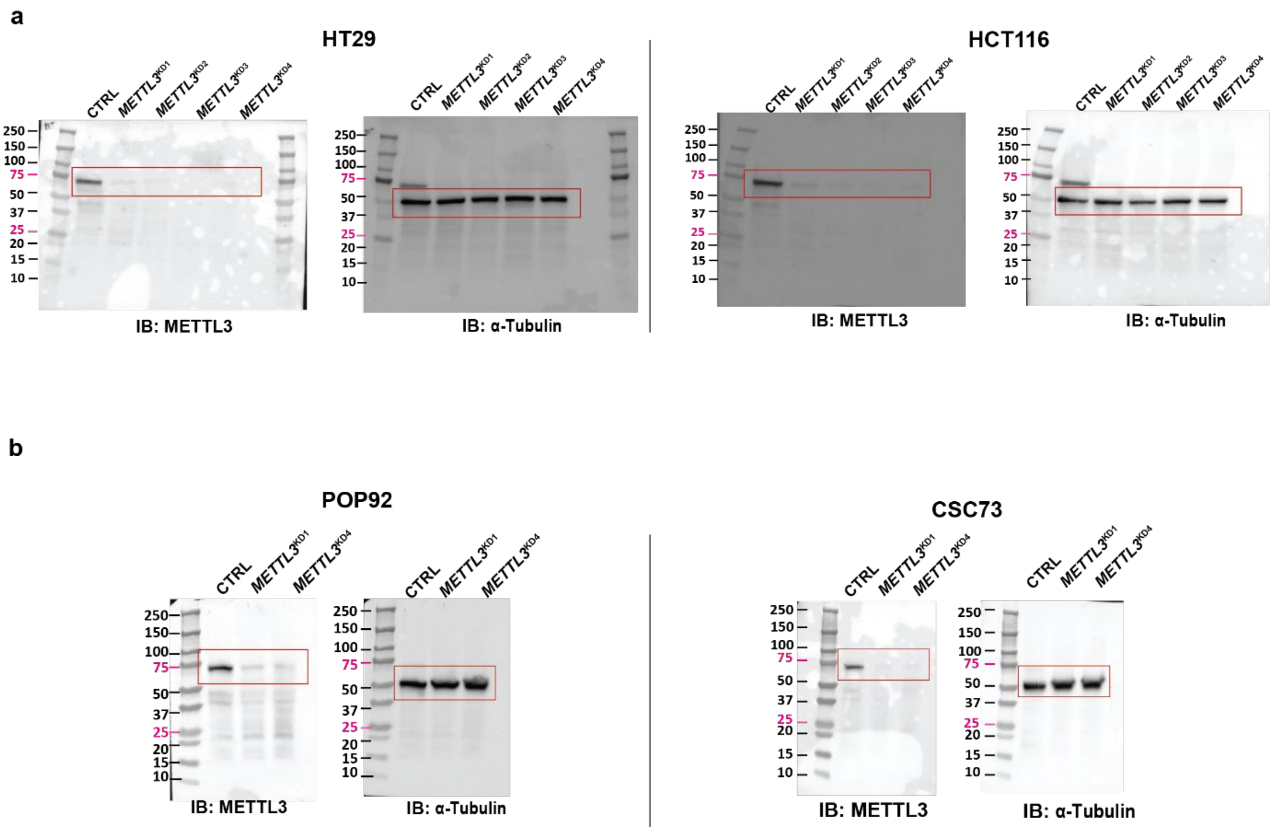

Supplementary Fig.12. Uncropped immunoblot images.

a,b. Full, uncropped immunoblot images corresponding to Supplementary Fig. 1a (**a**) and 1b (**b**) are shown. Molecular weight markers (Precision Plus Protein™ Dual Color Standards) are indicated; the 25 kDa and 75 kDa markers are shown in pink, and the remaining markers are shown in black. Immunoblots for METTL3 and α-tubulin loading controls are included. Boxes denote the regions presented in the supplementary figures.
