# Supplementary material for: m6A modification suppresses innate anti-tumour immunity in colorectal cancer by limiting alu-derived dsRNA accumulation
Source: Nat Commun. 2026 May 14;17:6428. doi: 10.1038/s41467-026-73211-z (PMC13377058; doi:10.1038/s41467-026-73211-z)
Supplement: Supplementary file 2 — Description of Additional Supplementary Files [file 41467_2026_73211_MOESM2_ESM.pdf]

Description of Additional Supplementary Files

Supplementary Data 1. List of 38 interferon-stimulated genes (ISGs).

Supplementary Data 2. Colorectal cancer consensus molecular subtypes.

Supplementary Data 3. The list of reagents and kits used in this study.

Supplementary Data 4. shRNA sequences used in this study.

Supplementary Data 5. qPCR primer names and sequences used in this study.

Supplementary Data 6. SgRNA and gRNA sequences used in this study.

Supplementary Data 7. Summary of colorectal cancer cell lines analyzed in this study.
