# Supplementary material for: m6A modification suppresses innate anti-tumour immunity in colorectal cancer by limiting alu-derived dsRNA accumulation
Source: Nat Commun. 2026 May 14;17:6428. doi: 10.1038/s41467-026-73211-z (PMC13377058; doi:10.1038/s41467-026-73211-z)
Supplement: Supplementary file 4 — Reporting Summary [file 41467_2026_73211_MOESM4_ESM.pdf]

nature portfolio

Corresponding author(s): Parinaz Mehdipour

Last updated by author(s): Mar 5, 2026

Reporting Summary

Nature Portfolio wishes to improve the reproducibility of the work that we publish. This form provides structure for consistency and transparency in reporting. For further information on Nature Portfolio policies, see our Editorial Policies and the Editorial Policy Checklist.

Statistics

For all statistical analyses, confirm that the following items are present in the figure legend, table legend, main text, or Methods section.

n/a	Confirmed	
☐	☒	The exact sample size (*n*) for each experimental group/condition, given as a discrete number and unit of measurement
☐	☒	A statement on whether measurements were taken from distinct samples or whether the same sample was measured repeatedly
☐	☒	The statistical test(s) used AND whether they are one- or two-sided<br>*Only common tests should be described solely by name; describe more complex techniques in the Methods section.*
☐	☒	A description of all covariates tested
☐	☒	A description of any assumptions or corrections, such as tests of normality and adjustment for multiple comparisons
☐	☒	A full description of the statistical parameters including central tendency (e.g. means) or other basic estimates (e.g. regression coefficient) AND variation (e.g. standard deviation) or associated estimates of uncertainty (e.g. confidence intervals)
☐	☒	For null hypothesis testing, the test statistic (e.g. *F*, *t*, *r*) with confidence intervals, effect sizes, degrees of freedom and *P* value noted<br>*Give P values as exact values whenever suitable.*
☒	☐	For Bayesian analysis, information on the choice of priors and Markov chain Monte Carlo settings
☒	☐	For hierarchical and complex designs, identification of the appropriate level for tests and full reporting of outcomes
☒	☐	Estimates of effect sizes (e.g. Cohen's *d*, Pearson's *r*), indicating how they were calculated

Our web collection on statistics for biologists contains articles on many of the points above.

Software and code

Policy information about availability of computer code

Data collection	Western blot images were acquired using a Bio-Rad ChemiDoc imaging system.L<br>ibraries for RNA-seq, CUT&RUN, MDA5 protection assay and MeRIP were sequenced on the Illumina NovaSeq 6000, NovaSeq X Plus and NextSeq 2000 platforms.<br>Quantitative real-time PCR was performed using a StepOnePlus Real-Time PCR System (Applied Biosystems).<br>DNA and RNA concentrations were measured using a NanoDrop ND-1000 spectrophotometer (Thermo Fisher Scientific).<br>Confocal images were acquired using a Zeiss LSM 980 and LSM 980 MP microscope.
Data analysis	GraphPad Prism v11.0.0 was used for the analysis of in vivo and in vitro phenotypic assays, graph generation, and statistical testing.<br>ImageJ v1.54g was used for the analysis of Immunofluorescence staining images.<br>Image Lab v6.1.0 (Build 7) was used for Western blot visualization.<br>ELDA software (https://bioinf.wehi.edu.au/software/elda/) provided by Walter and Eliza Hall Institute was used in order to calculated cancer initiating frequency for in vitro limiting dilution assay.<br>The statistical analyses on sequencing and public data were conducted in R (v4.3.2).<br>Total RNASeq analysis: trim_galore program (v0.6.2), STAR program (v2.7.9a), Picard program (v3.0.0), samtools (v1.8), subread (v1.6.4), bedtools (v.2.28.0), edgeR (v3.40.2), STRING website (v11.5), GSEA (v4.3.2), Cytoscape (v3.10.0), GSVA package (v1.46.0), and deepTools (v3.5.3).<br>MDA5-protection assays RNA-seq analysis: trim_galore program (v0.6.2), STAR program (v2.7.9a), Picard program (v3.0.0), samtools (v1.8), subread (v1.6.4), bedtools (v.2.28.0), MACS2 (v2.2.6), deepTools (v3.5.3), GenomicDistributions package (v3.17), and exonerate (v2.2.0).<br>MeRIP analysis: trim_galore program (v0.6.2), HISAT2 program (v2.2.1), Picard program (v3.0.0), samtools (v1.8), subread (v1.6.4), bedtools (v.2.28.0), HOMER software (v4.9.1), edgeR (v3.40.2), MACS2 (v2.2.6), GenomicDistributions package (v3.17), and deepTools (v3.5.3).

Cut&Run analysis: trim_galore program (v0.6.2), Bowtie2 (v.2.3.4.2), Picard program (v3.0.0), samtools (v1.8), subread (v1.6.4), bedtools (v.2.28.0), edgeR (v3.40.2), STRING website (v11.5), and deepTools (v3.5.3).
repeat elements analyis: SQuIRE (v0.9.9.9a-beta), edgeR (v3.40.2) and GenomicDistributions package (v3.17).
RNA secondary structures prediction: RNAfold WebServer (http://rna.tbi.univie.ac.at/cgi-bin/RNAWebSuite/RNAfold.cgi)
Consensus molecular subtyping: CMSclassifier R package (v1.0.0)
Mutation analysis of CRCs: maftools package (v2.18.0)
All scripts used for the analyses included in in this study, including differential expression analysis and IR-Alus identifications are publicly available in Zenodo at https://zenodo.org/records/18850943

For manuscripts utilizing custom algorithms or software that are central to the research but not yet described in published literature, software must be made available to editors and reviewers. We strongly encourage code deposition in a community repository (e.g. GitHub). See the Nature Portfolio guidelines for submitting code & software for further information.

Data

Policy information about availability of data

All manuscripts must include a data availability statement. This statement should provide the following information, where applicable:

- Accession codes, unique identifiers, or web links for publicly available datasets
- A description of any restrictions on data availability
- For clinical datasets or third party data, please ensure that the statement adheres to our policy

Data availability
All newly generated sequencing data from this study have been deposited in the Gene Expression Omnibus (GEO) database (https://www.ncbi.nlm.nih.gov/geo) under the accession number GSE289982.

Code availability
Scripts used for the analyses included in the manuscript are available on GitHub at https://github.com/yiluyucheng/m6A_dsRNA. DOI: 10.5281/zenodo.18850943
Source data are provided with this paper.

Research involving human participants, their data, or biological material

Policy information about studies with human participants or human data. See also policy information about sex, gender (identity/presentation), and sexual orientation and race, ethnicity and racism.

Reporting on sex and gender	Not reported in this study.
Reporting on race, ethnicity, or other socially relevant groupings	Not reported in this study.
Population characteristics	Human colorectal cancer patients undergoing surgical resection.
Recruitment	Human colorectal cancer tissue was obtained at the time of surgical resection with patient consent, as approved by the Research Ethics Board at the University Health Network, Toronto, Canada.
Ethics oversight	University Health Network

Note that full information on the approval of the study protocol must also be provided in the manuscript.

Field-specific reporting

Please select the one below that is the best fit for your research. If you are not sure, read the appropriate sections before making your selection.

☒ Life sciences ☐ Behavioural & social sciences ☐ Ecological, evolutionary & environmental sciences

For a reference copy of the document with all sections, see nature.com/documents/nr-reporting-summary-flat.pdf

Life sciences study design

All studies must disclose on these points even when the disclosure is negative.

Sample size	Group sizes were determined based on prior knowledge of variability, tumour growth kinetics, and response to DNA methyltransferase inhibitor treatment.<br>Roulois, D. et al. DNA-Demethylating Agents Target Colorectal Cancer Cells by Inducing Viral Mimicry by Endogenous Transcripts, Cell. 2015 Aug 27;162(5):961-73. doi: 10.1016/j.cell.2015.07.056. PMID: 26317465; PMCID: PMC4843502.<br>Mehdipour, P. et al. Epigenetic therapy induces transcription of inverted SINEs and ADAR1 dependency. Nature. 2020 Dec;588(7836):169-173. doi: 10.1038/s41586-020-2844-1. Epub 2020 Oct 21.
Data exclusions	No data was excluded from the analyses
Replication	All replication attempts were successful, as detailed in the Methods and figure legends.

Randomization	For in vivo experiments, mice were age-matched and randomized where appropriate, with equal numbers of male and female mice included in each group. No additional randomization was performed, as the remaining experiments were conducted in vitro using cell lines and PDX samples. For these experiments, cells were seeded from the same suspension across all relevant conditions to control for variability in seeding density.
Blinding	Data collection and analysis did not involve any blinding procedures. The investigators were not blinded since the collected data relied on quantitative analysis.

Reporting for specific materials, systems and methods

We require information from authors about some types of materials, experimental systems and methods used in many studies. Here, indicate whether each material, system or method listed is relevant to your study. If you are not sure if a list item applies to your research, read the appropriate section before selecting a response.

Materials & experimental systems

n/a	Involved in the study	
☐	☒	Antibodies
☐	☒	Eukaryotic cell lines
☒	☐	Palaeontology and archaeology
☐	☒	Animals and other organisms
☒	☐	Clinical data
☒	☐	Dual use research of concern
☒	☐	Plants

Methods

n/a	Involved in the study	
☒	☐	ChIP-seq
☒	☐	Flow cytometry
☒	☐	MRI-based neuroimaging

Antibodies

Antibodies used

METTL3 (D2I6O) Rabbit mAb #96391 (Cell Signaling Technology , Cat#96391S) 1:1000 Dilution, Lot#1
Monoclonal Anti-α-Tubulin antibody produced in mouse (Sigma-Aldrich, Cat# T9026) 1:3000 Dilution,Lot# 0000227464
Anti-mouse IgG, HRP-linked Antibody (Cell Signaling Technology , Cat#7076S) 1:5000 Dilution, Lot#36
Anti-rabbit IgG, HRP-linked Antibody (Cell Signaling Technology , Cat#7074S) 1:5000 Dilution, Lot#33
Anti-mouse IgG (H+L), F(ab')2 Fragment (Alexa Fluor® 647 Conjugate)(Cell Signaling Technology , Cat#4410S) 1:2000 Dilution, Lot#21
Anti-dsRNA monoclonal antibody J2, mouse, IgG2a, kappa chain (Jena Bioscience, Cat#RNT-SCI-10010500-JEN) 1:500 Dilution, Lot#18440
m6A Antibody (SYSY antibodies, Cat# 202 003) 1:1000 Dilution, Lot#5-155
Tri-Methyl-Histone H3 (Lys4) (C42D8) Rabbit mAb (Cell Signaling, #9751S) 1:50 Dilution, Lot#15
Rabbit (DA1E) mAb IgG XP® Isotype Control (Cell Signaling, #66362) 1:50 Dilution, Lot#3
Anti-PKR antibody [EPR19374] (Abcam, Cat#ab184257) 1: 2000 Dilution, Lot#1006611-7
RNase L Antibody (E-9): sc-74405 (Santa Cruz, Cat#sc-74405) 1:1000 Dilution, Lot#G0623
Anti-MAVS antibody (Abcam, Cat#ab89825) 1: 1000 Dilution, Lot#1082883-6
Vinculin Antibody (Invitrogen, # 700062) 1:3000 Dilution, Lot#2616511

Validation

For each assay (Western blot, immunofluorescence staining, and RNA dot blot), appropriate controls were included alongside knockdown, knockout, or DNMTi-treated samples on the same blot. Manufacturer validation statements are provided below.
METTL3: https://www.cellsignal.com/products/primary-antibodies/mettl3-d2i6o-rabbit-mab/96391?srsltid=AfmBOormct-MaKXzfpd1_scWoT9Tfu0NDBsSmgyLZt8AUYCPztWUTGOc
α-Tubulin: https://www.sigmaaldrich.com/GB/en/product/sigma/t9026?srsltid=AfmBOopKOmurAr_UoI3E8m06pz_-JYTHFZ1sOBNusZpDDEv5w_fQPOXF
Anti-mouse IgG, HRP-linked Antibody: https://www.cellsignal.com/products/secondary-antibodies/anti-mouse-igg-hrp-linked-antibody/7076
Anti-rabbit IgG, HRP-linked Antibody: https://www.cellsignal.com/products/secondary-antibodies/anti-rabbit-igg-hrp-linked-antibody/7074
Anti-mouse IgG (H+L), F(ab')2 Fragment: https://www.cellsignal.com/products/secondary-antibodies/anti-mouse-igg-h-l-f-ab-2-fragment-alexa-fluor-647-conjugate/4410?srsltid=AfmBOopQgreLybt6jklmT8ZybkGRGKzX1zqdHDHuTWHqUgKcAvqYaxr3
Anti-dsRNA monoclonal antibody J2: https://www.jenabioscience.com/rna-technologies/rna-analysis-detection/dsrna-detection/rnt-sci-10010-anti-dsrna-monoclonal-j2
M6a Antibody: https://sysy.com/product/202003
Tri-Methyl-Histone H3 (Lys4) (C42D8) Rabbit mAb: https://www.cellsignal.com/products/primary-antibodies/tri-methyl-histone-h3-lys4-c42d8-rabbit-mab/9751?srsltid=AfmBOoqUEJR9co4aoYVchwLWHGEQVD3wdy-0OxW3DL11TU6oxY4xhold
Rabbit (DA1E) mAb IgG XP® Isotype Control : https://www.cellsignal.com/products/primary-antibodies/rabbit-da1e-mab-igg-xp-isotype-control-cut-amp-run/66362?srsltid=AfmBOoq4IY_i9wMrt9EwX9bJwlc_GVAGoy6WKqcSnipY-qHu6noITpr7
PKR:https://www.abcam.com/en-us/products/primary-antibodies/pkr-antibody-epr19374-ab184257

RNaseL: https://www.scbt.com/p/rnase-l-antibody-e-9?srsltid=AfmBOoqnzM2XBrsWC1-XypE-T91gVCxSVtlJ6n8QyQ8n8Kv_B81ULiNr
MAVS: https://www.abcam.com/en-us/products/primary-antibodies/mavs-antibody-ab89825
Vinculin: https://www.thermofisher.com/antibody/product/Vinculin-Antibody-clone-42H89L44-Recombinant-Monoclonal/700062

Eukaryotic cell lines

Policy information about cell lines and Sex and Gender in Research

Cell line source(s)	CCD 841 CoN (CRL-1790™, Cat# ATCC-CRL-1790), HCT 116 (Cat# ATCC-CCL-247), NCI-H716 (H716; Cat# ATCC-CCL-251), and COLO 201 (Cat# ATCC-CCL-224) were purchased from the ATCC. HT29 cells were kindly provided by Prof. Colin Goding. p125 HEK IFNβ WT and MAVS KO cells were kindly provided by Prof. Jan Rehwinkel. POP92 and CSC73 colon cancer xenograft cells were kindly provided by Dr. Catherine O'Brien.
Authentication	HT29 cells were authenticated by STR (Short Tandem Repeat) DNA profiling
Mycoplasma contamination	Cells were routinely tested for mycoplasma contamination, and only mycoplasma-free cells were used in all experiments.
Commonly misidentified lines (See ICLAC register)	No commonly misidentified cell lines were used in this study.

Animals and other research organisms

Policy information about studies involving animals; ARRIVE guidelines recommended for reporting animal research, and Sex and Gender in Research

Laboratory animals	4- to 5-week-old male and female NSG (NOD.Cg-PrkdcSCID Il2rgtm1Wjl/SzJ) mice were purchased from Charles River. All cages were on a 12 h:12 h light: dark cycle (lights on, 07:00) in a temperature-controlled and humidity-controlled room. Room temperature was maintained at 19-23 °C, and room humidity was maintained at 45–65%.
Wild animals	This study did not involve wild animals.
Reporting on sex	Equal numbers of male and female mice were used in each experiment.
Field-collected samples	No field-collected samples were used in this study.
Ethics oversight	in vivo experiments were carried out in accordance with the terms of the UK Animals (Scientific Procedures) Act Project License (PPL) (PP7109477).

Note that full information on the approval of the study protocol must also be provided in the manuscript.

Plants

Seed stocks	N/A
Novel plant genotypes	N/A
Authentication	N/A
