# Supplementary material for: m6A modification suppresses innate anti-tumour immunity in colorectal cancer by limiting alu-derived dsRNA accumulation
Source: Nat Commun. 2026 May 14;17:6428. doi: 10.1038/s41467-026-73211-z (PMC13377058; doi:10.1038/s41467-026-73211-z)
Supplement: Supplementary file 5 — Transparent Peer Review file [file 41467_2026_73211_MOESM5_ESM.pdf]

nature portfolio

Peer Review File

m6A Modification Suppresses Innate Anti-Tumour Immunity in Colorectal Cancer by Limiting Alu-Derived dsRNA Accumulation

Corresponding Author: Dr Parinaz Mehdipour

This file contains all reviewer reports in order by version, followed by all author rebuttals in order by version.

Version 0:

Reviewer comments:

Reviewer #1

(Remarks to the Author)
The manuscript by Wang et al. focused on the m6A RNA methyltransferase METTL3 as a retroelement suppressor forming immunostimulatory dsRNA in colorectal cancer. The authors used mainly two cell lines, HT29 and HCT116, which showed significant different effects of METTL3 downregulation (via shRNAs). The METTL3 downregulation in HT29 led to higher amount of DEGs and dsRNA. A validation was performed with two additional cell lines. Overall, the authors identified the source of dsRNA as mainly SINE (>43 %) with Alu inverted repeats. However, LINE were found >31 % overexpressed, which would have been interesting to investigate further, also under the aspect, if SINEs were significantly higher expressed in respect to LINE (Suppl. Fig. 3) and did this justify to include Alu in the title? The authors further showed that METTL3 loss elevated expression of ISGs. Mice experiments with HT29 and HCT116 plus/minus METTL3 knockdown (shRNA) plus/minus Decitabine (demethylation) led to different tumor formations.

Although a well-rounded study with an employment against colorectal cancer, the author's results are somehow lessened in their novelty due to various prior publications (also cited as manuscript references), which I want to mention since some correlate with and confirm the results from Wang et al:
1) METTL3 and m6A incorporation modulates A-to-I editing (ADAR) (Xiang et al. 2018 Mol Cell)
2) M6A plays a role in development (see ref. #21)
3) M6A regulates other RE, like ERVs (#18)
4) METTL3 and IFN response, also with anti-PD1 (#22)
5) METTL3 m6A dsRNA and immunology (#38)
6) Inv repeat Alus (AIR) bind MDA5 (#32)
7) MDA5 induces a Z-RNA conformation of Alus for ADAR1 (Nichols et al. 2021, Nat Commun)
8) Alu inv rep (AIR) is the most common source of dsRNA (#32)
9) METTL3 methylates ADAR1 (Tassinari et al. 2021 Genome Biol)
10) METTL3 regulate tumor immune m6A (Zhu et al. 2023 Cell Oncol)
11) METTL3 acts as an oncogene in colorectal cancer (Li et al. 2019, Mol Cancer)
12) PKR binds AIR (Kim et al. 2014, Genes & Dev)
13) Inhibition of DNMT (DNMTi) induces SINE (#9)

Specific remarks and questions:
1) Ln 121: which HCT116 cells were used, as there are many different available strains at ATCC?
2) Ln 121 and ln 177, Table 2: please combine the cell lines with the CMS in Table 2, especially considering the RAS mutations (HCT116: KRASG13D; NCI-H716 and COLO201 RAS wt etc.)
3) Ln 274-279: The reference #32 was used for the RNase/MDA5 protection assay, although the reference #32 also claims that MDA5 wt has only limited binding capabilities of Alu-Alu hybrids (dsRNA), due to mismatches, bulges and A-to-I; in contrast to mutated MDA5. This could be a limitation of the Alu conclusions; please discuss.
4) Ln 291 and Fig. 4F: generally, IR-Alus were found; were any of the Alu subfamilies (AluY, AluJ, AluS) dominant?
5) Ln 344 and 678: Why were different amounts of HT29 (500,000 cells) and HCT116 (250,000 cells) for NSG mice injections used?
6) Ln 348 and Fig. 9: How many cells were injected with METTL3KD1 etc. Explain METTL3KD1/4 (KD1-4?), as Fig. 1 has

KD1-4 and Fig. 6 has only METTL3KD1.
7) Ln 380: Please explain “accumulation of dsRNA, rather than increased mRNA stability” in more detail. Is this really an exclusion rule? Which proof do the authors have for either or?
8) Ln 419: Decitabine is a new avenue for treatment, although other inhibitors (Ln 418) were not tested here. This is a too strong conclusion and judgement.
9) Ln 492: Was DNase I used for RNA isolations?
10) Ln 500: It is a general standard that for qPCR two reference genes have to be used (not only GAPDH), esp. due to the fact that GAPDH concentrations vary between individuals, development, cell cycle etc. (Bustin SA 2000, J Mol Endocrinol).

(Remarks on code availability)

Reviewer #2

(Remarks to the Author)
In this manuscript submission to Nature Communications, Wang, Y et al., present compelling data supporting a tumor promoting role of the METTL3 gene, often elevated in cancer, in suppressing tumor cell-intrinsic antiviral responses against transposable element-associated double-stranded RNA in colorectal cancer. This work has important implications for therapeutically enhancing the pro-apoptotic responses against transposable elements and double-stranded RNA in this cancer, especially in light of recent development and clinical trials of METTL3 inhibitors in solid tumors. The authors demonstrate that sensitivity to inhibition of METTL3 is variable in human colorectal cancer cell lines and dependent on basal levels of retroelement expression, but that even in those lines that are insensitive to inhibition, synergy with METTL3 inhibition can be achieved by cotreatment with a DNA methyltransferase inhibitor (Decitabine) that also increases retroelement expression. These findings, if further validated, are both timely and potentially clinically impactful.
However, there are two major issues with the manuscript that currently temper enthusiasm: 1) most of the mechanistic work is performed in only one METTL3 knockdown sensitive (HT29) and insensitive (HCT116) colorectal cancer cell line. These findings should be validated in additional colorectal cancer cell lines to warrant publication in a rigorous journal such as Nature Communications. 2) The authors fail to cite critical prior research in the area of METTL3’s role in modifying mRNA transcripts and viral mimicry, including highly relevant publications that have explored this concept specifically in CRC. It is therefore challenging to accurately evaluate the novelty of some of the key findings. Which findings are corroborative of prior studies of METLL3 in CRC, which findings are derivative of known roles of METTL3 in other contexts (i.e., similar findings but not specifically in CRC), and which findings are novel? A nuanced discussion of the above questions, inclusive of relevant citations, should be provided throughout the Introduction, Results section, and in the Discussion.

Specific comments are included below:

1)

114-116: “This analysis revealed that METTL3 expression is elevated in cancerous tissues compared to corresponding normal tissues in the majority of cancers, including colorectal adenocarcinoma (COAD).”

It is essential to cite prior literature here, as this has previously been reported in different cancers, including CRC (e.g., Pan, J, et al., Journal of Experimental and Clinical Cancer Research, 2022; Tang, T, et al., Cellular Signaling, 2022; etc.).

2)

124-125: “Collectively, these findings suggest that CRC cells exhibit varying vulnerabilities to METTL3 depletion.”

It is also important to cite prior literature here, as previous reports exist of antitumor effects of knockout and knockdown of METTL3 in CRC, including in the HCT116 line used in the current study (e.g., Pan, J, et al., Journal of Experimental and Clinical Cancer Research, 2022; Yang, Z, et al., Signal Transduction and Targeted Therapy, 2021; etc.).

3)

Can the authors reconcile the results of the previous studies reporting a decrease in viability in HCT116 with targeting METLL3 with their own results showing no effect (Figure 1C)? Could this perhaps be due to poor knockdown efficiency in HCT116? KD3 and KD4 are most potent in HT29 and also achieve much deeper RNA knockdown than in HCT116, so it is possible that more complete knockdown (or knockout) would have a phenotype in HCT116, as previously reported. CRISPR-Cas9 (or other) knockout (as opposed to knockdown) strategies should be considered.

Given the above, this line in the introduction is an overstatement and not accurate:

98-99: “However, the vulnerability of CRC to METTL3 targeting and the underlying mechanisms remain unexplored.”

Specifically, there are multiple published studies exploring the vulnerability of CRC to METTL3 targeting, and it is quite clear that METTL3 levels are elevated in CRC and knockout/knockdown reduces CRC viability in vitro and in vivo. However, the

mechanisms underlying this vulnerability, particularly as relate to RE and antiviral responses as proposed in this study, remain incompletely understood. The current study, therefore, has novelty, but should not overstate the novelty of the above central observations or ignore the important literature already published in this space.

4)

111: “Colorectal cancer cells show different sensitivity to METTL3 targeting”

Once again, the results presented in this section fail to cite the prior literature, which supports the same conclusions through similar analyses.

5)

228-229: “METTL3 loss drives ISG activation in m6A-high CRC cells via transcriptional regulation”

Can these results be validated in the other lines (COLO201, NCI-H716, POP92, CSC73)? Given that basal levels of RE expression is being proposed as a key determinant of sensitivity to METLL3 KO in CRC, it is important to validate this in more than n = 1 cell line.

6)

223-226: “Moreover, basal RE expression may serve as a predictive signature for determining cancer cell sensitivity to METTL3 inhibition. This novel insight could provide new avenues for identifying CRCs more likely to respond to METTL3 inhibition based on their baseline RE expression profiles.”

Given that there is only one sensitive and one insensitive cell line evaluated in this analysis (Figure 4F), it is impossible to assess this hypothesis with any statistical certainty. Additional cell lines must be included to be able to make this statement with any confidence—must have at least n = 3 sensitive and n = 3 insensitive, which the authors have already identified with the additional lines shown in Figure 3.

7)

228-229: “METTL3 loss drives ISG activation in m6A-high CRC cells via transcriptional regulation”

Again, for any meaningful conclusions to be drawn from this section, additional cell lines must be included and statistical tests performed.

8)

277-279: “SINEs, particularly Alu elements, constituted the majority of MDA5-protected regions, and METTL3KD significantly increased the enrichment of these regions in HT29 CRC cells (Supplementary Figure 5B).”

Supplementary Figure 5B does not show any significant enrichment of these regions in METTL3 KD versus WT. Again, statistics are impossible with only a single line in each group, and the difference is minor and could be completely within the error of the assay or variance between lines. Once again, the conclusions presented in this section are uncertain in the absence of validation in other lines and rigorous statistical analysis.

9)

297-299: “This phenomenon appears to depend, at least in part, on METTL3 activity, whereby METTL3-mediated m6A modifications inhibit the formation of dsRNA from transcriptionally active REs. To test this hypothesis . . . ”

Can references be provided here? This appears to be a well-established concept, albeit not necessarily the context of CRC.

10)

313-317: “Collectively, our results demonstrate that METTL3 suppresses the viral mimicry responses by inhibiting the folding of IR-Alu transcripts into dsRNA structures (Figure 7D). METTL3KD In METTL3-sensitive CRCs with higher basal RE expression and m6A modifications induces dsRNA formation, triggering viral mimicry and cancer cell death. In contrast, METTL3KD in METTL3-insensitive CRCs, characterized by lower RE expression and m6A modifications, fails to induce cancer cell death.”

Again, this needs to be validated in additional lines.

11)

331-333: “Additionally, viability assays using CELL-TITER-Glo and colony-forming assays demonstrated that DAC treatment induced a synthetic lethality with METTL3 inhibition in both CRC cells (Supplementary Figures 7D-G).”

A statistical test for synergy (e.g., non-additive increase) should be performed to prove this. It appears this may be true for HCT116, but likely not HT29. What about the other sensitive and insensitive lines? Again, there must be validation in additional lines to prove that these effects are not idiosyncratic differences between HT29 and HCT116.

12)

The presentation of Figures 1 and 3 feels disjointed, given that they make the same point but in a different set of cell lines. Figure 3 should not be a separate figure, but rather should be combined with Figure 1/Supplemental Figure 1.

13)

Figure 4B-C could ideally be combined into one panel, with shape (e.g., circle versus triangle) denoting MSI status. No need for two separate panels.

14)

Figure 4D is more suited to supplementary figures.

15)

Figure 4E is not particularly informative as presented. Choosing the top 20 genes based on mutation frequency tends to enrich for many non-relevant/non-driver genes that are simply very large and thus neutrally accumulate many mutations (e.g., TTN, OBSCN). Inclusion of these genes may obscure potentially meaningful differences. If the authors really want to make the point that there is no difference in mutational profile, a more earnest analysis should be performed. For example, prioritization of significantly recurrently mutated driver genes should be performed by using MutSigCV (Lawrence, MS, et al., Nature, 2013) (such analysis has been previously performed in CRC and the driver gene list(s) can be pulled from these studies) and only these included in the analysis. Then, a gene-by-gene analysis of mutation rates in DepMap METLL3 KO sensitive versus insensitive cell lines should be performed to identify any mutant gene-specific enrichments.
Relatedly, only 4 of the cell lines, albeit the ones included in the experiments in this study (COLO201, HT29, HCT116, NCIH716) are highlighted in the plot. Can the authors plot the cell line key bar to show sensitive versus insensitive lines? While it is noted in the legend that the lines are ordered by sensitivity score, where do the insensitive end and the sensitive begin (based on the dotted line in Figure 4B-C)? If there are truly no patterns to report here, this figure should be put in the supplementary figures.

16)

391-393: “In cancer cells, however, targeting METTL3 and the loss of m6A induces conformational changes in these IR-Alus, enabling the formation of dsRNA stem-loops and ultimately leading to dsRNA accumulation.”

Were conformational changes experimentally demonstrated, or are they being assumed?

(Remarks on code availability)

Reviewer #3

(Remarks to the Author)
This manuscript presents a well-executed study investigating how METTL3-mediated m6A RNA modification suppresses viral mimicry responses in colorectal cancer by preventing dsRNA formation from Alu repeats.
Major Comments
1. While the authors demonstrate that m6A loss enables IR-Alu folding into dsRNA, additional structural or biophysical studies could provide deeper insights into how m6A modifications specifically prevent these secondary structure formations, such as DMS-seq or SHAPE-related method to determine the RNA structural maps.
2. Similarly, dsRNA was detected via RNase/MDA5 protection assays. Could transcriptome-wide RNA-structure profiling (e.g. chemical probing and sequencing) be employed? Given the intronic enrichment of dsRNA signals post-treatment (Fig

6D), precise mapping and in-depth characterization of their genomic origins would strengthen the study.
3. METTL3 knockdown may affect other RNA metabolism pathways beyond dsRNA formation. The authors should clarify whether the observed cell death is primarily due to viral mimicry or involves additional stress response mechanisms.
4. METTL3 carries widespread beneficial functions, so its direct inhibition may elicit unintended adverse effects. In the authors' previous work (ref 9, Nature), targeting ADAR1 was shown to represent a promising strategy for enhancing antitumor immunity; unlike ADAR1, METTL3 lacks intrinsic dsRNA-specific binding capacity. Cell-level assays alone cannot reveal safety impacts at the tissue, organ, or organismal level.
5. The basis for selecting a DNMT inhibitor (DNMTi) and its mechanism of enhancement are not experimentally validated. The choice appears based only on literature precedent rather than systematic screening, and a DNMTi-only control is missing.
Minor Comments：
1. Pg. 34, line 22, "The top 20 most mutated genes " showed "21 genes ".
2. MeRIP-seq Technical Issue (Lines 708-711),The observation that "the first read from paired-end sequencing exhibited lower sequencing quality" in MeRIP samples is indeed unusual and warrants further explanation.
3. The Introduction does not justify the focus on colorectal cancer—e.g. its prevalence, urgency of progression, or treatment challenges. Figure S1A shows that METTL3 is overexpressed across most cancer types. To support broad applicability, additional cancer cell lines should be evaluated.
4. Line 62: Please add an appropriate reference.
5. Line 197: This statement lacks supporting data or statistical evidence; please provide a figure or analysis.
6. Figs 1A, 4B & 4C: Provide supplementary tables with the underlying data so readers can examine additional cell lines of interest.

(Remarks on code availability)

Reviewer #4

(Remarks to the Author)
In this manuscript, the authors elucidate a mechanism by which inhibition of METTL3 increases dsRNA levels, thereby activate the immune response. They demonstrate that METTL3 suppresses dsRNA formation particularly that derived from IR-Alus, via m6A methylation in colorectal cancer. They investigate that CRC cells exhibiting higher basal levels of retroelements and m6A modification display greater dependency on METTL3. Importantly, they find that activating REs using DNMTi can sensitize previously insensitive CRC cells to METTL3 targeting, suggesting a promising treatment of combination therapy strategy.

Overall, this is a well-written and presented study that propose a promising therapeutic approach CRC. However, a significant concern is the similarity to the findings reported by Yimeng Gao et al. 1 , who also suggested that m6A modification mediated by METTL3 prevents formation of endogenous double-stranded RNA (dsRNA). The authors should thoroughly address this by comparing the two studies in discussion section, specifically highlighting the contribution of IR-Alu among dsRNAs in CRC.

I have a few additional comments that should be addressed.
1. Line 132, is the minimal effect of METTL3 ko in HCT116 cells reliable? Chaoqin Shen et.al2 showed significant transcriptomic changes ("a total of 2848 downregulated genes and 3046 upregulated genes ($p < 0.05$) were detected (raw data accessible via GEO number GSE130012) after knockout of METTL3 in HCT116 cells"). Do other insensitive cell lines such as NCI-H716 and 183 CSC73 cells also exhibit similar minimal effects following METTL3 KO?
2. For IFNβ promoter luciferase reporter assay, authors used total RNA isolated from cells. Using MDA5-captured dsRNA would provide more direct evidence.
3. The authors propose that higher basal expression of REs and m6A is major reason of sensitivity of CRCs to METTL3 targeting. Do these sensitive CRCs also express higher ISGs compared to the insensitive cells?
4. Figure 4F show higher expressed TEs in HT29 compared to HCT116 (82680 vs 18712). However, the volcano plot appears to show only imperceptible changes; In addition, does different TEs show different expression pattern? Supplementary Figures 3C and 3D should show expression changes for different TE loci, especially Alu elements.
5. Why does targeting METTL3 specifically induce IR-Alus? Does the METTL14/3 specifically bind Alu RNA?
6. Are all IR-Alu molecules mentioned in this manuscript derived from embedded Alu elements transcribed by pol II within host gene transcripts? Does METTL3 KO regulate RNA stability of host genes containing these Alu elements?

References
1. m6A Modification Prevents Formation of Endogenous Double-Stranded RNAs and Deleterious Innate Immune Responses during Hematopoietic Development. Immunity 52, 1007-1021.e8 (2020).
2. Shen, C. et al. m6A-dependent glycolysis enhances colorectal cancer progression. Mol. Cancer 19, 1–19 (2020).

(Remarks on code availability)

Version 1:

Reviewer comments:

Reviewer #1

(Remarks to the Author)
The revised MS by Wang et al. addresses most of the questions and comments by Reviewer 1; esp. concerning the specific remarks 1-10. Concerning the specific remark #7 regarding „dsRNA accumulation rather than stability“ the authors did many additional experiments (Fig. 2 in reviewer comments). However, the use of RNAfold (which did not reveal substantial differences between methylated and unmethylated RNA) is not sufficient and actually inadequate for this question. Wang (ref #43) which is only used in ln273 as a common reference showed that the binding of YTHDF2, as well as IGF2BPs (see review by Wei G 2024, Biochem Soc Trans) are responsible for the stability of m6A RNA. The authors should consider these RNA-protein interactions, which also apply to dsRNA as shown by Yang et al. (2025, Nature Commun) rather than RNAfold studies.
In summary, the present study by Wang et al. demonstrates a thorough investigation about m6A RNA, dsRNA and METTL3 in colorectal cancer cell lines. It is important to confirm known cellular processes between different cancer types using cell lines. However, the reply by the authors to all other publications showing a similar importance of m6A, dsRNA, METTL3 with innate immunity (main questions 1-13 for reviewer 1) was always „colorectal cell line and Alu dsRNA“. This is in my opinion not sufficient for Nature Communications, even after a novel paper in PNAS (Li Y et al. 2025), which showed that next to LINE and ERV, also Alus are enriched in METTL3 knock-out cells (although in breast cell lines).

(Remarks on code availability)

Reviewer #2

(Remarks to the Author)
The authors have addressed all of my concerns and the manuscript is significantly strengthened. I recommend it for publication at Nature Communications.

(Remarks on code availability)

Reviewer #3

(Remarks to the Author)
Thank you for the revised manuscript. The authors have addressed all my concerns.

(Remarks on code availability)

Reviewer #4

(Remarks to the Author)
The authors have properly addressed all my concerns and adequately revised the manuscript. I recommend it for publication.

(Remarks on code availability)

Open Access This Peer Review File is licensed under a Creative Commons Attribution 4.0 International License, which permits use, sharing, adaptation, distribution and reproduction in any medium or format, as long as you give appropriate credit to the original author(s) and the source, provide a link to the Creative Commons license, and indicate if changes were made.
In cases where reviewers are anonymous, credit should be given to 'Anonymous Referee' and the source.
The images or other third party material in this Peer Review File are included in the article's Creative Commons license, unless indicated otherwise in a credit line to the material. If material is not included in the article's Creative Commons license and your intended use is not permitted by statutory regulation or exceeds the permitted use, you will need to obtain permission directly from the copyright holder.
To view a copy of this license, visit https://creativecommons.org/licenses/by/4.0/

REVIEWER COMMENTS

Reviewer #1 (Remarks to the Author):

The manuscript by Wang et al. focused on the m6A RNA methyltransferase METTL3 as a retroelement suppressor forming immunostimulatory dsRNA in colorectal cancer. The authors used mainly two cell lines, HT29 and HCT116, which showed significant different effects of METTL3 downregulation (via shRNAs). **The METTL3 downregulation in HT29 led to higher amount of DEGs and dsRNA. A validation was performed with two additional cell lines. Overall, the authors identified the source of dsRNA as mainly SINE (>43 %) with Alu inverted repeats. However, LINE were found >31 % overexpressed, which would have been interesting to investigate further, also under the aspect, if SINEs were significantly higher expressed in respect to LINE (Suppl. Fig. 3) and did this justify to include Alu in the title?** The authors further showed that METTL3 loss elevated expression of ISGs. Mice experiments with HT29 and HCT116 plus/minus METTL3 knockdown (shRNA) plus/minus Decitabine (demethylation) led to different tumor formations.

Regarding the reviewer's comment on overexpressed LINEs, we fully agree that this is an important point. The role of differentially expressed LINEs following METTL3 knockdown indeed warrants further investigation. In this study, we focused primarily on SINEs and included Alu elements in the title because our RNase/MDA5 protection assay identified IR-Alus as the predominant source of immunostimulatory dsRNA sensed by MDA5 upon METTL3 depletion. Nevertheless, we acknowledge that the observed changes in LINE expression may reflect their involvement in additional regulatory pathways influenced by METTL3 knockdown, which we plan to investigate in future studies.

Although a well-rounded study with an employment against colorectal cancer, the author's results are somehow lessened in their novelty due to various prior publications (also cited as manuscript references), which I want to mention since some correlate with and confirm the results from Wang et al:

- **We thank the reviewer for this thoughtful comment. While prior studies have indeed explored related aspects, we believe our work provides several substantive advances beyond these earlier findings. To clarify the distinct contributions of our study, we address each cited manuscript in relation to our results below.**

1) METTL3 and m6A incorporation modulates A-to-I editing (ADAR) (Xiang et al. 2018Mol Cell)

- **We appreciate the reviewer's reference to Xiang et al., which reported a mutually exclusive relationship between m^{6}A modification and A-to-I editing, demonstrating that an adenosine carrying an m^{6}A mark cannot be edited by ADAR1, and vice versa. This study provides mechanistic insight into the interplay between these two RNA modifications. However, its focus remains on biochemical regulation rather than the downstream cellular or phenotypic consequences in cancer.**
  In contrast, our study does not examine a direct correlation between METTL3 and ADAR1 activity. Instead, we show that METTL3 depletion leads to an accumulation of immunostimulatory dsRNA that is sufficient to trigger a viral mimicry response despite the presence of ADAR1. Importantly, the central novelty of our work lies in

identifying differential sensitivity of colorectal cancer cells to METTL3 depletion, a phenomenon not previously described.
We therefore believe that while Xiang et al. offers important mechanistic context, it does not diminish the originality of our findings. Rather, it highlights an interesting, unresolved question, whether METTL3 and ADAR1 may coordinately regulate repeat element–derived dsRNAs in the context of cancer, which we agree remains an open avenue for future investigation.

2) M6A plays a role in development (see ref. #21)

- **This comment refers to the study by Gao et al., which we have also discussed in our manuscript. While both studies report that loss of m^{6}A can induce viral mimicry responses, there are important distinctions in biological context, methodology, and mechanistic conclusions.**

  Gao et al. focused on hematopoietic stem cells and, without directly mapping m^{6}A sites, relied primarily on public datasets restricted to protein-coding transcripts. Based on these data, they concluded that viral mimicry arises from dsRNAs derived mainly from protein-coding genes and a subset of lncRNAs (including MALAT1). <u>Their J2-RIP-seq approach captured global dsRNA accumulation but did not distinguish immunostimulatory from non-immunostimulatory species (i.e., RNAs capable of activating MDA5 versus those bound by other sensors).</u>
  In contrast, our study investigates colorectal cancer and demonstrates that the cellular response to METTL3 depletion is context-dependent, as not all CRC cells undergo viral mimicry. Using RNase/MDA5 protection sequencing, we show for the first time in the context of METTL3, that the immunostimulatory dsRNAs arise specifically from repeat elements. Moreover, our data indicate that once repeat elements are transcribed in cancer cells, they are further regulated at the post-transcriptional level by METTL3.
  Thus, while Gao et al. provide valuable insight into the role of m^{6}A in hematopoietic development, our study advances the field by demonstrating (i) that colorectal cancer cells exhibit differential sensitivity to METTL3 loss, and (ii) that viral mimicry in this setting is driven by repeat element–derived immunostimulatory dsRNAs, a distinct and previously uncharacterized mechanism not addressed by Gao et al.

3) M6A regulates other RE, like ERVs (#18)

- **We appreciate the reviewer highlighting the study by Chelmicki et al., which we also discuss in our manuscript. While both studies examine m^{6}A regulation of ERVs, the biological context and mechanisms differ. Chelmicki et al. used mouse embryonic stem cells and showed that METTL3/METTL14 restrict IAP ERVs mainly by reducing their mRNA stability through YTHDF readers.**
  In contrast, our work focuses on human colorectal cancer and demonstrates that METTL3 depletion induces viral mimicry in a context-dependent manner. Rather than acting through ERV transcript stability, we show that METTL3 regulates repeat element–derived immunostimulatory dsRNAs, which drive viral mimicry only in sensitive CRC cells. This mechanism is distinct from, and not addressed in, the Chelmicki et al. study.

4) METTL3 and IFN response, also with anti-PD1 (#22)

We thank the reviewer for highlighting the study by Guirguis et al., who showed that METTL3 inhibition leads to dsRNA accumulation and a cell-intrinsic interferon response that enhances antitumor immunity. While the overall outcome is similar to ours, their work did not investigate the origin or structural basis of the dsRNAs induced by METTL3 inhibition. In contrast, our study identifies intronic IR-Alus as the predominant source of immunostimulatory dsRNAs in colorectal cancer cells and demonstrates that these dsRNAs form or persist due to loss of m^6A, providing mechanistic insight into dsRNA biogenesis that was not addressed by Guirguis et al.

5) METTL3 m6A dsRNA and immunology (#38)

- **We thank the reviewer for raising this point. Xiao et al. reported that METTL3-mediated m^6A regulates mRNA stability and dsRNA levels to control γδ T-cell differentiation. While this study highlights an important immunological role for METTL3, it focuses on T-cell lineage specification and does not investigate the genomic origin, structural features, or immunostimulatory capacity of the accumulated dsRNAs.**
  In contrast, our study investigates METTL3 function in colorectal cancer and reveals a distinct mechanism in which METTL3 depletion triggers viral mimicry selectively in METTL3-sensitive CRC models. Importantly, we identify intronic IR-Alu elements as the predominant source of immunostimulatory dsRNAs, show that their accumulation occurs independently of transcriptional induction, and demonstrate that loss of m^6A promotes their formation or persistence. These mechanistic insights into dsRNA origin and m^6A-dependent regulation were not addressed by Xiao et al.

6) Inv repeat Alus (AIR) bind MDA5 (#32)

- **Ahmad et al. showed that IR-Alu dsRNAs can activate gain-of-function MDA5 mutants, breaking self-tolerance and driving inflammation. Their study therefore focuses on mutant MDA5–driven pathology. In contrast, we show that loss of METTL3 in colorectal cancer cells increases IR-Alu–derived immunostimulatory dsRNA, which is sufficient to activate wild-type MDA5. Thus, our work reveals a distinct mechanism in which METTL3 normally restrains the immunogenicity of IR-Alus in cancer.**

7) MDA5 induces a Z-RNA conformation of Alus for ADAR1 (Nichols et al. 2021, Nat Commun).

- **Nichols et al. showed that MDA5 can induce a Z-RNA conformation in Alu elements, facilitating ADAR1 editing and thereby maintaining self-tolerance. Their work focuses on how MDA5 and ADAR1 cooperate to prevent inappropriate innate immune activation.**
  In contrast, our study demonstrates that loss of METTL3 in colorectal cancer leads to the accumulation of immunostimulatory IR-Alu–derived dsRNAs, which activate wild-type MDA5. Rather than describing how MDA5 shapes ADAR1 editing, we reveal a distinct mechanism in which METTL3 normally suppresses the immunogenic potential of repeat element dsRNAs. Importantly, it remains unknown when and where ADAR1 edits IR-Alus relative to METTL3, and how IR-Alus may escape one RNA

modifier but be restricted by the other, highlighting an unresolved question for future studies.

8) Alu inv rep (AIR) is the most common source of dsRNA (#32)

- **Ahmad et al. showed that IR-Alu dsRNAs can activate gain-of-function MDA5 mutants, breaking self-tolerance and driving inflammation. Their study therefore focuses on mutant MDA5–driven pathology. In contrast, we show that loss of METTL3 in colorectal cancer cells increases IR-Alu–derived immunostimulatory dsRNA, which is sufficient to activate wild-type MDA5. Thus, our work reveals a distinct mechanism in which METTL3 normally restrains the immunogenicity of IR-Alus in cancer.**

9) METTL3 methylates ADAR1 (Tassinari et al. 2021 Genome Biol)

- **The study reporting ADAR1 as a downstream target of METTL3 in glioblastoma highlights an editing-independent, pro-oncogenic role of ADAR1 that is regulated by m^{6}A. Their work focuses on METTL3–ADAR1 interactions in glioblastoma biology, without examining dsRNA origin, immunostimulation, or viral mimicry.**

  In contrast, our study shows that in colorectal cancer, loss of METTL3 leads to the accumulation of immunostimulatory IR-Alu–derived dsRNAs, which activate wild-type MDA5 and drive viral mimicry in a context-dependent manner. We therefore uncover a mechanism centered on repeat element–derived dsRNA regulation, rather than on ADAR1's pro-tumor functions. Notably, the interplay between METTL3 and ADAR1 specifically on IR-Alu dsRNAs remains uncharacterized and represents an important open question.

10) METTL3 regulate tumor immune m6A (Zhu et al. 2023 Cell Oncol)

- **Zhu et al. showed that METTL3 promotes endogenous dsRNA-induced anti-tumor immunity in pancreatic cancer, but did not identify the source or mechanism generating these dsRNAs. In contrast, our study demonstrates that loss of METTL3 in colorectal cancer leads to the post-transcriptional accumulation of immunostimulatory IR-Alu dsRNAs, directly activating wild-type MDA5 in a context-dependent manner. This repeat element–based mechanism is unique to our work and not addressed by Zhu et al.**

11) METTL3 acts as an oncogene in colorectal cancer (Li et al. 2019, Mol Cancer)

- **We thank the reviewer for noting the study by Li et al., who reported that METTL3 functions as an oncogene in colorectal cancer by promoting m^{6}A-dependent stabilization of oncogenic transcripts. Their work focuses on proliferation-related m^{6}A targets and does not examine dsRNA biology, innate immune sensing, or variability in the cellular response to METTL3 loss.**

  In contrast, our study reveals that METTL3 depletion in colorectal cancer has context-dependent effects, with only a subset of CRC models undergoing viral mimicry. Using RNase/MDA5 protection sequencing, we identify repeat element–derived IR-Alu dsRNAs as the immunostimulatory species that accumulate upon

METTL3 loss, an RNA source and mechanism not addressed by Li et al. <u>Thus, while Li et al. describe oncogenic m^{6}A pathways downstream of METTL3, our work uncovers a distinct, immunity-driven mechanism that directly links METTL3 to the regulation of endogenous dsRNA and innate immune activation in CRC.</u>

12) PKR binds AIR (Kim et al. 2014, Genes & Dev)

- **Kim et al. demonstrated that PKR can bind AIR dsRNAs under stress conditions, linking IR-Alu structures to translational control via PKR activation. Their study focuses on PKR–AIR interactions in the context of cellular stress and does not examine how these IR-Alus are regulated or stabilized in cancer.**

  In contrast, our work shows that METTL3 loss drives the post-transcriptional accumulation of immunostimulatory IR-Alu dsRNAs in colorectal cancer. These RNAs activate wild-type MDA5 to induce interferon and, importantly, can also engage PKR, which triggers translational reprogramming and contributes to cancer cell death. <u>Thus, while Kim et al. established that IR-Alus can bind PKR, our study reveals why IR-Alus become immunogenic in cancer and demonstrates that their accumulation following METTL3 loss enables parallel sensing by distinct RNA sensors with different downstream outcomes.</u>

13) Inhibition of DNMT (DNMTi) induces SINE (#9)

- **DNMTi are known to activate endogenous SINE elements (Mehdipour et al, Nature, 2020), as shown in seminal studies by Roulois et al. (Cell 2015) and Chiappinelli et al. (Cell 2015), where DNMTi-induced SINE/ERV transcription triggers viral mimicry and type I IFN responses. These studies formed the rationale for combining DNMTi with METTL3 inhibition in our work. Specifically, because a subset of colorectal cancer cells is insensitive to METTL3 loss alone, we sought to pharmacologically increase endogenous dsRNA levels using DNMTi to sensitize these resistant models.**

  In contrast to DNMTi, which transcriptionally upregulate SINEs, our study shows that METTL3 loss causes post-transcriptional accumulation of immunostimulatory IR-Alu dsRNAs, driving viral mimicry only in sensitive CRC cells. Combining DNMTi with METTL3 inhibition therefore represents a targeted strategy to raise dsRNA to a lethal threshold of type I IFN signaling in otherwise resistant cells,obviating the need for an extensive drug screen and directly leveraging the mechanistic insights from both DNMTi-induced and METTL3-regulated dsRNA pathways.

Specific remarks and questions:

1) **Ln 121: which HCT116 cells were used, as there are many different available strains at ATCC?**
   - **We thank the reviewer for highlighting this point. We have now included the exact strain designations for all cell lines, including HCT116 (CCL-247™), as obtained from ATCC, in the revised Materials and Methods section.**

2) Ln 121 and ln 177, Table 2: please combine the cell lines with the CMS in Table 2, especially considering the RAS mutations (HCT116: KRASG13D; NCI-H716 and COLO201 RAS wt etc.)

- **We thank the reviewer for the suggestion. In the revised manuscript, we have added CMS classification for all colorectal cancer cell lines included in DepMap to Supplementary Table 6.**

3) Ln 274-279: The reference #32 was used for the RNase/MDA5 protection assay, although the reference #32 also claims that MDA5 wt has only limited binding capabilities of Alu-Alu hybrids (dsRNA), due to mismatches, bulges and A-to-I; in contrast to mutated MDA5. This could be a limitation of the Alu conclusions; please discuss.

- **We apologise for the ambiguity of this statement. Regarding the reviewer's comment that "MDA5 WT has only limited binding capabilities of Alu–Alu hybrids (dsRNA) due to mismatches, bulges, and A-to-I editing, in contrast to mutated MDA5", we cited Reference 32 as it was the first to develop and use the RNase/MDA5 protection assay. However, we agree with the reviewer's point and, for clarification, have now also cited our previous study (Mehdipour et al., 2020), in which we used an MDA5-protection assay with WT MDA5 to assess dsRNA species enriched after epigenetic derepression in colorectal cancer cells. Our findings showed an increase in Alu elements, indicating that these are the major species of endogenous dsRNA induced by epigenetic therapy. <u>Indeed, upon accumulation of endogenous dsRNAs, WT MDA5 can sense these species in a manner similar to gain-of-function MDA5</u>. In the current work, we further show that loss of m^6A leads to the accumulation of already transcribed or newly expressed repeat elements that act as immunostimulatory dsRNAs and can also be sensed by WT MDA5. We apologise that this was not sufficiently clear in the original manuscript and have revised the text accordingly.**

4) Ln 291 and Fig. 4F: generally, IR-Alus were found; were any of the Alu subfamilies (AluY, AluJ, AluS) dominant?

- **We thank the reviewer for this question. In HT29 and COLO201 METTL3-sensitive cells (Figure 3D-G; Supplementary Fig. 4E), the majority of upregulated Alus belong to the AluS and Alu J subfamilies. Odds ratio relative to Alu subfamilies present in the human genome (Supplementary Fig. 4F).**

  Similarly, in Figure 5E, both baseline and $METTL3^{KD}$-induced immunostimulatory RNAs are predominantly AluS (71.28% and 70.28%, respectively), each showing significant enrichment compared with the genomic proportion of genome-wide IR-Alus from this subfamily (odds ratio = 1.2, P < 0.0001) (Supplementary Figure 6C).

Figure 1

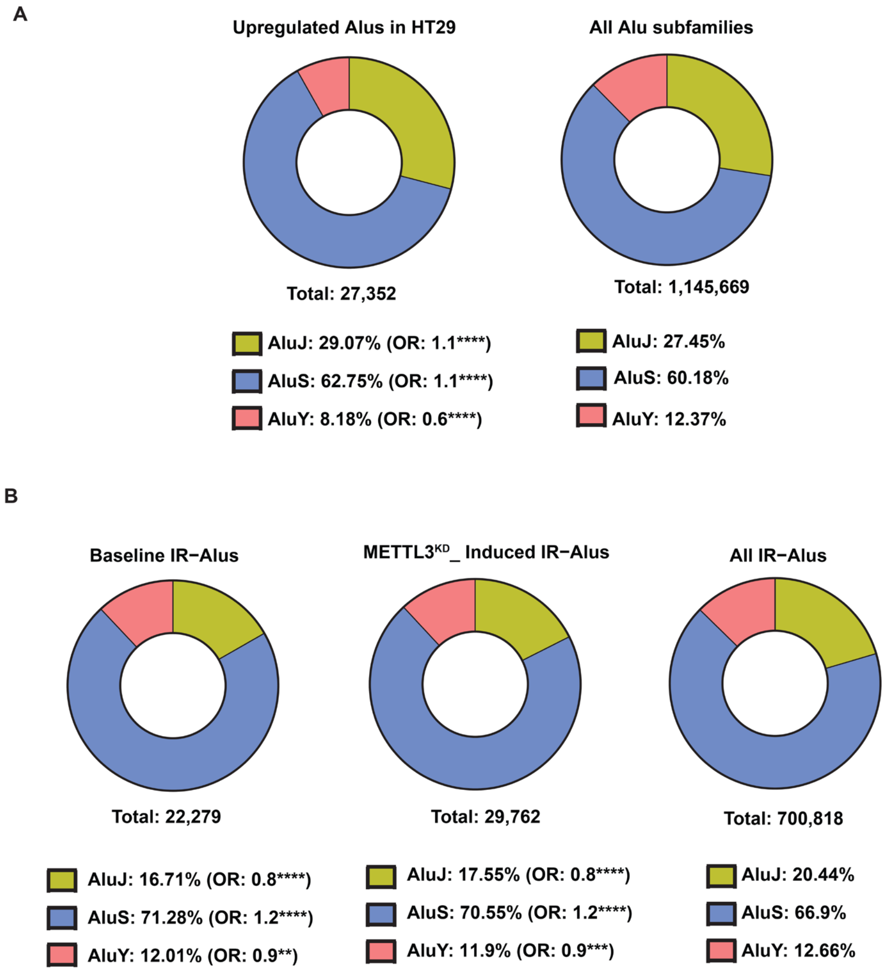

Figure 1. Alu subfamily distribution.

(A) Distribution of Alu subfamilies upregulated in HT29 CRC cells (n = 27,352 Alus), and all Alus present in the human genome (n = 1,145,669 Alus). Odds ratio shows depletion or enrichment for each Alu subfamily (AluS, AluJ and AluY) at baseline HT29 compared to Alu subfamily distribution of all Alus present in the human genome. ****P < 0.0001, two-sided Fisher exact test.

(B) Distribution of Alu subfamilies from baseline immunogenic IR-Alus (n = 22,279 IR Alus), METTL3^{KD}-induced immunogenic IR-Alus (n = 29,762 IR Alus), and IR-Alus present in the human genome (n = 700,818 IR Alus). Odds ratio shows depletion or enrichment for each Alu subfamily (AluS, AluJ and AluY) at baseline immunogenic IR-Alus or METTL3^{KD}-induced immunogenic IR-Alus compared to Alu subfamily distribution of all IR-Alus present in the human genome. ****P < 0.0001, two-sided Fisher exact test.

- **The newly generated data have now been incorporated into the revised manuscript as Supplementary Figures 4E, F and Supplementary Figure 6C.**

5) Ln 344 and 678: Why were different amounts of HT29 (500,000 cells) and HCT116 (250,000 cells) for NSG mice injections used?

- **The different numbers of injected cells reflect the distinct *in vivo* growth kinetics of the two CRC models. HT29 cells grow more slowly and have lower tumour-take rates in NSG mice, whereas HCT116 cells are more aggressive and engraft efficiently.**

Accordingly, we used higher numbers of HT29 cells (5 × 10^5) and lower numbers of HCT116 cells (2.5 × 10^5) to obtain comparable and measurable tumour growth within the experimental timeframe. These conditions were based on prior optimisation and are consistent with published xenograft studies; for example, an orthotopic NSG model required 1 × 10^6 HT29 cells but only 1 × 10^5 HCT116 cells [1].

Importantly, our intention was not to compare tumour growth between the two cell lines. METTL3 knockdown effects were assessed within each model independently, each with appropriate non-transduced and TGFP-transduced controls. Therefore, the differences in injected cell numbers do not influence the interpretation of METTL3-dependent effects.

6) Ln 348 and Fig. 9: How many cells were injected with METTL3KD1 etc. Explain METTL3KD1/4 (KD1-4?), as Fig. 1 has KD1-4 and Fig. 6 has only METTL3KD1.

- As shown in the revised Figure 8A, equal numbers of cells were injected into NSG mice for each HT29 group: 5 × 10^5 HT29 non-transduced (NT), 5 × 10^5 HT29 control (TGFP-transduced), 5 × 10^5 HT29 $METTL3^{KD1}$, and 5 × 10^5 HT29 $METTL3^{KD4}$ cells.

  All four shRNAs targeting METTL3 (KD1–KD4), shown in Supplementary Figure 1A, specifically target METTL3, and their sequences are listed in Supplementary Table 3. As shown in Supplementary Figure 1A, all four shRNAs efficiently reduced METTL3 protein levels in both HT29 and HCT116 cells. To ensure robust and reproducible results while minimizing potential off-target effects, we selected $METTL3^{KD1}$ and $METTL3^{KD4}$ for all downstream experiments. These included METTL3 knockdown in additional CRC models (COLO201, NCI-H716, POP92, and CSC73), as well as the *in vivo* studies.

  To improve clarity, we have reorganized the presentation of these data: the KD1–KD4 shRNA sequences and validation results have been moved to Supplementary Figure 1A and Supplementary Table 3, and we now explicitly state that all functional assays throughout the manuscript were performed using KD1 and KD4 across all CRC cell lines and PDX samples.

7) Ln 380: Please explain "accumulation of dsRNA, rather than increased mRNA stability" in more detail. Is this really an exclusion rule? Which proof do the authors have for either or?

- In this context, "mRNA stability" refers specifically to the stability of interferon-stimulated gene (ISG) transcripts. Our data indicate that ISG induction following METTL3 depletion is primarily driven by sensing of accumulated endogenous dsRNA rather than by altered ISG transcript stability. Supporting this interpretation, disruption of the dsRNA-sensing pathway through MAVS depletion almost completely abolishes ISG induction (Figure 2H), indicating that any contribution of ISG mRNA stability is minimal.

Our CUT&RUN analysis further supports a transcriptional activation mechanism: METTL3^{KD} increases H3K4me3 occupancy at ISG promoters (Figure 5A and Supplementary Figure 5D), consistent with enhanced upstream signaling triggered by dsRNA accumulation. To directly assess whether METTL3 loss affects RNA stability, we performed Actinomycin D chase assays for MX1 (as a representative ISG transcript) and for indicated repeat-derived RNAs enriched in our RNase/MDA5 protection assay. These analyses revealed no major differences in RNA half-life upon METTL3 depletion, as shown below and in the revised Supplementary Fig. 7A–D, indicating that altered transcript stability is unlikely to account for the observed upregulation of ISGs or the assessed dsRNAs.

Multiple orthogonal assays demonstrate that METTL3 loss leads to an increase in immunostimulatory dsRNAs. J2 immunofluorescence and J2 dot blot analyses revealed a clear accumulation of dsRNAs (>40 bp) in METTL3-depleted, IFN-responsive CRC cells. Our immunoprecipitation experiments (Figure 6A, B) and the reduced m^{6}A scores around MDA5-protected regions (Figure 6C) further reinforce this conclusion: METTL3^{KD} decreases m^{6}A levels specifically within the J2-IP fraction, indicating that the dsRNAs recognized by J2 become relatively hypomethylated upon METTL3 depletion. Importantly, this reduction in m^{6}A coincides with increased recovery of the same transcripts in J2-IP, demonstrating that METTL3 loss alters both the methylation state and the abundance/accessibility of J2-reactive dsRNAs rather than simply reducing total RNA levels.

Although RNAfold predictions did not reveal substantial differences in global minimum-free-energy structures between methylated and unmethylated sequences (an example is shown below), this is expected because m^{6}A effects are predominantly local and kinetic, rather than global. Extensive biochemical and structural studies have shown that m^{6}A disrupts base stacking, weakens local duplex stability, and increases RNA structural flexibility [2-4]. Loss of m^{6}A may therefore allow repeat-derived transcripts to adopt or persist in more stable double-stranded conformations that are normally disfavored when methylated, an interpretation supported by our J2 immunofluorescence, dot blot, and immunoprecipitation data (Figures 1G, 6A–C, Supplementary Figure 2B).

Together, these results support a model in which METTL3-mediated m^{6}A normally restrains the formation or persistence of immunostimulatory dsRNAs derived from repetitive elements. METTL3 depletion reduces m^{6}A on these transcripts, facilitating the accumulation of unmethylated dsRNAs that activate the MAVS-dependent innate immune response.

Figure 2

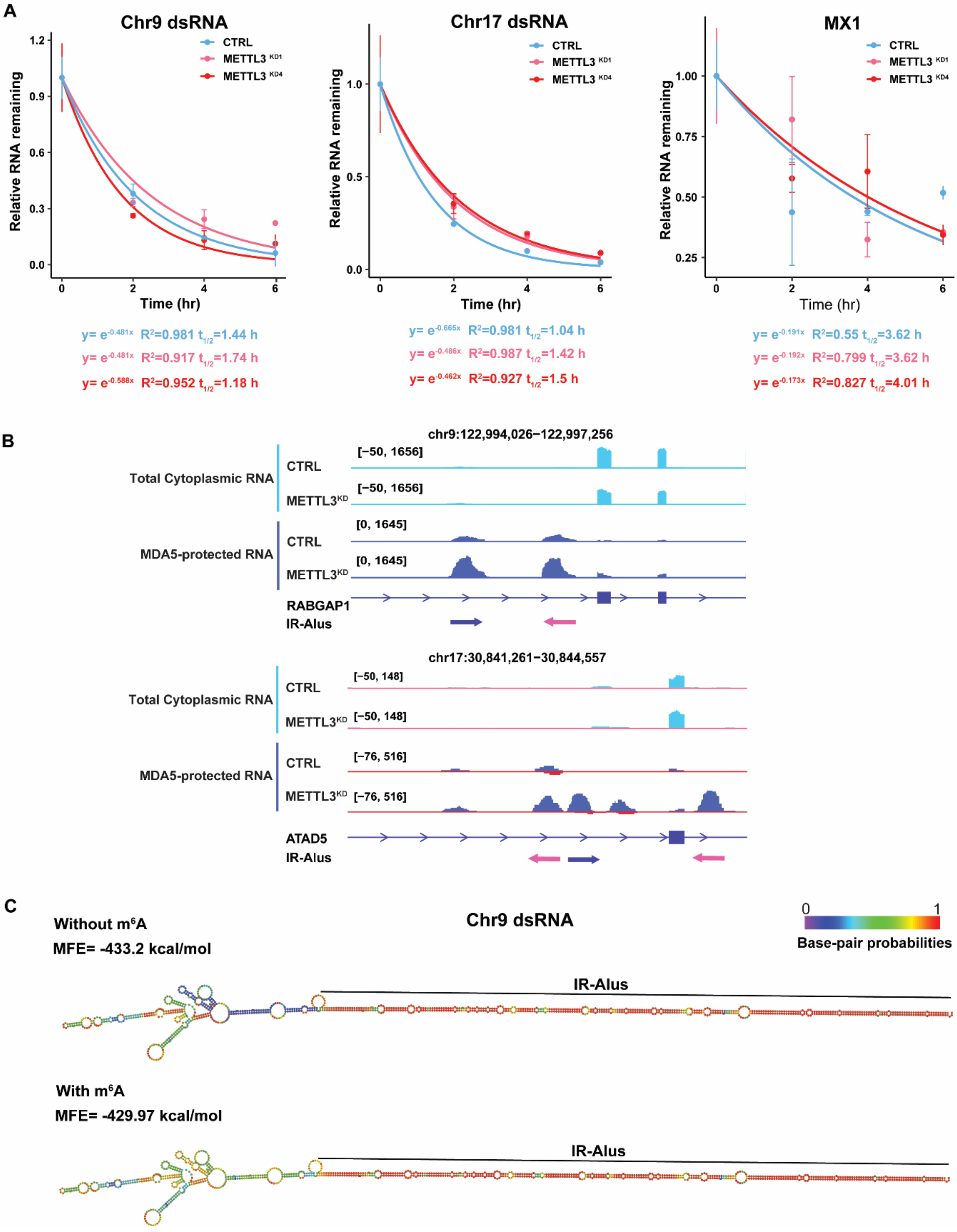

Figure 2. METTL3 depletion increases hypomethylated IR-Alu–derived dsRNAs without altering RNA stability in HT29 CRC cells.

(A) HT29 control and METTL3^{KD} cells were treated with actinomycin D (1 μg/mL), and RNA was extracted at the indicated time points for qPCR analysis. The degradation rate of each transcript was fitted using $y = e^{-}$

$^{k(decay)x}$. Fits with $R^2 \geq 0.95$ were considered robust; RNA half-lives were calculated as $t_1/_2 = ln(2) / k(decay)$. $n = 2$ technical replicates.

(B) Representative genomic tracks showing METTL3^{KD}-induced immunostimulatory inverted-repeat Alu (IR-Alu) elements highlighted in pink and blue. The top two tracks display total cytoplasmic RNA with sense (sky blue) and antisense (pink) reads distinguished. The next two tracks show RNase A–digested cytoplasmic RNA, with sense (dark blue) and antisense (red) reads indicated.

(C) Representative examples of predicted dsRNA structures formed by IR-Alu pairs, shown either in their unmethylated form or with all adenosines in m^{6}A motif (DRACH) substituted to mimic m^{6}A-modified positions. RNAfold-predicted secondary structures and corresponding minimum free energy (MFE)are shown for an IR-Alu locus on chromosome 9.

8) Ln 419: Decitabine is a new avenue for treatment, although other inhibitors (Ln 418) were not tested here. This is a too strong conclusion and judgement.

- **We agree with the reviewer and appreciate the feedback. We have rephrased the statement in the revised manuscript as follows: "To overcome this challenge and extend the therapeutic potential of METTL3 inhibitors for the treatment of solid tumours, our findings provide a rationale for combining METTL3 targeting with DNMT inhibitors and, potentially, other compounds that have been shown to modulate the levels of immunostimulatory dsRNAs, such as CDK4/6 inhibitors, LSD1, SETDB1, G9a, and XRN1. However, the therapeutic potential of these additional combinations needs to be further explored and experimentally validated."**

9) Ln 492: Was DNase I used for RNA isolations?

- **DNase I treatment was used during RNA isolation, and this has now been added to the revised Materials and Methods section as follows: "Total RNA was extracted with the RNeasy Mini Kit following the manufacturer's protocol. Briefly, after adding RLT buffer and 70% ethanol to the cell pellets, in-column DNase I digestion was performed using the QIAGEN RNase-Free DNase Set (catalogue 79256)."**

10) Ln 500: It is a general standard that for qPCR two reference genes have to be used (not only GAPDH), esp. due to the fact that GAPDH concentrations vary between individuals, development, cell cycle etc. (Bustin SA 2000, J Mol Endocrinol).

- **We thank the reviewer for this helpful suggestion. To exclude the possibility that the observed gene expression changes were due to effects of METTL3 depletion or DNMTi treatment on GAPDH, we repeated selected qPCR analyses using three housekeeping genes (GAPDH, RPLP0, and β-actin (ACTB)). While the magnitude of fold changes for MX1, ISG15, and METTL3 varied slightly depending on the reference gene, the overall trends were consistent across all normalizers, indicating that the results are not attributable to changes in housekeeping gene expression.**

Figure 3

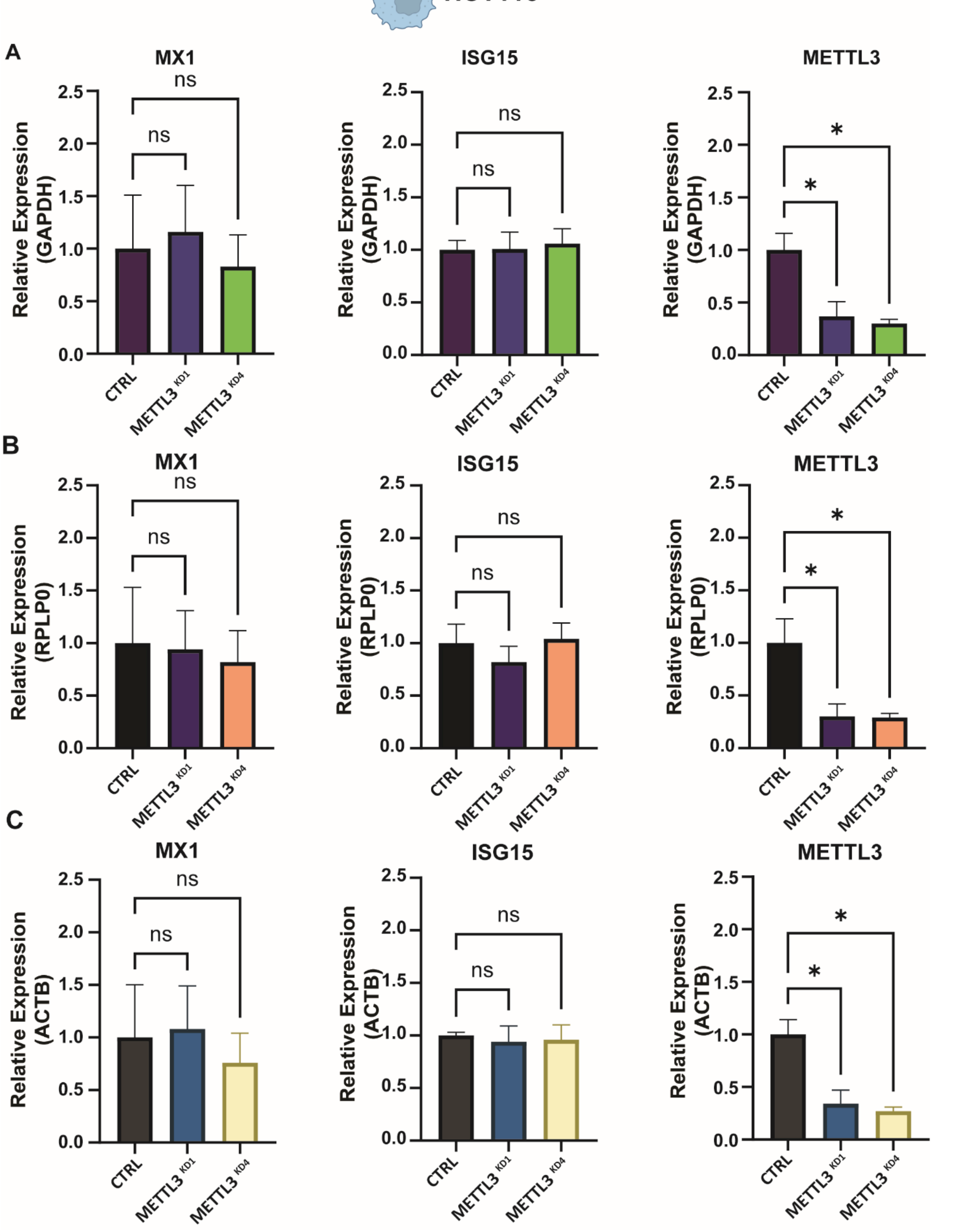

Figure 3. Comparison of relative mRNA expression levels normalized to various housekeeping genes. Quantitative PCR (qPCR) analysis of MX1, ISG15, and METTL3 expression in HCT116 cells relative to the control (CTRL) sample, normalized to **(A)** GAPDH, **(B)** RPLP0, and **(C)** β-actin (ACTB). Data are presented as mean ± SD. Statistical significance was determined using one-way ANOVA with Dunnett's correction (*$P \leq 0.05$, **$P \leq 0.01$, ***$P \leq 0.001$, ****$P < 0.0001$).

Figure 4

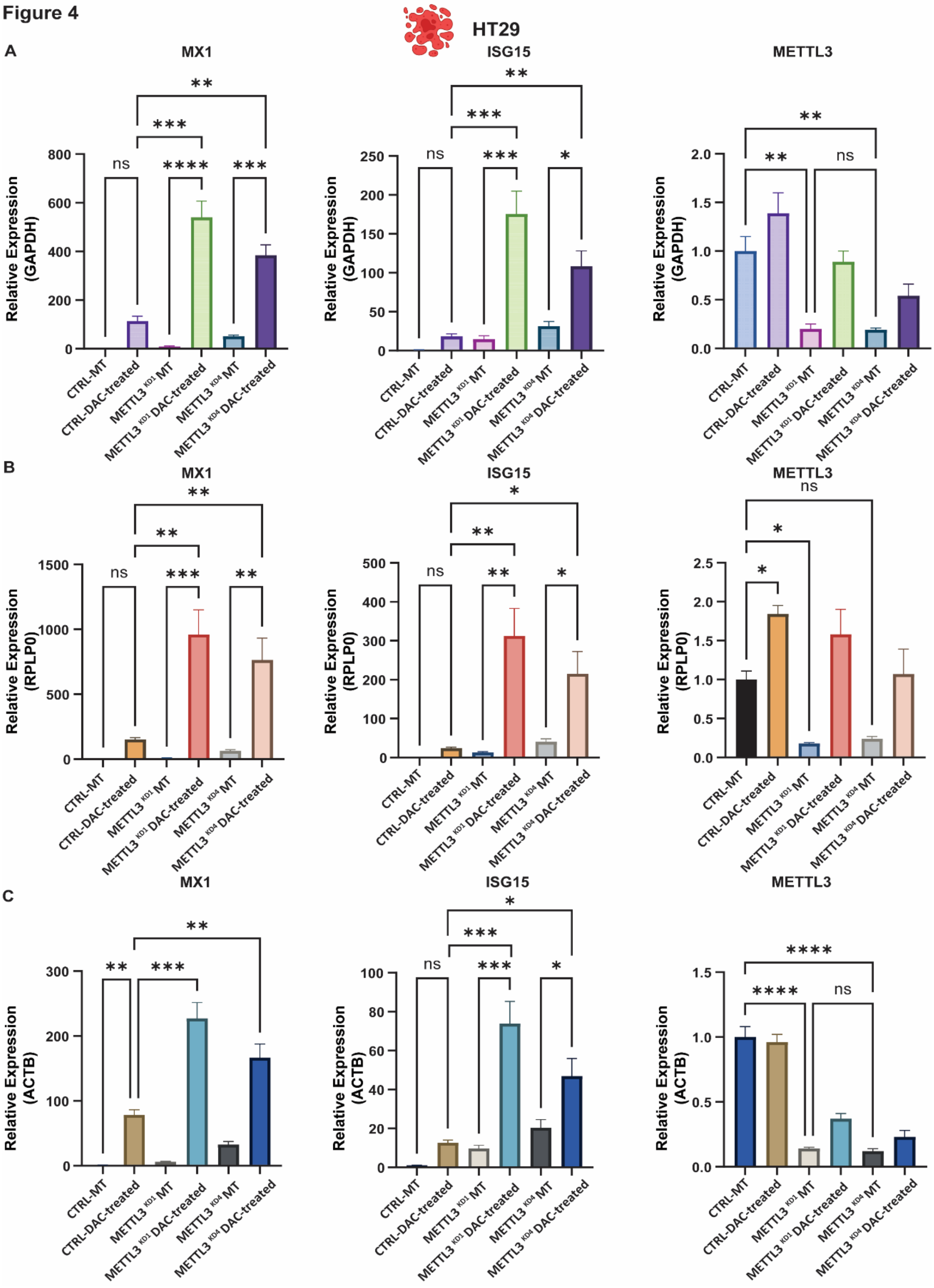

Figure 4. Comparison of relative mRNA expression levels normalized to various housekeeping genes. Quantitative PCR (qPCR) analysis of MX1, ISG15, and METTL3 expression in HT29 colorectal cancer (CRC) cells relative to the control (CTRL) sample. Cells were either wild type (WT) or METTL3 knockdown (KD), and were mock-treated (MT) or treated with the DNA methyltransferase inhibitor decitabine (DAC). Expression levels were normalized to **(A)** GAPDH, **(B)** RPLP0, or **(C)** β-actin (ACTB). Data are presented as mean ± SD. Statistical significance was determined using one-way ANOVA with Dunnett's correction (*P ≤ 0.05, **P ≤ 0.01, ***P ≤ 0.001, ****P < 0.0001).

Reviewer #2 (Remarks to the Author):

In this manuscript submission to Nature Communications, Wang, Y et al., present compelling data supporting a promoting role of the METTL3 gene, often elevated in cancer, in suppressing cell-intrinsic antiviral responses against transposable element-associated double-stranded RNA in colorectal cancer. This work has important implications for therapeutically enhancing the pro-apoptotic responses against transposable elements and double-stranded RNA in this cancer, especially in light of recent development and clinical trials of METTL3 inhibitors in solid . The authors demonstrate that sensitivity to inhibition of METTL3 is variable in human colorectal cancer cell lines and dependent on basal levels of retroelement expression, but that even in those lines that are insensitive to inhibition, synergy with METTL3 inhibition can be achieved by cotreatment with a DNA methyltransferase inhibitor (Decitabine) that also increases retroelement expression. These findings, if further validated, are both timely and potentially clinically impactful.

However, there are two major issues with the manuscript that currently temper enthusiasm: 1) most of the mechanistic work is performed in only one METTL3 knockdown sensitive (HT29) and insensitive (HCT116) colorectal cancer cell line. These findings should be validated in additional colorectal cancer cell lines to warrant publication in a rigorous journal such as Nature Communications. 2) The authors fail to cite critical prior research in the area of METTL3's role in modifying mRNA transcripts and viral mimicry, including highly relevant publications that have explored this concept specifically in CRC. It is therefore challenging to accurately evaluate the novelty of some of the key findings.

Which findings are corroborative of prior studies of METLL3 in CRC, which findings are derivative of known roles of METTL3 in other contexts (i.e., similar findings but not specifically in CRC), and which findings are novel? A nuanced discussion of the above questions, inclusive of relevant citations, should be provided throughout the Introduction, Results section, and in the Discussion.

Specific comments are included below:

1)

114-116: "This analysis revealed that METTL3 expression is elevated in cancerous tissues compared to corresponding normal tissues in the majority of cancers, including colorectal adenocarcinoma (COAD)."

It is essential to cite prior literature here, as this has previously been reported in different cancers, including CRC (e.g., Pan, J, et al., Journal of Experimental and Clinical Cancer Research, 2022; Tang, T, et al., Cellular Signaling, 2022; etc.).

- **These citations have been added to acknowledge prior studies reporting elevated METTL3 expression in CRC and other cancers.**

2)

124-125: "Collectively, these findings suggest that CRC cells exhibit varying vulnerabilities to METTL3 depletion."

It is also important to cite prior literature here, as previous reports exist of antitumor effects of knockout and knockdown of METTL3 in CRC, including in the HCT116 line used in the current study (e.g., Pan, J, et al., Journal of Experimental and Clinical Cancer Research, 2022; Yang, Z, et al., Signal Transduction and Targeted Therapy, 2021; etc.).

- We thank the reviewer for this helpful suggestion. These citations have been added to acknowledge prior studies reporting the role of METTL3 in CRC. The revised text now reads as follows:

"Although CRC cells have been shown to respond to METTL3 targeting [5, 6], our findings uncover an additional layer of complexity: CRC models vary substantially in their vulnerability to METTL3 inhibition."

This revision acknowledges prior work while emphasizing the novelty of our findings.

3)

Can the authors reconcile the results of the previous studies reporting a decrease in viability in HCT116 with targeting METLL3 with their own results showing no effect (Figure 1C)? Could this perhaps be due to poor knockdown efficiency in HCT116? KD3 and KD4 are most potent in HT29 and also achieve much deeper RNA knockdown than in HCT116, so it is possible that more complete knockdown (or knockout) would have a phenotype in HCT116, as previously reported. CRISPR-Cas9 (or other) knockout (as opposed to knockdown) strategies should be considered.

Given the above, this line in the introduction is an overstatement and not accurate: 98-99: "However, the vulnerability of CRC to METTL3 targeting and the underlying mechanisms remain unexplored."

Specifically, there are multiple published studies exploring the vulnerability of CRC to METTL3 targeting, and it is quite clear that METTL3 levels are elevated in CRC and knockout/knockdown reduces CRC viability in vitro and in vivo. However, the mechanisms underlying this vulnerability, particularly as relate to RE and antiviral responses as proposed in this study, remain incompletely understood. The current study, therefore, has novelty, but should not overstate the novelty of the above central observations or ignore the important literature already published in this space.

- We thank the reviewer for these constructive comments.
  Regarding the first point, although several studies have reported reduced viability of CRC cell lines, including HCT116, following METTL3 depletion [5-7], the magnitude of these effects varies and does not fully align with DepMap data, which likewise demonstrates heterogeneous METTL3 dependency across CRC models. These observations indicate that METTL3 dependency is context-specific rather than uniform.
  Furthermore, the prior reports describing METTL3 targeting in HCT116 did not include RNA-seq analyses comparing METTL3-wild-type and METTL3-deficient cells generated by shRNA or siRNA knockdown [5, 7, 8]. Consequently, the transcriptomic consequences of METTL3 loss in this model remain insufficiently characterized. Moreover, Shen et al. evaluated METTL3 depletion in HCT116 using only a single

isogenic clone, thereby limiting the robustness and generalizability of their conclusions. These limitations underscore the need for our systematic, orthogonal analyses and support our interpretation that METTL3 dependency and its downstream effects are more heterogeneous than previously appreciated.

All four shRNAs we initially tested reduced METTL3 protein levels (Supplementary Figure 1A), and KD4 showed slightly stronger effects in viability assays in HT29 cells (Figure 1D). To minimize off-target effects, we used two independent shRNAs (KD1 and KD4) for all downstream assays. RNA-seq with both shRNAs in HCT116 confirmed highly concordant transcriptional changes.

Complementary assays (Western blot, colony formation and CellTiter-Glo) in CRISPR–Cas9 knockout model using one HCT116^{KO} clone showed only modest viability effects in HCT116, indicating intrinsically lower METTL3 dependency rather than incomplete knockdown.
The discrepancy with some published studies likely reflects differences in depletion kinetics, and subclonal or culture-condition variability, all of which can shift the threshold for IFN-driven cytotoxicity.

- Regarding the second point, we have now cited relevant studies showing METTL3 upregulation and revised the introduction to: <u>"However, the differential vulnerability of CRC cells to METTL3 targeting and the mechanisms underlying their varying dependency on METTL3 remain incompletely understood."</u>

Figure 5

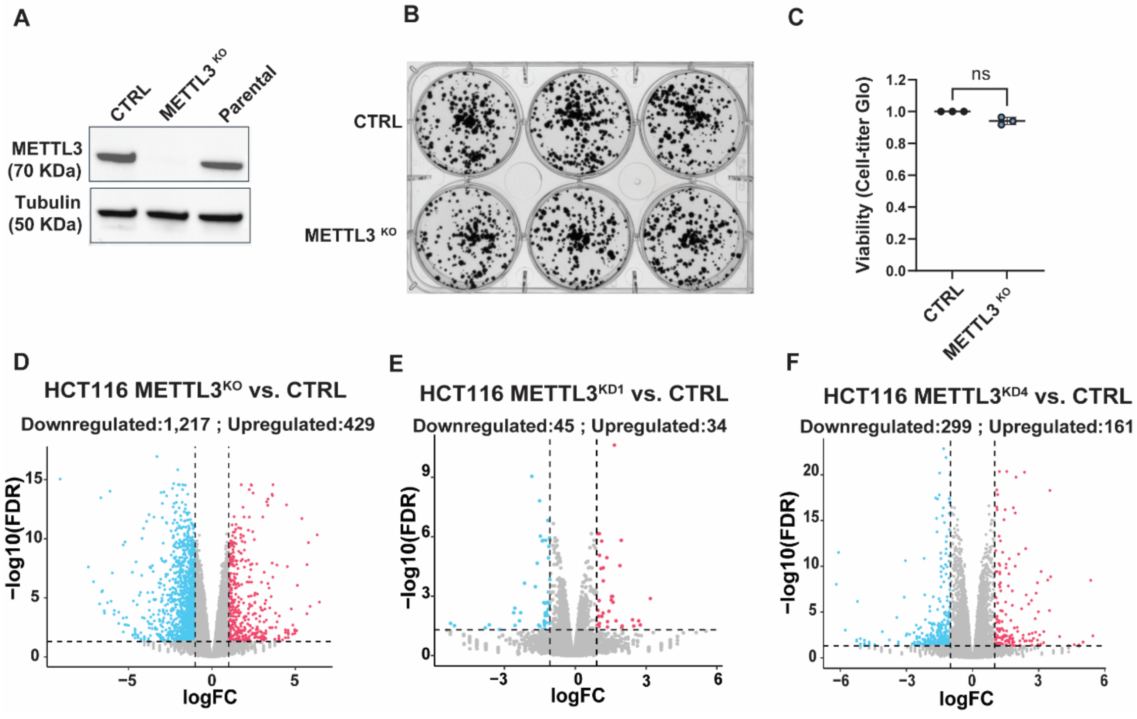

Figure5. Effects of METTL3^{KO} on viability, colony formation, and transcriptional changes in HCT116 cells.
(A) Immunoblot analysis of METTL3 knockout (^{KO}) efficiency in HCT116 CRC isoclone cell lines generated using non-targeting control sgRNA (CTRL) or METTL3 sgRNA. A representative blot shows METTL3 protein levels, with α-tubulin used as a loading control.
(B) Colony-forming ability of HCT116 cells transfected with CTRL or METTL3^{KO} sgRNA, visualized by crystal violet staining.
(C) Viability of CTRL and METTL3^{KO} HCT116 CRC cells measured by the CellTiter-Glo luminescence assay. Data are presented as mean ± SD (n = 3 biological replicates). ns: not significant (P > 0.05), Mann–Whitney test.
(D–F) Volcano plots showing differential gene expression in METTL3^{KO} compared with CTRL **(D),** METTL3^{KD1} compared with CTRL **(E),** and METTL3^{KD4} compared with CTRL **(F)** in HCT116 cells. Red: upregulated; gray: not significant; blue: downregulated. Significance was defined as FDR < 0.05 and $|\log_2 FC| \geq 1$. Data represent two independent experiments.

4)

111: "Colorectal cancer cells show different sensitivity to METTL3 targeting" Once again, the results presented in this section fail to cite the prior literature, which supports the same conclusions through similar analyses.

- **We have now added the relevant citations to acknowledge prior studies reporting the role of METTL3 in CRC. The revised text has been updated accordingly to better reflect existing knowledge while clarifying our contribution.**
"Although CRC cells have been shown to respond to METTL3 targeting [5, 6], our findings uncover an additional layer of complexity: CRC models vary substantially in their vulnerability to METTL3 inhibition."

5)

228-229: "METTL3 loss drives ISG activation in m6A-high CRC cells via transcriptional regulation"

Can these results be validated in the other lines (COLO201, NCI-H716, POP92, CSC73)? Given that basal levels of RE expression is being proposed as a key determinant of sensitivity to METLL3 KO in CRC, it is important to validate this in more than n = 1 cell line.

- **We thank the reviewer for this valuable suggestion. In line with this recommendation, we have already assessed and validated the effects of METTL3 targeting on ISG15 and MX1 expression by qPCR (Supplementary Figure 1I,J). In addition, we evaluated basal dsRNA levels using J2 antibody staining and measured global m^{6}A levels across all CRC models (Figure 6D).**

  To further strengthen our analysis, we performed RNA sequencing on four CRC cell lines and one patient-derived xenograft (PDX) CRC model representing the spectrum of METTL3 sensitivity: the METTL3-insensitive CSC73 PDX, NCI-H716 and HCT116 CRC cell lines, and the METTL3-sensitive HT29 and COLO201 cell lines. POP92 PDX CRC cells, which are METTL3-sensitive, exhibited extremely low RNA integrity

numbers (RINs) following METTL3 knockdown (as shown below); therefore, only POP92 control cells were sequenced.

These new datasets have been incorporated into the revised manuscript as Supplementary Figure 1E and Supplementary Figure 4. Collectively, they further reinforce our conclusion that basal dsRNA abundance and global m^6A levels correlate with the heterogeneous sensitivity of CRC models to METTL3 depletion.

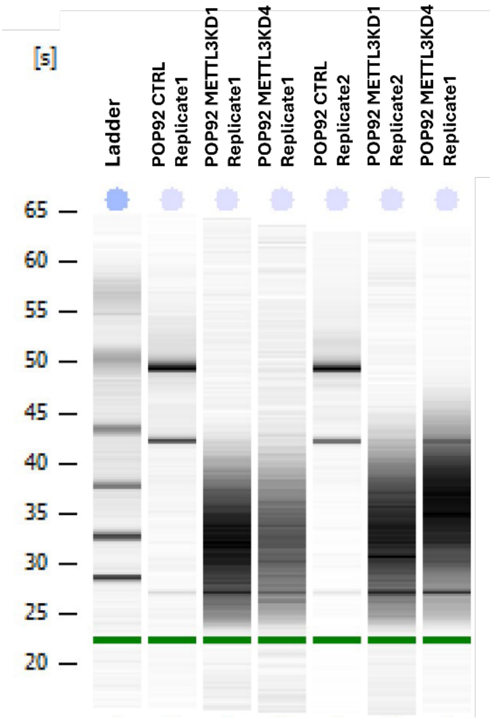

Figure 6. Bioanalyzer analysis of RNA integrity in POP92 PDX CRC cells following METTL3 knockdown (KD). Bioanalyzer traces showing RNA quality from POP92 PDX CRC samples. Lane 1: RNA ladder. Lane 2: POP92 CTRL, biological replicate 1 (RIN = 8.9). Lane 3: POP92 $METTL3^{KD1}$, biological replicate 1 (RIN = 1.9). Lane 4: POP92 $METTL3^{KD4}$, biological replicate 1 (RIN = 2.2). Lane 5: POP92 CTRL, biological replicate 2 (RIN = 9.8). Lane 6: POP92 $METTL3^{KD1}$, biological replicate 2 (RIN = 1.9). Lane 7: POP92 $METTL3^{KD4}$, biological replicate 2 (RIN = 2.2).

6)

223-226: "Moreover, basal RE expression may serve as a predictive signature for determining cancer cell sensitivity to METTL3 inhibition. This novel insight could provide new avenues for identifying CRCs more likely to respond to METTL3 inhibition based on their baseline RE expression profiles."

Given that there is only one sensitive and one insensitive cell line evaluated in this analysis (Figure 4F), it is impossible to assess this hypothesis with any statistical certainty. Additional cell lines must be included to be able to make this statement with any confidence—must have at least n = 3 sensitive and n = 3 insensitive, which the authors have already identified with the additional lines shown in Figure 3.

- We thank the reviewer for this valuable suggestion. In response, we expanded our analysis to include basal dsRNA levels (J2 staining) and global m^6A measurements

across all CRC models (Figure 6D). We also performed RNA-seq on four CRC cell lines, including CSC73 and POP92 PDX models. These new datasets have been incorporated into the revised manuscript (Figure 3D–G and Supplementary Figure 3B) and further support our conclusion that differential sensitivity to METTL3 loss is linked to m^6A levels and dsRNA-forming capacity rather than total retroelement expression. Notably, we find no direct correlation between RE expression, RE-derived dsRNA levels, and basal ISG expression (Supplementary Fig. 1K-M).

Based on these expanded analyses, we propose that CRC vulnerability to METTL3 inhibition depends on the combined status of global m^6A deposition and the capacity of RE-derived transcripts to form dsRNA. CRC models with high m^6A levels harbor a large pool of methylated IR-Alu–containing transcripts that are normally kept in a non-dsRNA state; however, upon METTL3 loss, permissive for folding into immunostimulatory dsRNA, thereby triggering viral mimicry. In contrast, CRC models with low basal m^6A deposition and limited dsRNA-forming competence remain resistant, even when RE expression is high. Thus, high m^6A levels in METTL3-proficient (wild-type) cells confer susceptibility not because m^6A promotes dsRNA formation, but because loss of m^6A releases IR-Alu transcripts to fold into dsRNA.

7)

228-229: "METTL3 loss drives ISG activation in m6A-high CRC cells via transcriptional regulation"

Again, for any meaningful conclusions to be drawn from this section, additional cell lines must be included and statistical tests performed.

- We thank the reviewer for this valuable suggestion. We have already assessed and validated the effect of METTL3 targeting on ISG15 and MX1 expression by qPCR in all the 6 CRC cells (Supplementary Figure 1I, J). Additionally, we examined through RNA-sequencing the ISG activation in NCI-H716 (METTL3-insensitive), CSC73 (METTL3-insensitive) and COLO201 (METTL3-sensitive) cells with two biological replicates for each in addition to HCT116 and HT29 (Figure 7B).

Figure 7

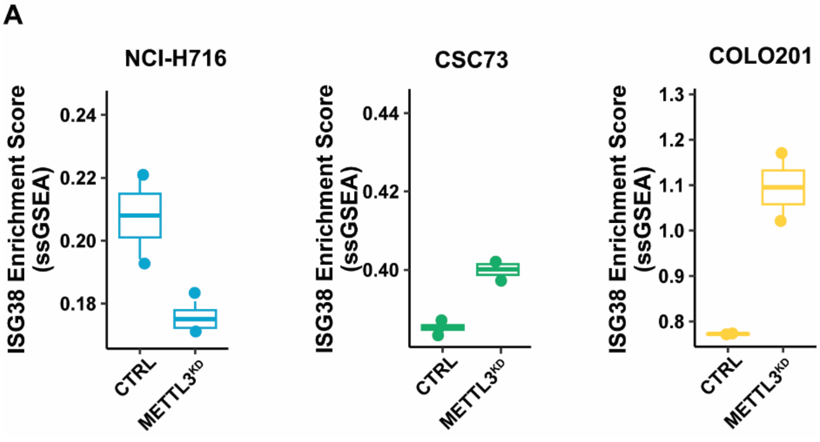

Figure 7. ISG38 expression changes across three CRC models. **(A)** ISG38 single-sample GSEA (ssGSEA) scores in NCI-H716, CSC73, and COLO201 colorectal cancer models. Data represent two independent experiments.

8)

277-279: "SINEs, particularly Alu elements, constituted the majority of MDA5-protected regions, and METTL3KD significantly increased the enrichment of these regions in HT29 CRC cells (Supplementary Figure 5B)."

Supplementary Figure 5B does not show any significant enrichment of these regions in METTL3 KD versus WT. Again, statistics are impossible with only a single line in each group, and the difference is minor and could be completely within the error of the assay or variance between lines. Once again, the conclusions presented in this section are uncertain in the absence of validation in other lines and rigorous statistical analysis.

- **To evaluate enrichment of repeat element classes, we applied a two-sided Fisher's exact test relative to the genomic representation of each repeat class, reporting both odds ratios (ORs) and P values. These analyses support our conclusion that SINEs, particularly Alu elements, represent the predominant class of MDA5-protected regions and are significantly enriched.**

  We agree that the proportional distribution of repeat classes is similar between CTRL and $METTL3^{KD}$ samples. However, METTL3KD resulted in a 1.7-fold increase in the total number of MDA5-protected repeat elements (73,134 vs. 42,418) (Supplementary Fig. 6B), paralleling the 1.7-fold increase in overall MDA5-protected regions (57,057 vs. 33,712) (Supplementary Fig. 6A). Thus, although class proportions remain stable, the overall abundance of MDA5-bound repeats increases markedly upon METTL3 depletion.

  This assay was performed in one CRC cell line with two biological replicates per condition. Given the technical complexity of the RNase/MDA5 protection assay, which requires large amounts of cytoplasmic RNA, recombinant MDA5 incubation, RNase A digestion, and sequencing, this replication level is consistent with prior applications of the method and yields robust results [9, 10].

  To avoid overinterpretation, we have revised the manuscript text to read: "SINEs, particularly Alu elements, constituted the majority of MDA5-protected regions, and $METTL3^{KD}$ resulted in a 1.7-fold increase in the total number of MDA5-protected repeat elements (73,134 vs. 42,418) (Supplementary Fig. 6B).

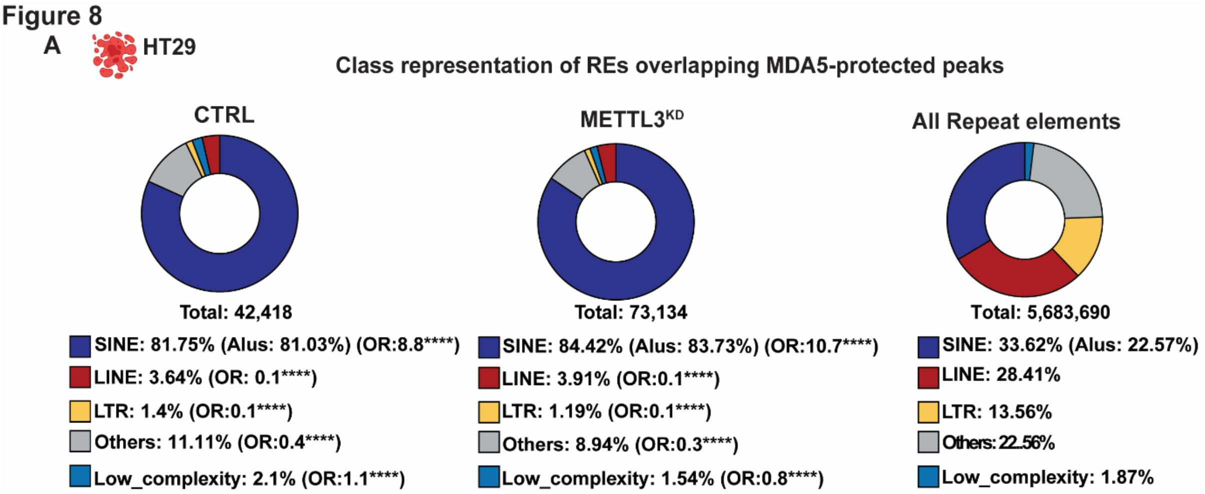

Figure 8. Class representation of REs overlapping MDA5-protected peaks. Donut plots showing the class representation of repeat elements which overlap with MDA5-protected regions for CTRL and METTL3^{KD} in HT29 cells. Odds ratio (OR) shows depletion or enrichment for each genomic category of Repeat elements in CTRL or METTL3^{KD} compared to the distribution of repeat elements present in the human genome. ****P < 0.0001, two-sided Fisher exact test.

9)

297-299: "This phenomenon appears to depend, at least in part, on METTL3 activity, whereby METTL3-mediated m6A modifications inhibit the formation of dsRNA from transcriptionally active REs. To test this hypothesis . . . "

Can references be provided here? This appears to be a well-established concept, albeit not necessarily the context of CRC.

- **We thank the reviewer for this thoughtful comment. While several studies have reported that METTL3 depletion can influence global dsRNA levels, to our knowledge none have directly demonstrated that METTL3-mediated m^{6}A modification prevents the formation of dsRNA specifically from transcriptionally active or newly transcribed repeat elements (REs). Prior work, including Gao et al. and Guirguis et al., showed dsRNA accumulation following METTL3 loss, but the dsRNA species described in those studies were derived predominantly from long protein-coding or non-coding transcripts rather than REs, and the mechanistic link between m^{6}A and RE-derived dsRNA folding was not examined.**

  Our study therefore builds on these observations by providing direct evidence that METTL3-dependent m^{6}A normally suppresses the dsRNA-forming potential of intronic IR-Alus in CRC cells. We have incorporated citations to the relevant prior studies in the Introduction and Discussion to provide appropriate context, while clarifying that the specific mechanism described in this section has not been previously established.

10)

313-317: "Collectively, our results demonstrate that METTL3 suppresses the viral mimicry responses by inhibiting the folding of IR-Alu transcripts into dsRNA structures (Figure 7D).

METTL3KD In METTL3-sensitive CRCs with higher basal RE expression and m6A modifications induces dsRNA formation, triggering viral mimicry and cancer cell death. In contrast, METTL3KD in METTL3-insensitive CRCs, characterized by lower RE expression and m6A modifications, fails to induce cancer cell death." Again, this needs to be validated in additional lines.

- We thank the reviewer for this important point. We assessed METTL3 loss across six CRC models using CellTiter-Glo and multiple orthogonal molecular assays (revised Figures 1D, and Supplementary Figures 1C). Overall, our results indicate that CRC models differ in their vulnerability to METTL3 targeting. METTL3-insensitive lines generally show lower J2-detectable dsRNA and reduced m^{6}A deposition (Revised figure 6D) and do not undergo significant cell death after METTL3 depletion, even when retroelement (RE) transcript levels are high (for example, NCI-H716).

  By contrast, METTL3-sensitive models tend to exhibit higher levels of RE-derived dsRNA and greater m^{6}A deposition and undergo robust cell death upon METTL3 loss. We therefore conclude that sensitivity is linked to m^{6}A-dependent dsRNA-forming competence rather than RE abundance per se. That said, we agree that these findings should be validated in larger panels and in primary tumour specimens, and we have added text to the Discussion to highlight this limitation.

11)

331-333: "Additionally, viability assays using CELL-TITER-Glo and colony-forming assays demonstrated that DAC treatment induced a synthetic lethality with METTL3 inhibition in both CRC cells (Supplementary Figures 7D-G)."

A statistical test for synergy (e.g., non-additive increase) should be performed to prove this. It appears this may be true for HCT116, but likely not HT29. What about the other sensitive and insensitive lines? Again, there must be validation in additional lines to prove that these effects are not idiosyncratic differences between HT29 and HCT116.

- We thank the reviewer for this comment. In HCT116 cells, the analysis demonstrated a non-additive (synergistic) decrease in viability, whereas in HT29 cells the combined treatment produced an additive but not statistically synergistic effect, consistent with the reviewer's observation. We agree with this point, and the terminology distinguishing additive from synergistic effects has been revised throughout the manuscript.

  As suggested, we extended the CTG analysis to additional CRC models, including METTL3-sensitive (COLO201 and POP92) and METTL3-insensitive (NCI-H716 and CSC73 PDX-derived) lines, under CTRL, METTL3^{KD}, METTL3^{KD} in combination with DAC, and DAC-only conditions. DAC increased cytotoxicity across all models; however, synergistic effects were observed specifically in METTL3-insensitive lines, while METTL3-sensitive models exhibited an additive response (Supplementary Fig. 8).

To clarify this point, we have revised the manuscript text as follows: "Consistent with these transcriptomic changes, CellTiter-Glo and colony-formation assays demonstrated that DAC treatment enhanced the anti-tumour effects of METTL3 inhibition in both METTL3-sensitive and METTL3-insensitive CRC models (Supplementary Fig. 8D–H), indicating that DNMT inhibition can overcome resistance to METTL3 targeting."

It should be noted that colony-forming unit assays could not be performed for suspension cells (e.g., NCI-H716, POP92, CSC73) due to their non-adherent growth properties. Therefore, CTG assays were used to quantitatively assess viability across all models, providing a robust and comparable readout.

12)

The presentation of Figures 1 and 3 feels disjointed, given that they make the same point but in a different set of cell lines. Figure 3 should not be a separate figure, but rather should be combined with Figure 1/Supplemental Figure 1.

- We thank the reviewer for this helpful suggestion. We agree that Figures 1 and 3 present complementary data addressing the same biological question across different CRC cell lines and PDX models. Accordingly, we have reorganized the figures to improve clarity and flow. The data previously shown in Figure 3 have now been incorporated into Figure 1 (and the relevant supplemental figure adjusted accordingly).

13)

Figure 4B-C could ideally be combined into one panel, with shape (e.g., circle versus triangle) denoting MSI status. No need for two separate panels.

- In accordance with the reviewer's suggestion, the two panels have been merged into a single panel (Figure 3B) in the revised manuscript, as shown below.

Figure 9

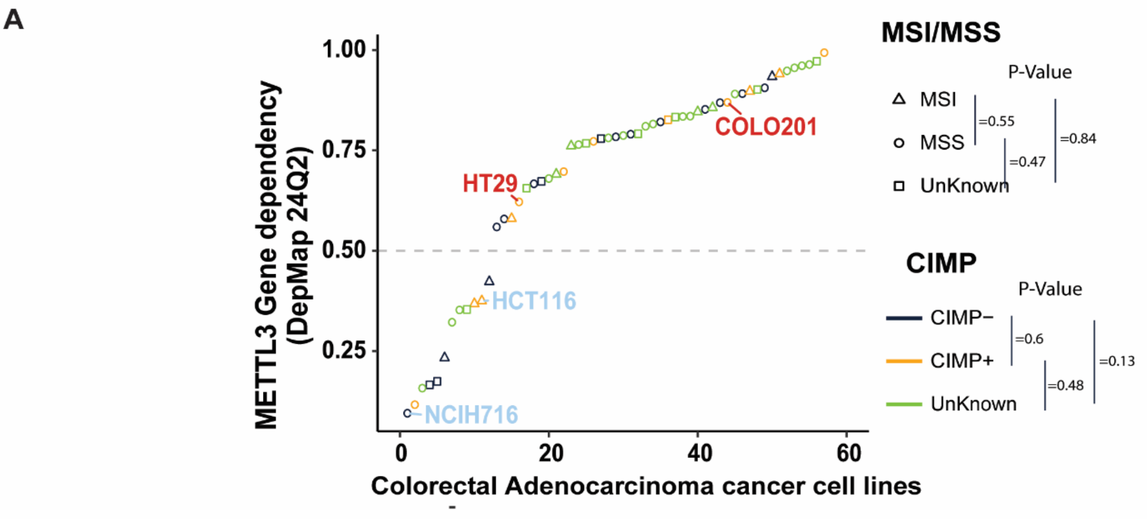

Figure 9. Scatter plot illustrating the dependency of colorectal adenocarcinoma (COAD) cell lines on METTL3 based on DepMap Public 24Q2 data.

(A) Cell lines are distinguished by shape according to microsatellite stability status (triangles: MSS; circles: MSI; squares: uncertain) and by color according to CpG island methylator phenotype (black: CIMP–; yellow: CIMP+; green: unknown). Statistical significance was assessed using the Wilcoxon rank-sum test, with the corresponding p-values displayed to the right of the legend.

14)

Figure 4D is more suited to supplementary figures.

- **We agree with the reviewer's comment and have moved Figure 4D to Supplementary Figure 3A in the revised manuscript.**

15)

Figure 4E is not particularly informative as presented. Choosing the top 20 genes based on mutation frequency tends to enrich for many non-relevant/non-driver genes that are simply very large and thus neutrally accumulate many mutations (e.g., TTN, OBSCN). Inclusion of these genes may obscure potentially meaningful differences. If the authors really want to make the point that there is no difference in mutational profile, a more earnest analysis should be performed. For example, prioritization of significantly recurrently mutated driver genes should be performed by using MutSigCV (Lawrence, MS, et al., Nature, 2013) (such analysis has been previously performed in CRC and the driver gene list(s) can be pulled from these studies) and only these included in the analysis. Then, a gene-by-gene analysis of mutation rates in DepMap METLL3 KO sensitive versus insensitive cell lines should be performed to identify any mutant gene-specific enrichments.

Relatedly, only 4 of the cell lines, albeit the ones included in the experiments in this study (COLO201, HT29, HCT116, NCIH716) are highlighted in the plot. Can the authors plot the cell line key bar to show sensitive versus insensitive lines? While it is noted in the legend that the lines are ordered by sensitivity score, where do the insensitive end and the sensitive begin (based on the dotted line in Figure 4B-C)? If there are truly no patterns to report here, this figure should be put in the supplementary figures.

- **We appreciate the reviewer's suggestions. In the revised manuscript, we incorporated the 131 significantly mutated genes in colorectal adenocarcinoma identified by the Broad Institute TCGA MutSigCV analysis [1]. From this list, we selected genes mutated in at least 15% of colorectal cancer cell lines, yielding a focused set of 35 recurrently altered genes. We then compared the mutational profiles of METTL3-sensitive versus insensitive CRC models.**
  Overall, METTL3-sensitive cell lines showed a higher frequency of mutations across these genes than insensitive lines. For example, TP53 mutations were observed in 84% of sensitive models but only 58% of insensitive ones. Importantly, no single driver mutation reliably predicted sensitivity to METTL3 inhibition. The pattern was highly variable: HT29, a METTL3-sensitive line, carried only 4 of the 35 recurrent mutations, whereas HCT116, an insensitive line, carried 13. These findings support our conclusion that CRC sensitivity to METTL3 targeting is not determined by

individual driver mutations but more likely reflects broader epigenetic or transcriptomic features.

[1] Broad Institute TCGA Genome Data Analysis Center (2016): Mutation Analysis (MutSigCV v0.9). Broad Institute of MIT and Harvard. doi:10.7908/C1G44PPJ

Figure 10

A

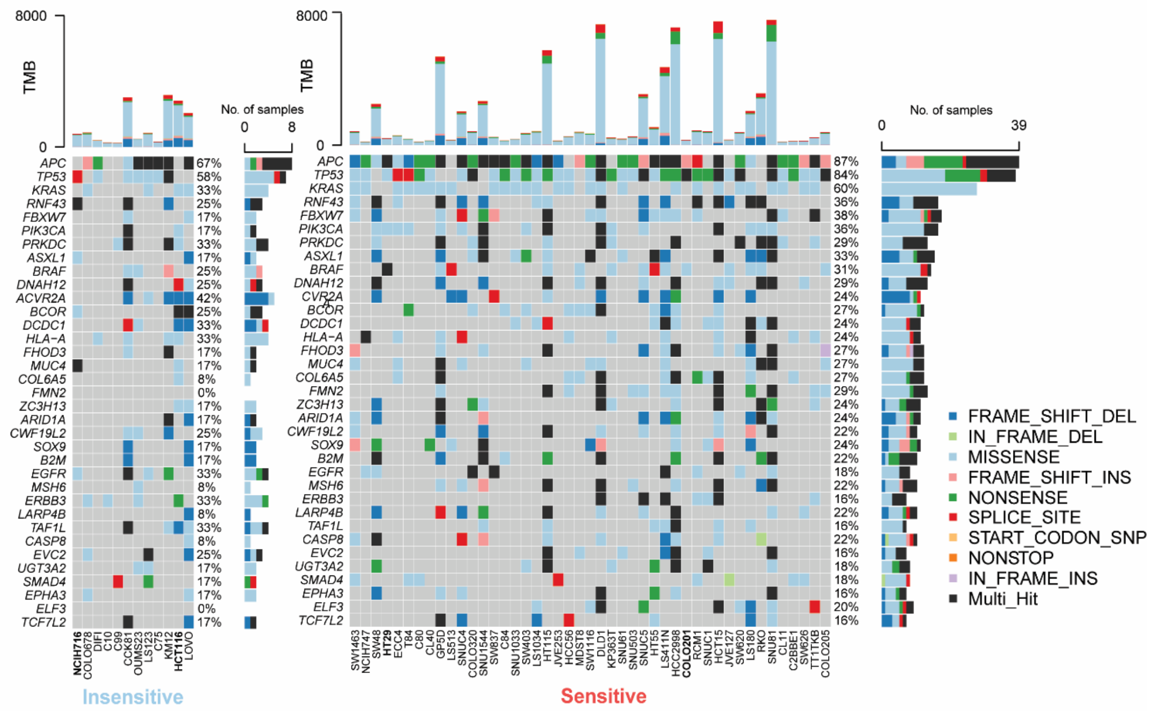

Figure 10. Oncoplots comparing mutation profiles of 35 genes between METTL3-targeting sensitive (dependency score > 0.5) and insensitive (dependency score < 0.5) colorectal cancer cell lines. The 35 genes were selected based on their mutation frequency (≥15% of colorectal cancer cell lines) and inclusion in the list of 131 significantly mutated genes in colorectal adenocarcinoma identified by the Broad Institute TCGA MutSigCV analysis **[11]**. While certain mutations such as *TP53* appeared more frequent in METTL3-sensitive cell lines, no single recurrent mutation pattern was found to explain METTL3 dependency.

16)

391-393: "In cancer cells, however, targeting METTL3 and the loss of m6A induces conformational changes in these IR-Alus, enabling the formation of dsRNA stem-loops and ultimately leading to dsRNA accumulation." Were conformational changes experimentally demonstrated, or are they being assumed?

- **We thank the reviewer for this important point. We agree that the original phrasing could imply direct visualization of RNA structural changes, which we did not perform. Our conclusions are instead based on multiple orthogonal assays that consistently indicate increased formation or persistence of dsRNA structures following METTL3 depletion.**

  Specifically, METTL3^{KD} led to a robust increase in J2-positive dsRNA by immunofluorescence and dot blot (Figure 1G and Supplementary Fig 2B). These

effects occurred without detectable changes in RNA half-life, as shown by Actinomycin D chase experiments for indicated repeat-derived RNAs (Supplementary Fig. 7A), arguing against a stability-based explanation. Furthermore, $METTL3^{KD}$ reduced m^6A specifically within the J2-bound fraction (Figure 6A, B), consistent with the accumulation of hypomethylated dsRNAs that are more efficiently recognized by J2 and MDA5.

Although RNAfold did not predict global minimum-free-energy differences between methylated and unmethylated sequences, which is expected because m^6A primarily affects local and kinetic RNA folding rather than global thermodynamics, prior biochemical studies have shown that m^6A weakens base stacking and destabilizes local duplex structures [2, 3]. These data support the plausibility that loss of m^6A favors the formation or persistence of dsRNA conformations. The stem-loop structures we refer to are inferred from the RNase/MDA5 protection assay, which selectively enriches dsRNAs derived from convergently oriented IR-Alu elements, consistent with intramolecular pairing rather than increased transcription.

- We have revised the manuscript text to state: "Mechanistically, our findings suggest how m^6A influences the structural fate of IR-Alu–containing transcripts. Biochemical and structural studies have shown that m^6A destabilizes RNA duplexes by disrupting base stacking and slowing annealing kinetics [2-4, 12], providing a biophysical rationale for how loss of m^6A promotes the formation or persistence of IR-Alu–derived dsRNAs. Although global RNA structure-mapping approaches such as DMS-seq or SHAPE-seq could further refine this model, these methods cannot readily distinguish immunostimulatory IR-Alu dsRNAs from other structured RNAs. In this context, our targeted RNase/MDA5 protection assay is particularly informative, as it selectively enriches dsRNAs capable of engaging innate immune sensors."

Figure 11

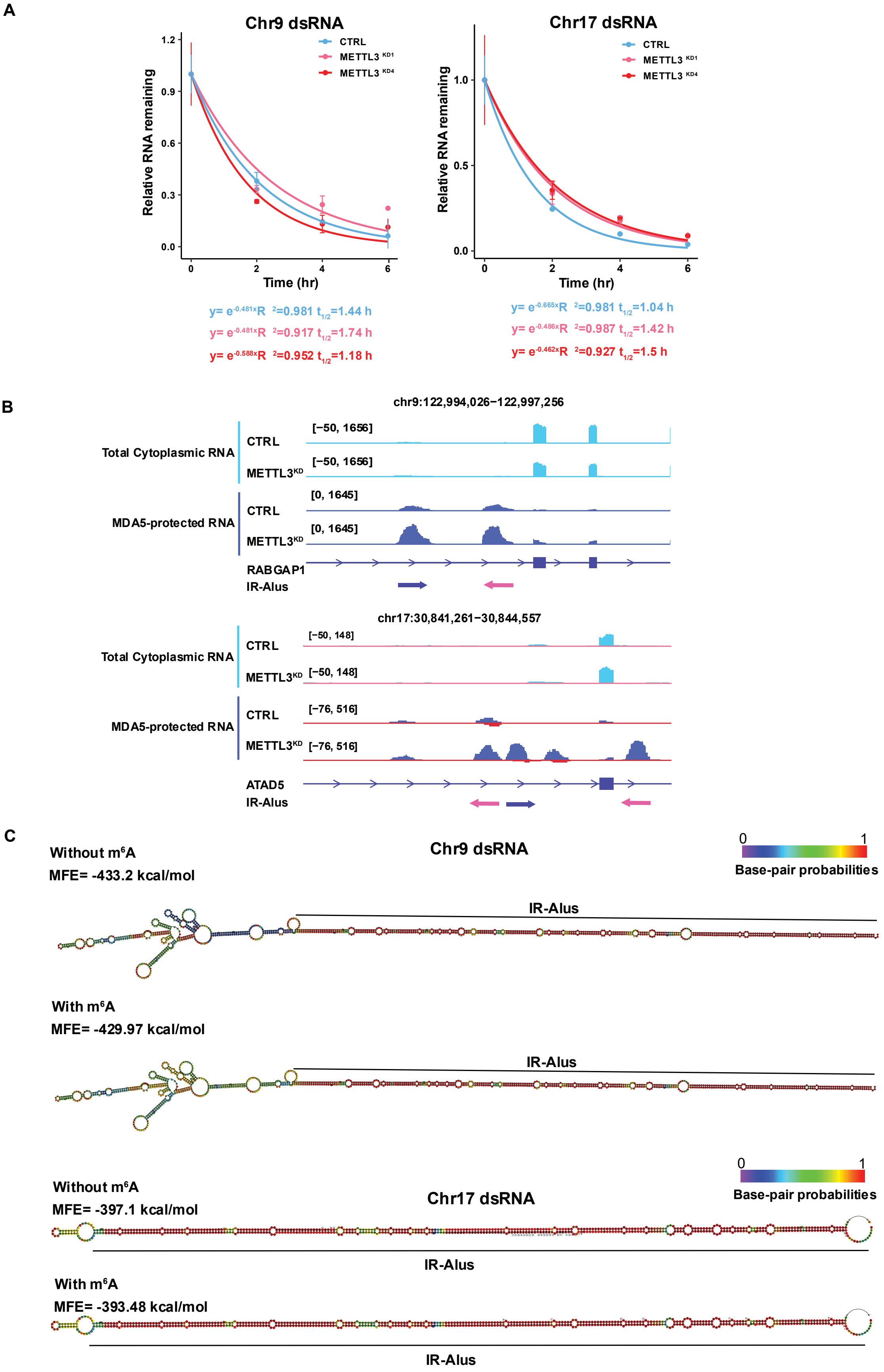
A
Chr9 dsRNA
CTRL
METTL3 KD1
METTL3 KD4
Relative RNA remaining
Time (hr)
y= e-0.481xR 2=0.981 t1/2=1.44 h
y= e-0.481xR 2=0.917 t1/2=1.74 h
y= e-0.588xR 2=0.952 t1/2=1.18 h
Chr17 dsRNA
y= e-0.665xR 2=0.981 t1/2=1.04 h
y= e-0.486xR 2=0.987 t1/2=1.42 h
y= e-0.462xR 2=0.927 t1/2=1.5 h
B
chr9:122,994,026−122,997,256
Total Cytoplasmic RNA
MDA5-protected RNA
CTRL
METTL3KD
[−50, 1656]
[0, 1645]
RABGAP1
IR-Alus
chr17:30,841,261−30,844,557
[−50, 148]
[−76, 516]
ATAD5
C
Without m6A
MFE= -433.2 kcal/mol
With m6A
MFE= -429.97 kcal/mol
Chr9 dsRNA
Base-pair probabilities
Without m6A
MFE= -397.1 kcal/mol
With m6A
MFE= -393.48 kcal/mol
Chr17 dsRNA

Figure 11. METTL3 depletion increases hypomethylated IR-Alu–derived dsRNAs without altering RNA stability in HT29.

(A) HT29 control and METTL3^{KD} cells were treated with actinomycin D (1 µg/mL), and RNA was extracted at the indicated time points for RT–qPCR analysis. The degradation rate of each transcript was fitted using $y = e^{-k(decay)x}$. Fits with $R^2 \geq 0.95$ were considered robust; RNA half-lives were calculated as $t_1/_2 = ln(2) / k(decay)$. $n = 2$ technical replicates.

(B) Representative genomic tracks showing METTL3^{KD}-induced immunostimulatory inverted-repeat Alu (IR-Alu) elements highlighted in pink and blue. The top two tracks display total cytoplasmic RNA with sense (sky blue) and antisense (pink) reads distinguished. The next two tracks show RNase A–digested cytoplasmic RNA, with sense (dark blue) and antisense (red) reads indicated.

(C) Representative examples of predicted dsRNA structures formed by IR-Alu pairs, shown either in their unmethylated form or with all adenosines in m^{6}A motif (DRACH) substituted to mimic m^{6}A-modified positions. RNAfold-predicted secondary structures and corresponding minimum free energy (MFE)are shown for an IR-Alu locus on chromosome 9 and chromosome 17.

Reviewer #3 (Remarks to the Author):

This manuscript presents a well-executed study investigating how METTL3-mediated m6A RNA modification suppresses viral mimicry responses in colorectal cancer by preventing dsRNA formation from Alu repeats.

Major Comments

1. While the authors demonstrate that m6A loss enables IR-Alu folding into dsRNA, additional structural or biophysical studies could provide deeper insights into how m6A modifications specifically prevent these secondary structure formations, such as DMS-seq or SHAPE-related method to determine the RNA structural maps.

2. Similarly, dsRNA was detected via RNase/MDA5 protection assays. Could transcriptome-wide RNA-structure profiling (e.g. chemical probing and sequencing) be employed? Given the intronic enrichment of dsRNA signals post-treatment (Fig 6D), precise mapping and in-depth characterization of their genomic origins would strengthen the study.

- **We thank the reviewer for these excellent suggestions. We fully agree that genome-wide RNA-structure profiling approaches such as DMS-seq, SHAPE-seq, or related chemical-probing methods would provide deeper structural insight into how m^6A prevents IR-Alu elements from adopting double-stranded conformations. These approaches would indeed further elucidate the precise structural transitions underlying dsRNA formation. However, implementing these experiments, requiring optimization of chemical probing conditions, library preparation, deep sequencing, and extensive computational modelling, is not feasible within the timeframe of the current revision and is therefore beyond the scope of this study.**

  Importantly, the mechanistic premise that m^6A impedes RNA duplex formation is strongly supported by existing biochemical and structural literature. Prior NMR and biophysical analyses have demonstrated that m^6A disrupts local base stacking, weakens duplex stability, and slows RNA annealing kinetics [2, 3, 13]. In parallel, functional studies have shown that loss of METTL3 or m^6A increases endogenous dsRNA abundance and activates MDA5-dependent antiviral signaling [14]. These published data form a robust mechanistic foundation for our model.

  In our study, we provide direct, orthogonal experimental evidence that METTL3 loss enhances dsRNA accumulation from transcriptionally active repeat elements. This includes J2 immunofluorescence, J2-IP, RNase/MDA5 protection assays, and locus-resolved mapping of enriched dsRNAs, including the observed intronic enrichment (Figure 6D). Although transcriptome-wide structural profiling would offer nucleotide-level resolution of these dsRNA regions, our combined RNA-seq, RNase protection, and MDA5-dependence analyses already define the genomic origins and biochemical properties of the dsRNA species induced by METTL3 depletion.

  Finally, while transcriptome-wide structural profiling (e.g., DMS-seq or SHAPE-seq) would provide additional nucleotide-level information, these approaches do not easily discriminate immunostimulatory dsRNAs produced by IR-Alus from the many structured RNAs present throughout the transcriptome. In contrast, the MDA5/RNase protection assay used in our study selectively enriches long dsRNA species that are bound and protected by MDA5, thereby isolating the functionally

relevant dsRNA pool that drives innate immune activation. This allows us to specifically identify the dsRNAs that accumulate upon METTL3 depletion and are responsible for triggering the MAVS-dependent response.

To address the reviewer's comment directly, we have added citations to the relevant studies and incorporated a clarifying statement in the revised manuscript text.

3. METTL3 knockdown may affect other RNA metabolism pathways beyond dsRNA formation. The authors should clarify whether the observed cell death is primarily due to viral mimicry or involves additional stress response mechanisms.

- We thank the reviewer for this insightful comment. We agree that METTL3 knockdown could, in principle, influence additional RNA-metabolism pathways beyond dsRNA formation. To directly determine whether the cytotoxicity we observe is primarily driven by viral mimicry, we performed genetic epistasis experiments in PKR knockout (PKR^{KO}), RNase L knockout ($RNaseL^{KO}$), and MAVS knockout ($MAVS^{KO}$) cells, individually and in combination with METTL3 knockout ($METTL3^{KO}$) (Figure 2E–H).
  Importantly, cell death was markedly reduced in PKR/METTL3 double-knockout (^{DKO}) cells, demonstrating that activation of the PKR-dependent antiviral pathway is a major contributor to METTL3-loss–induced cytotoxicity. Likewise, MAVS loss strongly attenuated ISG induction, confirming that canonical dsRNA sensing is required for the antiviral transcriptional response. RNase L depletion produced a more modest rescue, consistent with PKR rather than RNase L being the dominant effector.

  Together, these data indicate that viral mimicry is the primary driver of the cell-death phenotype following METTL3 loss. Nonetheless, because METTL3 regulates several facets of RNA metabolism, we recognize that we cannot fully exclude the possibility that additional stress-response pathways contribute secondarily to this outcome.

4. METTL3 carries widespread beneficial functions, so its direct inhibition may elicit unintended adverse effects. In the authors' previous work (ref 9, Nature), targeting ADAR1 was shown to represent a promising strategy for enhancing antitumor immunity; unlike ADAR1, METTL3 lacks intrinsic dsRNA-specific binding capacity. Cell-level assays alone cannot reveal safety impacts at the tissue, organ, or organismal level.

- We thank the reviewer for this insightful comment. We agree that METTL3 plays broad physiological roles and that its systemic inhibition may have wider consequences than targeting ADAR1, which directly edits and suppresses immunogenic dsRNAs. However, the comparative therapeutic windows of METTL3 and ADAR1 inhibition remain unresolved, as neither enzyme has been comprehensively evaluated in normal tissues in vivo.

  Importantly, small-molecule METTL3 inhibitors have already entered phase I clinical trials for hematologic malignancies and solid tumors (e.g., NCT05584111, NCT05640311), indicating that METTL3 is currently being clinically explored and that

its tolerability is under active investigation. In contrast, no ADAR1 inhibitors have yet reached clinical testing, and potential toxicities associated with globally unleashing endogenous dsRNA in healthy tissues remain unknown.

Our study was therefore motivated not by the assumption that METTL3 is inherently safer than ADAR1, but by the need to understand whether CRC tumors exhibit a therapeutically actionable dependency on METTL3 and to define the mechanisms by which METTL3 regulates repeat-derived dsRNA in this context. In addition, METTL3 and ADAR1 appear to functionally intersect in regulating endogenous dsRNAs, yet this relationship has not been explored in colorectal cancer. Understanding this interplay is essential for evaluating whether METTL3 inhibition alone or in combination with other immunomodulatory strategies may have therapeutic benefit.

Together, these considerations justify our focus on METTL3 in the present study: (i) METTL3 is an actively pursued clinical target, (ii) the impact of METTL3 inhibition in CRC was unknown, and (iii) defining how METTL3 regulates repeat-element–derived dsRNA provides mechanistic insight relevant for evaluating both efficacy and potential safety consideration.

5. The basis for selecting a DNMT inhibitor (DNMTi) and its mechanism of enhancement are not experimentally validated. The choice appears based only on literature precedent rather than systematic screening, and a DNMTi-only control is missing.

- We thank the reviewer for this thoughtful comment. Our use of a DNMT inhibitor (DNMTi) was motivated by extensive prior evidence demonstrating that Decitabine (DAC) induces viral mimicry by activating repeat element–derived dsRNAs in colorectal cancer and multiple other tumor types. This mechanism has been well established by our group and by several independent studies [10, 15-17]. Tor this reason, we used DAC as a validated, mechanistically understood positive control to induce endogenous dsRNA, rather than performing additional compound screening.

  Our goal in this study was to test whether increasing basal levels of immunostimulatory dsRNA could sensitize METTL3-insensitive CRC cells to METTL3 loss. DAC provides a robust and reproducible way to achieve this, allowing us to directly evaluate the combinatorial effect. As noted in the Discussion, other agents that elevate dsRNA abundance could also be explored in future work to identify the most effective therapeutic combination partne

  A DNMTi-only control was included in all relevant experiments, referred to as CTRL-DAC–treated, and is presented in Figures 7, 8 and Supplementary Figure 8. <u>We have clarified this point in the Methods and Figure legends to make this explicit.</u>

Minor Comments：

1. **Pg. 34, line 22, "The top 20 most mutated genes " showed "21 genes ".**
    - We thank the reviewer for spotting this error. The figures have been regenerated and the numbers have been corrected accordingly.

2. **MeRIP-seq Technical Issue (Lines 708-711),The observation that "the first read from paired-end sequencing exhibited lower sequencing quality" in MeRIP samples is indeed unusual and warrants further explanation.**
    - We thank the reviewer for this comment and apologize for the confusion. The lower sequencing quality observed in the first read was not unexpected and does not impact the analysis, as all analyses were performed using single-end data (the second read), although both reads were deposited in GEO. The libraries were generated using the NEBNext® Ultra™ II Directional RNA Library Prep Kit for Illumina® (Cat. #E7760L) and the NEBNext® Multiplex Oligos for Illumina® (Index Primer Set 1; Cat. #E7335S/L). Because the NEBNext Ultra II Directional protocol produces strand-specific libraries and MeRIP-seq fragments are short and enriched for sequence-biased regions, differences in base quality between Read 1 and Read 2 are common. In our dataset, Read 2 displayed higher overall sequencing quality, and therefore single-end analyses were performed using Read 2 only. The study was designed to use single-end sequencing; however, the sequencing run was carried out in paired-end mode. We have now corrected and clarified this point in the Methods section.

3. **The Introduction does not justify the focus on colorectal cancer—e.g. its prevalence, urgency of progression, or treatment challenges. Figure S1A shows that METTL3 is overexpressed across most cancer types. To support broad applicability, additional cancer cell lines should be evaluated.**
    - We thank the reviewer for this valuable comment and agree that additional context strengthens our rationale for focusing on colorectal cancer (CRC). CRC is the third most commonly diagnosed cancer worldwide and the second leading cause of cancer-related mortality [18]. Despite therapeutic advances, relapse rates remain high, and epigenetic therapies that are effective in hematologic malignancies have shown limited benefit in solid tumors, including CRC Furthermore, the rising incidence of CRC in younger adults highlights an urgent need to identify new therapeutic vulnerabilities [19].

      Our study therefore centers on CRC to determine whether METTL3 inhibition could represent a viable therapeutic strategy in this clinically significant and therapeutically challenging disease. To capture biological diversity and enhance translational relevance, we included four independent CRC cell lines and two patient-derived xenograft (PDX) models that span distinct molecular and phenotypic backgrounds.

      We appreciate the suggestion to include an additional cancer type; however, doing so would require establishing and validating an entirely separate

experimental system, which is beyond the scope of this manuscript. Our goal here is to provide a focused mechanistic and functional analysis within CRC, where the clinical need and disease-specific context justify this dedicated investigation.
We have now revised the Introduction to include these points and better justify our focus on CRC.

4. **Line 62: Please add an appropriate reference.**
   - We apologise for this omission and have added the appropriate citation in the revised manuscript.

5. **Line 197: This statement lacks supporting data or statistical evidence; please provide a figure or analysis.**
   - We apologise for the confusion. We observed higher METTL3 protein levels in CRC cells compared to normal colon cells (Figure 3A). However, quantification of METTL3 protein intensity relative to the loading control showed no significant association between METTL3 abundance and CRC cell sensitivity to METTL3 targeting (Figure 3A). The corresponding densitometric analysis has now been added to the revised figure and is shown below.

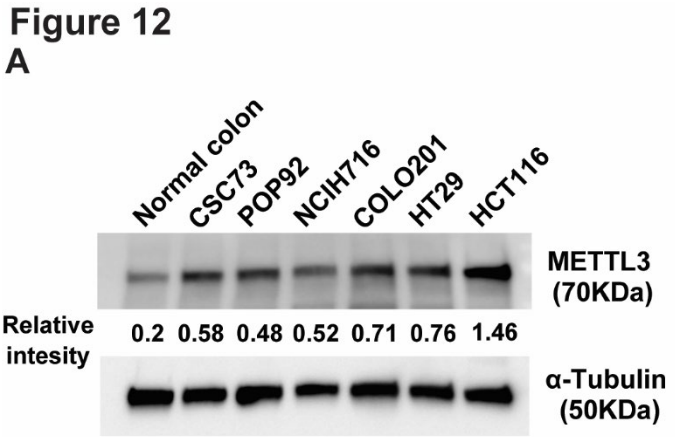

Figure 12. Immunoblot analysis of METTL3 protein expression in normal colon cells, CSC73 and POP92 PDX colorectal cancer cells, and CRC cell lines (NCI-H716, COLO201, HT29, and HCT116). Anti–α-tubulin was used as a loading control. The relative intensity of METTL3 normalized to α-tubulin for each lane was quantified using ImageLab software.

6. **Figs 1A, 4B & 4C: Provide supplementary tables with the underlying data so readers can examine additional cell lines of interest.**
   - We thank the reviewer for this helpful suggestion. In the revised manuscript, we have added Supplementary Table 6, which lists all colorectal cancer cell lines from DepMap along with their METTL3 dependency probability, MSS/MSI status, CIMP status, and CMS subtype classification, as shown below.

Order	Cancer cell line	Dependency Probability	MSS/MSI	CIMP	CMS	KRAS mutation status
1	NCIH716	0.0951	MSS	CIMP-	CMS1	Wild type
2	COLO678	0.1165	MSS	CIMP+	CMS2	Mutated
3	DIFI	0.1576	MSS	Unknown	Unknown	Wild type
4	C10	0.1659	Unknown	CIMP-	CMS1	Wild type
5	C99	0.1744	Unknown	CIMP-	Unknown	Wild type
6	CCK81	0.2331	MSI	CIMP-	CMS2	Wild type
7	OUMS23	0.3219	MSS	Unknown	CMS2	Wild type
8	LS123	0.3525	MSS	Unknown	CMS1	Mutated
9	C75	0.3536	Unknown	Unknown	CMS2	Wild type
10	KM12	0.3681	MSI	CIMP+	CMS1	Wild type
11	HCT116	0.3751	MSI	CIMP+	CMS1	Mutated
12	LOVO	0.4229	MSI	CIMP-	CMS1	Mutated
13	SW1463	0.5593	MSS	CIMP-	Unknown	Mutated
14	NCIH747	0.5792	MSS	CIMP-	CMS1	Mutated
15	SW48	0.5802	MSI	CIMP+	CMS1	Wild type
16	HT29	0.6218	MSS	CIMP+	Non-consensus	Wild type
17	ECC4	0.6558	Unknown	Unknown	Unknown	Mutated
18	T84	0.6668	MSS	CIMP-	CMS2	Mutated
19	C80	0.6728	Unknown	CIMP-	Unknown	Mutated
20	CL40	0.6799	MSS	Unknown	CMS3	Mutated
21	GP5D	0.6908	MSI	Unknown	CMS2	Mutated
22	LS513	0.6972	MSS	CIMP+	CMS3	Mutated
23	SNUC4	0.7612	MSI	Unknown	Non-consensus	Wild type
24	COLO320	0.7645	MSS	Unknown	CMS2	Wild type
25	SNU1544	0.7677	Unknown	Unknown	CMS1	Wild type
26	SW837	0.7725	MSS	CIMP+	Unknown	Mutated
27	C84	0.7795	Unknown	CIMP-	CMS2	Mutated
28	SNU1033	0.7812	MSS	Unknown	Unknown	Mutated
29	SW403	0.7838	MSS	CIMP-	CMS2	Mutated
30	LS1034	0.7872	MSS	Unknown	CMS2	Mutated
31	HT115	0.7904	MSS	CIMP-	Non-consensus	Wild type
32	JVE253	0.7915	Unknown	Unknown	Unknown	Mutated
33	HCC56	0.8102	MSS	Unknown	CMS2	Mutated
34	MDST8	0.8158	MSS	Unknown	CMS1	Wild type
35	SW1116	0.8209	MSS	CIMP-	CMS2	Mutated
36	DLD1	0.8264	Unknown	CIMP+	CMS1	Mutated
37	KP363T	0.8331	Unknown	Unknown	CMS1	Wild type
38	SNU61	0.8345	MSS	Unknown	Unknown	Mutated
39	SNU503	0.8354	MSS	Unknown	Unknown	Wild type
40	SNUC5	0.8462	MSI	Unknown	CMS1	Wild type
41	HT55	0.8523	MSS	CIMP-	CMS2	Wild type
42	LS411N	0.8567	MSI	Unknown	CMS1	Wild type
43	HCC2998	0.869	MSS	CIMP-	Unknown	Mutated

44	COLO201	0.8696	MSS	CIMP+	Non-consensus	Wild type
45	RCM1	0.8906	MSS	Unknown	Unknown	Mutated
46	SNUC1	0.8916	MSS	CIMP-	CMS2	Wild type
47	HCT15	0.8973	MSI	CIMP+	CMS1	Mutated
48	JVE127	0.9015	Unknown	Unknown	Unknown	Wild type
49	SW620	0.906	MSS	CIMP-	CMS2	Mutated
50	LS180	0.934	MSI	CIMP-	CMS3	Mutated
51	RKO	0.9408	MSI	CIMP+	CMS1	Wild type
52	SNU81	0.9483	MSS	Unknown	CMS1	Mutated
53	CL11	0.9558	MSS	Unknown	Non-consensus	Mutated
54	C2BBE1	0.961	MSS	Unknown	CMS2	Wild type
55	SW626	0.9641	MSS	Unknown	CMS2	Mutated
56	TT1TKB	0.9721	Unknown	Unknown	Unknown	Mutated
57	COLO205	0.9936	MSS	CIMP+	Unknown	Wild type

Reviewer #4 (Remarks to the Author):

In this manuscript, the authors elucidate a mechanism by which inhibition of METTL3 increases dsRNA levels, thereby activate the immune response. They demonstrate that METTL3 suppresses dsRNA formation particularly that derived from IR-Alus, via m6A methylation in colorectal cancer. They investigate that CRC cells exhibiting higher basal levels of retroelements and m6A modification display greater dependency on METTL3.

Importantly, they find that activating REs using DNMTi can sensitize previously insensitive CRC cells to METTL3 targeting, suggesting a promising treatment of combination therapy strategy. Overall, this is a well-written and presented study that propose a promising therapeutic approach CRC. However, a significant concern is the similarity to the findings reported by Yimeng Gao et al. 1, who also suggested that m6A modification mediated by METTL3 prevents formation of endogenous double-stranded RNA (dsRNA). The authors should thoroughly address this by comparing the two studies in discussion section, specifically highlighting the contribution of IR-Alu among dsRNAs in CRC.

- We thank the reviewer for this thoughtful comment.
  Gao et al. reported that loss of METTL3 during murine fetal hematopoiesis leads to accumulation of endogenous dsRNA and activation of innate immune signaling, with J2-immunoprecipitated dsRNAs derived largely from protein-coding transcripts [14]. While this study highlights a conserved role for METTL3 in restricting dsRNA formation, our work extends these observations to human colorectal cancer and identifies a mechanistically distinct source of immunogenic dsRNA. Specifically, we demonstrate that METTL3 depletion in CRC promotes the folding of inverted-repeat Alu (IR-Alu)–containing transcripts into dsRNA structures, independent of changes in transcript abundance. This represents a key difference from Gao et al., likely reflecting both the divergent biological contexts (murine fetal liver versus human CRC) and the unique abundance and evolutionary expansion of Alu elements in primate genomes.
  Methodologically, our study also provides complementary insight. By integrating m^6A profiling with RNase/MDA5 protection assays, we identify the subset of dsRNAs that directly engage MDA5, rather than the full dsRNA pool captured by J2-RIP. This approach allowed us to pinpoint IR-Alus as the predominant immunostimulatory species driving viral mimicry upon METTL3 loss in CRC cells. Beyond defining dsRNA origins, our study demonstrates that CRC cells exhibit heterogeneous dependency on METTL3, with sensitivity linked to basal retroelement activity. Finally, we show that DNMT inhibitors, established inducers of retroelement-derived dsRNA, synergize with METTL3 targeting to overcome resistance in METTL3-insensitive CRC models. Together, our findings expand the mechanistic understanding of METTL3-mediated dsRNA suppression and highlight IR-Alus as a critical and cancer-specific driver of viral mimicry in colorectal cancer.

In the revised manuscript, we have added a dedicated paragraph to the Discussion that directly compares our findings with those of Gao et al.:

- "Gao et al. previously demonstrated that METTL3 loss during murine fetal hematopoiesis leads to the accumulation of dsRNAs derived predominantly from protein-coding transcripts [27]. While these findings highlight a conserved role for METTL3 in limiting dsRNA formation, our results identify a mechanistically distinct source of immunogenic dsRNA in human CRC. Through integration of m^6A profiling with RNase/MDA5 protection assays, we show that IR-Alu elements constitute the primary MDA5-engaging dsRNAs activated upon METTL3 loss, an outcome likely influenced by both the differing biological contexts and the primate-specific expansion of Alu repeats. We further demonstrate substantial heterogeneity across CRC models in their capacity to generate dsRNA and show that DNMT inhibition, a known activator of retroelement dsRNA, synergizes with METTL3 targeting to overcome resistance".

I have a few additional comments that should be addressed.

1. **Line 132, is the minimal effect of METTL3 ko in HCT116 cells reliable? Chaoqin Shen et.al showed significant transcriptomic changes ("a total of 2848 downregulated genes and 3046 upregulated genes ($p < 0.05$) were detected (raw data accessible via GEO number GSE130012) after knockout of METTL3 in HCT116 cells"). Do other insensitive cell lines such as NCI-H716 and 183 CSC73 cells also exhibit similar minimal effects following METTL3 KO?**
   - We thank the reviewer for this thoughtful question. The discrepancy with Shen et al. [6] is largely due to different statistical thresholds. Their reported numbers were based solely on p-values, without considering fold changes. We reanalyzed their sequencing data using the same computational pipeline as in our manuscript. By applying criteria of FDR < 0.05 and absolute fold change ≥ 1, we identified 653 upregulated and 461 downregulated genes, which is consistent with the modest transcriptomic effect we observe with $METTL3^{KD}$ in HCT116. Although $METTL3^{KO}$ in our hands produces a larger number of deregulated genes, the phenotype remains identical to KD.

     To further address this point, we generated RNA-seq profiles for two additional METTL3-insensitive CRC lines (NCI-H716 and CSC73), now included in the revised manuscript (Supplementary Figure 1E). Both show moderate but non–pathway-defining changes and no enrichment of antiviral signaling upon METTL3 loss. In contrast, HCT116 displays a small but detectable sublethal interferon signature.

     Together, these data show that METTL3-insensitive CRC models exhibit heterogeneous but limited transcriptomic responses, and that their viability phenotype reflects a failure to activate cytotoxic viral mimicry rather than a lack of gene expression changes.

Figure 13

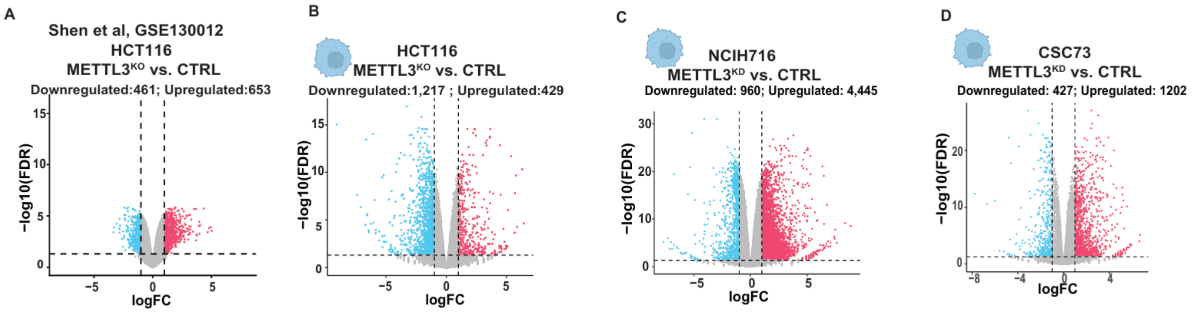

Figure 13. METTL3-Targeting Effects on Deregulated Gene Numbers Compared With Shen et al., and RNA-seq of METTL3-Insensitive CRCs.

(A) Volcano plot showing changes in gene expression by comparing METTL3^{KO} to CTRL in HCT116 cells, RNAseq data from GSE130012 which was generated by Shen et al. Significance was defined as FDR < 0.05 and |logFC| ≥ 1. n=3.

(B) Volcano plot showing changes in gene expression by comparing METTL3^{KO} to CTRL in HCT116 cells. Significance was defined as FDR < 0.05 and |logFC| ≥ 1. n=2.

(C) Volcano plot showing changes in gene expression by comparing METTL3^{KD} to CTRL in NCIH716 cells. Significance was defined as FDR < 0.05 and |logFC| ≥ 1. n=2.

(D) Volcano plot showing changes in gene expression by comparing METTL3^{KD} to CTRL in CSC73 cells. Significance was defined as FDR < 0.05 and |logFC| ≥ 1. n=2.

2. **For IFNβ promoter luciferase reporter assay, authors used total RNA isolated from cells. Using MDA5-captured dsRNA would provide more direct evidence.**

- **We thank the reviewer for this valuable suggestion. We fully agree that isolating MDA5-bound dsRNA would, in principle, provide more direct evidence of immunostimulatory RNA species. However, in our hands, MDA5 immunoprecipitation (IP) under native conditions, using either tagged or endogenous MDA5, has consistently failed to recover RNA that retains activity in IFNβ promoter luciferase reporter assays. This likely reflects the biochemical nature of MDA5–RNA interactions: MDA5 forms stable filamentous assemblies along long dsRNA molecules and functions as a sentinel that surveys cellular RNA homeostasis. These complexes are inherently fragile and often dissociate during IP, resulting in loss of bound RNA.**

  While crosslinking (e.g., with formaldehyde or UV) could, in theory, stabilize MDA5–RNA complexes, such treatments are known to compromise RNA integrity and immunostimulatory activity, precluding functional downstream assays. In contrast, RIG-I IP typically preserves shorter dsRNA ligands compatible with functional recovery, which explains why the approach works better for RIG-I than for MDA5.

  Given these technical constraints, we believe that the use of total cellular RNA provides a reliable and physiologically relevant readout of endogenous immunostimulatory RNA activity. We have clarified this point in the Methods and Discussion.

3. **The authors propose that higher basal expression of REs and m6A is major reason of sensitivity of CRCs to METTL3 targeting. Do these sensitive CRCs also express higher ISGs compared to the insensitive cells?**
   - We thank the reviewer for this insightful question. In the revised manuscript, we expanded our analyses by sequencing additional CRC models and found that repeat-element (RE) expression varies across METTL3-sensitive and -insensitive cell lines, whereas sensitivity is more consistently associated with high m^6A levels and elevated basal dsRNA abundance (Figure 6D,E).

     To directly evaluate whether ISG levels predict METTL3 dependency, we compared basal ISG scores with METTL3 dependency probability across CRC cell lines and found no significant correlation (Supplementary Fig. 1K). When extending this analysis across cancer types, we observed context-dependent relationships: ISG scores showed a positive correlation with METTL3 dependency in OCSC and SCLC, but a negative correlation in LUAD (Supplementary Fig. 1M), indicating that ISG expression is not a universal predictor.

     Consistent with this, our RNA-seq data show that HT29, a METTL3-sensitive CRC model, has lower basal ISG expression than METTL3-insensitive lines such as HCT116 and CSC73 (Supplementary Fig. 1L). Importantly, this pattern is biologically plausible: despite high basal dsRNA detected by J2 staining in HT29 cells, these cells exhibit defective MAVS-mediated antiviral signaling due to APC truncations that impair MAVS localization on mitochondria. This mechanism, demonstrated by Li et al. [20] explains why HT29 maintains high dsRNA levels without corresponding ISG activation.

     Taken together, these findings demonstrate that ISG expression is not a reliable biomarker of METTL3 sensitivity in CRC. Instead, sensitivity is more closely linked to basal dsRNA accumulation and m^6A-dependent regulation, rather than to pre-existing interferon pathway activation. (All corresponding figures are provided below).

Figure 14

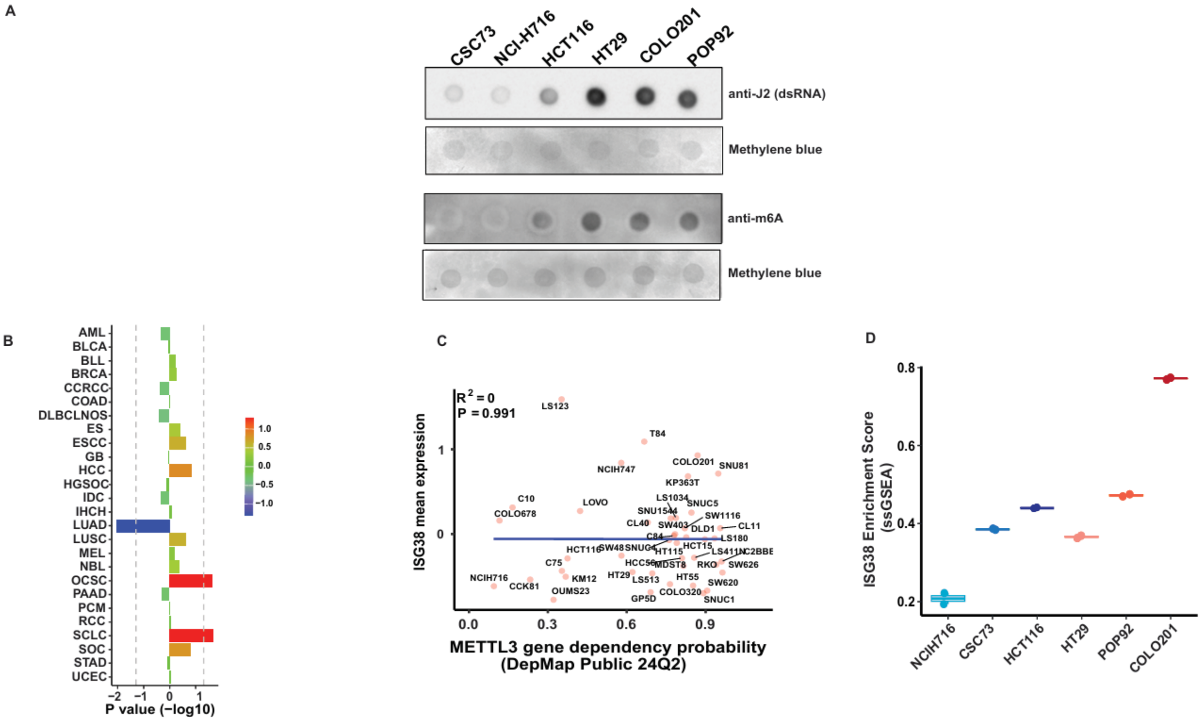

Figure 14. CRC METTL3 dependency does not correlate with basal ISG expression.

(A) Dot blot analysis of double-stranded RNA (dsRNA; detected with the J2 antibody) and m^6A levels in total RNA isolated from the indicated CRC cell lines and PDX models.

(B) Bar plot showing the significance of the linear correlation between METTL3 dependency probability (DepMap 24Q2) and ISG38 mean expression across 20 cancer types. Bar height and colour reflect the p-value scale; negative values indicate a negative association. Horizontal dashed lines denote the cutoffs at log10(0.05) and –log10(0.05).

(C) Scatter plot showing the correlation between ISG38 mean expression and METTL3 dependency probability in CRC cell lines. The blue line represents the linear regression between the two variables.

(D) ISG38 single-sample GSEA (ssGSEA) scores in METTL3-insensitive (NCI-H716, CSC73, HCT116) and METTL3-sensitive (HT29, POP92, COLO201) CRC cells. Data represent two independent experiments.

4. **Figure 4F show higher expressed TEs in HT29 compared to HCT116 (82680 vs 18712). However, the volcano plot appears to show only imperceptible changes; In addition, does different TEs show different expression pattern? Supplementary Figures 3C and 3D should show expression changes for different TE loci, especially Alu elements.**

    - **We thank the reviewer for these helpful suggestions. In the revised manuscript, we have regenerated the volcano plot in Figure 4F (now Figure 3D) to more clearly illustrate the substantially higher expression of TEs in HT29 compared to HCT116. These upregulated TEs in HT29 are significantly enriched for the SINE family (33.93%, OR = 1.2, p < 0.0001) (Supplementary Fig. 4A) and are predominantly**

located within intronic regions (73.31%, OR = 3.9, $p < 0.0001$) (Supplementary Fig. 4C).

We have also regenerated the volcano plots for Supplementary Figures 3C and 3D (now Supplementary Figures 4G and 4I) to separately display the effects of METTL3 knockdown on Alu, LINE, and LTR elements. In HT29 cells, METTL3KD leads to differential expression of hundreds of TEs across all three families, with no single class disproportionately affected. In contrast, TE expression is minimally altered by METTL3^{KD} in HCT116 cells, consistent with their METTL3-insensitive phenotype.

Although METTL3^{KD} in NCI-H716 cells results in significant changes in TE expression, these cells exhibit very low basal m^6A levels and minimal J2-detected dsRNA, and they remain fully insensitive to METTL3 loss. This suggests that the deregulated TEs in NCI-H716 are likely downstream consequences of METTL3 depletion rather than functionally contributing to viral mimicry. Consistent with this, NCI-H716 cells do not activate interferon signaling upon METTL3^{KD}.

Together, these analyses reinforce that TE dysregulation after METTL3^{KD} is context-dependent, and that TE abundance alone does not predict viral mimicry induction. Instead, the combination of high basal m^6A, high dsRNA, and TE accessibility better explains sensitivity to METTL3 targeting in CRC models.

Figure 15

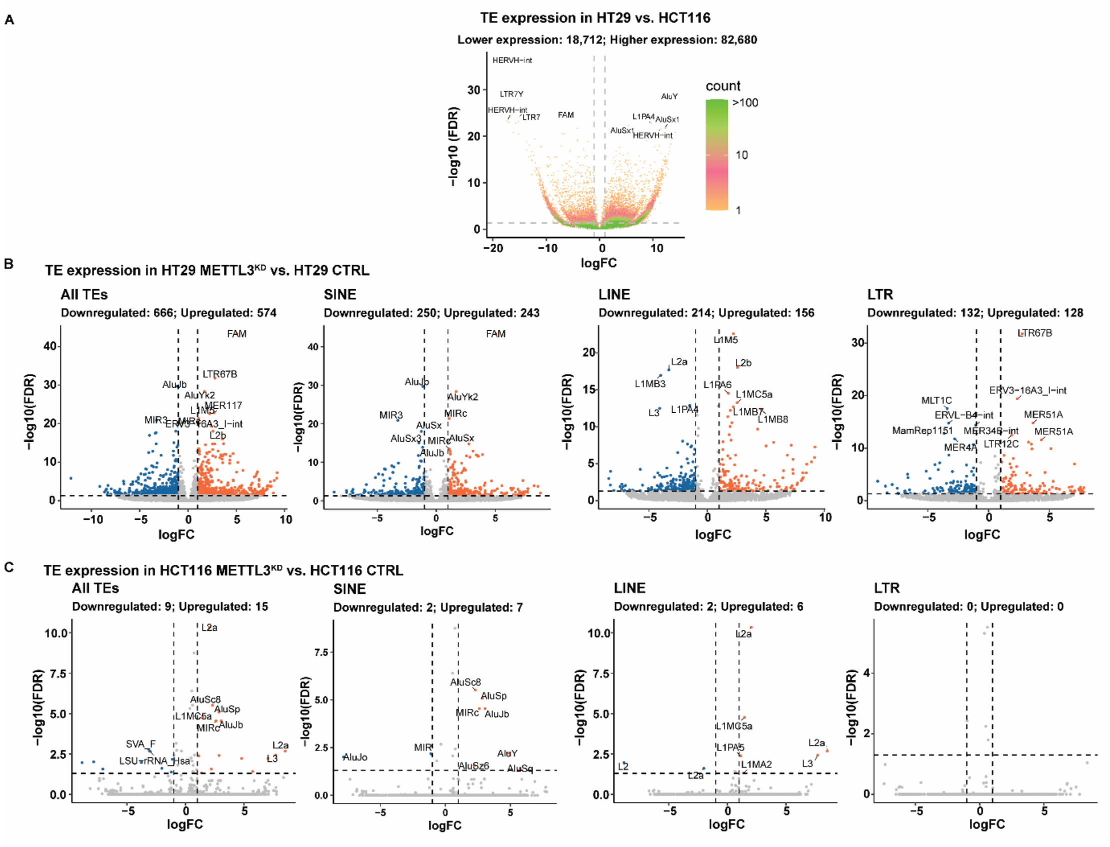

Figure 15. Differential Transposable Element Expression in HT29 vs. HCT116 and in METTL3-Deficient HT29 Cells.

(A) Volcano plot showing differential expression of transposable elements (TEs) between HT29 and HCT116 cells based on RNA-sequencing data. The color scale represents TE counts, and dotted lines indicate the cutoffs used to define significantly differentially expressed TEs (FDR < 0.05 and |fold change| ≥ 1). Data represent two independent experiments.

(B,C) Volcano plots showing changes in TE expression following METTL3 knockdown compared to control in HT29 **(B)** and HCT116 (C) cells. The first column displays overall TE expression changes, while subsequent columns show expression changes for SINE, LINE, and LTR elements separately.

a) **Why does targeting METTL3 specifically induce IR-Alus? Does the METTL14/3 specifically bind Alu RNA?**

- **We thank the reviewer for this important question. In our study, we found that METTL3 depletion reduces m^{6}A levels, which in turn promotes dsRNA formation and activates innate immune signaling. Our MDA5-protection assay indicates that the majority of immunogenic dsRNAs in CRC cells originate from inverted-repeat Alu (IR-Alu) elements.**

  Alu elements are primate-specific SINEs and the most abundant transposable elements in the human genome (>1 million copies) [21]. Because Alus are evolutionarily young, short (~300 bp), and highly sequence-conserved, closely spaced Alu pairs frequently occur in inverted orientations, making them particularly prone to forming stable dsRNA structures. This genomic architecture likely explains why IR-Alus emerge as the predominant source of MDA5-stimulatory dsRNA in human cancer cells.

  Importantly, we do not propose that m^{6}A selectively suppresses dsRNA formation from IR-Alus over other repeat families, nor do we have evidence that METTL3/METTL14 preferentially binds Alu transcripts. Rather, our data support a model in which m^{6}A broadly reduces dsRNA formation across multiple sequence contexts. IR-Alus appear most prominent in our analyses because they are especially abundant and structurally predisposed to forming dsRNA, and because we focused specifically on the dsRNA species that functionally activate viral mimicry and innate immune responses upon METTL3 loss.

  Finally, we note that METTL3^{KD} also alters the expression and/or methylation of other repeat families, including LINE elements. Determining the extent to which these contribute to dsRNA biology or innate immune activation will require future investigation.

b) **Are all IR-Alu molecules mentioned in this manuscript derived from embedded Alu elements transcribed by pol II within host gene transcripts? Does METTL3 KO regulate RNA stability of host genes containing these Alu elements?**

- **We thank the reviewer for this important question.**
  Origin of IR-Alus.

  To determine whether the IR-Alus identified in our study originate from embedded Alu elements within host gene transcripts, we examined their transcriptional orientation relative to overlapping genes in HT29 cells. The majority of both basal and METTL3^{KD}-induced IR-Alus were transcribed in the same (sense) direction as their host genes (Figure 16 shown below). Because intronic and exonic Alu elements embedded within protein-coding genes are co-transcribed by RNA polymerase II as part of the host pre-mRNA, this strand concordance strongly supports that the IR-Alus detected in our assays are predominantly derived from Pol II–transcribed host gene transcripts, rather than from independent Pol III transcription. This conclusion is consistent with recent studies showing that TEs embedded within protein-coding genes represent a major source of endogenous immunogenic dsRNA [22, 23].

- **Effect of METTL3 targeting on host gene RNA stability.**

  To assess whether METTL3 loss alters the stability of host transcripts containing IR-Alus, we performed Actinomycin D chase experiments on both ISG transcripts and several repeat-derived RNAs enriched in the RNase/MDA5 protection assay. These analyses revealed no major differences in RNA half-life between control and METTL3^{KD} cells. Although we were unable to measure stability of every host gene containing IR-Alus individually, the absence of global stability changes in both host-associated repeat transcripts and ISGs suggests that METTL3 loss does not broadly regulate the stability of host genes harboring IR-Alus. Instead, the increased dsRNA signal following METTL3^{KD} is more consistent with changes in structure or accessibility of existing transcripts, rather than changes in their degradation rates.

  Together, these findings indicate that (i) the IR-Alus we detect are primarily derived from Pol II–transcribed host gene transcripts, and (ii) METTL3 loss does not significantly alter the stability of these host transcripts, supporting a model where m^{6}A regulates dsRNA formation rather than host-mRNA decay.

Figure 16

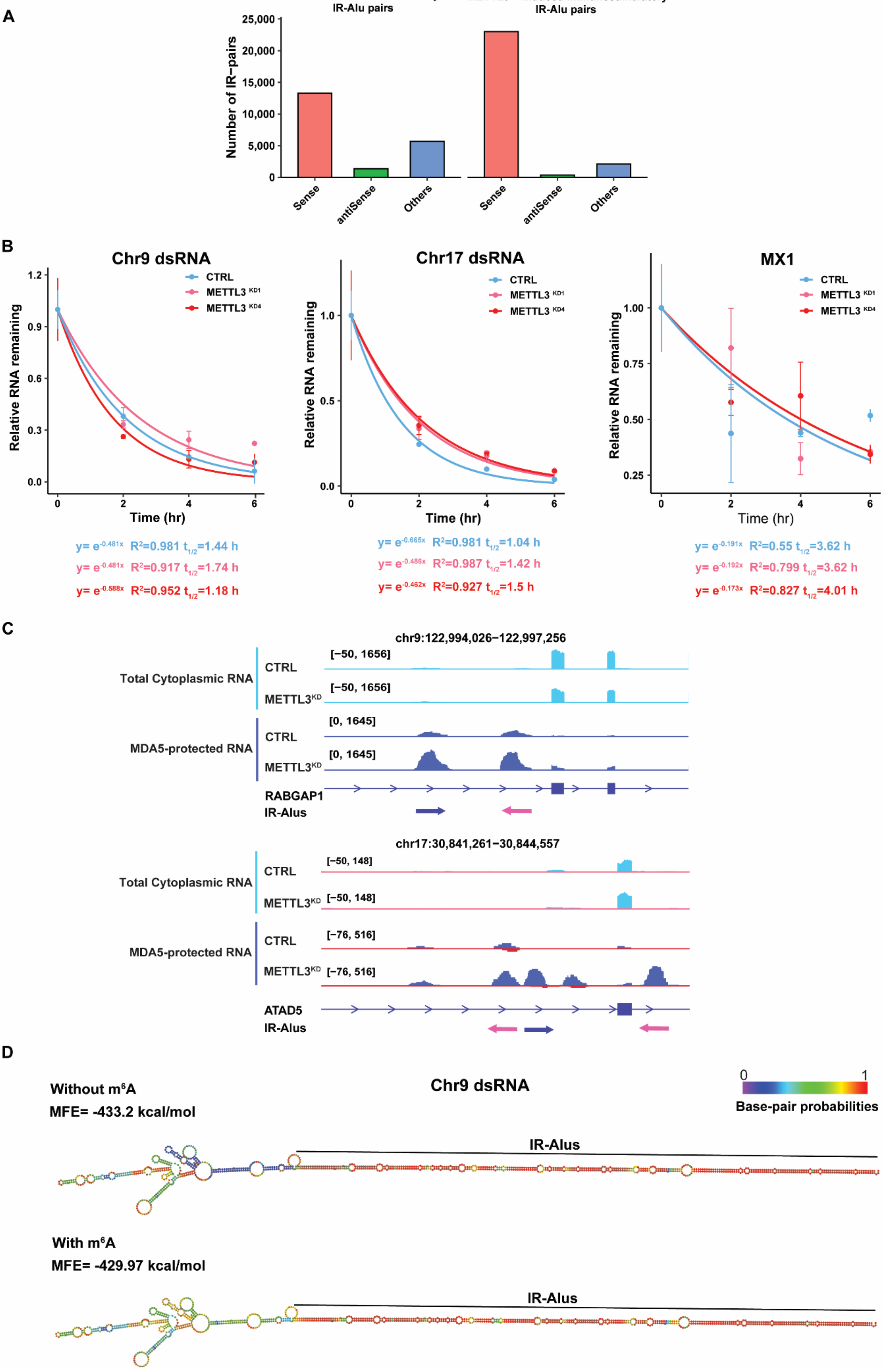
A
Baseline-immunostimulatory IR-Alu pairs
METTL3KD-induced immunostimulatory IR-Alu pairs
Number of IR-pairs
25,000
20,000
15,000
10,000
5,000
0
Sense
antiSense
Others
B
Chr9 dsRNA
Chr17 dsRNA
MX1
CTRL
METTL3 KD1
METTL3 KD4
Relative RNA remaining
Time (hr)
y= e-0.481x R2=0.981 t1/2=1.44 h
y= e-0.481x R2=0.917 t1/2=1.74 h
y= e-0.588x R2=0.952 t1/2=1.18 h
y= e-0.665x R2=0.981 t1/2=1.04 h
y= e-0.486x R2=0.987 t1/2=1.42 h
y= e-0.462x R2=0.927 t1/2=1.5 h
y= e-0.191x R2=0.55 t1/2=3.62 h
y= e-0.192x R2=0.799 t1/2=3.62 h
y= e-0.173x R2=0.827 t1/2=4.01 h
C
chr9:122,994,026−122,997,256
Total Cytoplasmic RNA
MDA5-protected RNA
CTRL
METTL3KD
[−50, 1656]
[0, 1645]
RABGAP1
IR-Alus
chr17:30,841,261−30,844,557
[−50, 148]
[−76, 516]
ATAD5
D
Chr9 dsRNA
Without m6A
MFE= -433.2 kcal/mol
With m6A
MFE= -429.97 kcal/mol
Base-pair probabilities
0
1

Figure 16. METTL3 depletion increases hypomethylated IR-Alu–derived dsRNAs without altering RNA stability in HT29.

(A) Counts of baseline (n = 20,304) and $METTL3^{KD}$-induced (n = 25,462) IR-pairs in HT29 cells, categorized by their transcriptional orientation relative to host genes. “Sense” and “antisense” indicate IR pairs transcribed in the same or opposite direction as their hosted genes, respectively, while “others” represent intergenic IR pairs.

(B) HT29 control and $METTL3^{KD}$ cells were treated with actinomycin D (1 μg/mL), and RNA was extracted at the indicated time points for RT–qPCR analysis. The degradation rate of each transcript was fitted using $y = e^{-k(decay)x}$. Fits with $R^2 \geq 0.95$ were considered robust; RNA half-lives were calculated as $t_{1/2} = ln(2) / k(decay)$. $n = 2$ technical replicates.

(C) Representative genomic tracks showing $METTL3^{KD}$-induced immunostimulatory inverted-repeat Alu (IR-Alu) elements highlighted in pink and blue. The top two tracks display total cytoplasmic RNA with sense (sky blue) and antisense (pink) reads distinguished. The next two tracks show RNase A–digested cytoplasmic RNA, with sense (dark blue) and antisense (red) reads indicated.

(D) Representative examples of predicted dsRNA structures formed by IR-Alu pairs, shown either in their unmethylated form or with all adenosines in m6A motif (DRACH) substituted to mimic m^6A-modified positions. RNAfold-predicted secondary structures and corresponding minimum free energy (MFE)are shown for an IR-Alu locus on chromosome 9 and chromosome 17.

References:

1. Scholch, S., et al., *Circulating tumor cells exhibit stem cell characteristics in an orthotopic mouse model of colorectal cancer.* Oncotarget, 2016. **7**(19): p. 27232-42.
2. Roost, C., et al., *Structure and thermodynamics of N6-methyladenosine in RNA: a spring-loaded base modification.* J Am Chem Soc, 2015. **137**(5): p. 2107-15.
3. Liu, N., et al., *N(6)-methyladenosine-dependent RNA structural switches regulate RNA-protein interactions.* Nature, 2015. **518**(7540): p. 560-4.
4. Spitale, R.C., et al., *Structural imprints in vivo decode RNA regulatory mechanisms.* Nature, 2015. **519**(7544): p. 486-90.
5. Pan, J., et al., *METTL3 promotes colorectal carcinoma progression by regulating the m6A-CRB3-Hippo axis.* J Exp Clin Cancer Res, 2022. **41**(1): p. 19.
6. Shen, C., et al., *m(6)A-dependent glycolysis enhances colorectal cancer progression.* Mol Cancer, 2020. **19**(1): p. 72.
7. Sun, Y., W. Gong, and S. Zhang, *METTL3 promotes colorectal cancer progression through activating JAK1/STAT3 signaling pathway.* Cell Death Dis, 2023. **14**(11): p. 765.
8. Wang, Y., et al., *hsa_circ_0000523/miR-let-7b/METTL3 axis regulates proliferation, apoptosis and metastasis in the HCT116 human colorectal cancer cell line.* Oncol Lett, 2022. **23**(6): p. 186.
9. Ahmad, S., et al., *Breaching Self-Tolerance to Alu Duplex RNA Underlies MDA5-Mediated Inflammation.* Cell, 2018. **172**(4): p. 797-810 e13.
10. Mehdipour, P., et al., *Epigenetic therapy induces transcription of inverted SINEs and ADAR1 dependency.* Nature, 2020. **588**(7836): p. 169-173.
11. Center, B.I.T.G.D.A. *Mutation Analysis (MutSigCV v0.9).* 2016; Available from: https://doi.org/10.7908/C1G44PPJ.
12. Kierzek, E. and R. Kierzek, *The thermodynamic stability of RNA duplexes and hairpins containing N6-alkyladenosines and 2-methylthio-N6-alkyladenosines.* Nucleic Acids Res, 2003. **31**(15): p. 4472-80.
13. Shi, H., et al., *NMR Chemical Exchange Measurements Reveal That N(6)-Methyladenosine Slows RNA Annealing.* J Am Chem Soc, 2019. **141**(51): p. 19988-19993.
14. Gao, Y., et al., *m(6)A Modification Prevents Formation of Endogenous Double-Stranded RNAs and Deleterious Innate Immune Responses during Hematopoietic Development.* Immunity, 2020. **52**(6): p. 1007-1021 e8.
15. Chiappinelli, K.B., et al., *Inhibiting DNA Methylation Causes an Interferon Response in Cancer via dsRNA Including Endogenous Retroviruses.* Cell, 2015. **162**(5): p. 974-86.
16. Roulois, D., et al., *DNA-Demethylating Agents Target Colorectal Cancer Cells by Inducing Viral Mimicry by Endogenous Transcripts.* Cell, 2015. **162**(5): p. 961-73.
17. de Castro, F.A., et al., *Ratio of stemness to interferon signalling as a biomarker and therapeutic target of myeloproliferative neoplasm progression to acute myeloid leukaemia.* Br J Haematol, 2024. **204**(1): p. 206-220.
18. Organization, W.H. *Colorectal cancer.* 2023; Available from: https://www.who.int/news-room/fact-sheets/detail/colorectal-cancer.
19. Sung, H., et al., *Colorectal cancer incidence trends in younger versus older adults: an analysis of population-based cancer registry data.* Lancet Oncol, 2025. **26**(1): p. 51-63.
20. Li, S.Y., et al., *Truncated APC impairs innate immune response by targeting MAVS on mitochondria in colorectal cancer.* J Transl Med, 2025. **23**(1): p. 1252.
21. Deininger, P., *Alu elements: know the SINEs.* Genome Biol, 2011. **12**(12): p. 236.
22. Feng, S., et al., *Inhibiting EZH2 targets atypical teratoid rhabdoid tumor by triggering viral mimicry via both RNA and DNA sensing pathways.* Nat Commun, 2024. **15**(1): p. 9321.

23. Gázquez-Gutiérrez, A., et al., *Control of retrotransposon-driven activation of the interferon response by the double-stranded RNA binding protein DGCR8*. bioRxiv, 2025: p. 2025.05.28.656609.

REVIEWERS' COMMENTS

Reviewer #1 (Remarks to the Author):

The revised MS by Wang et al. addresses most of the questions and comments by Reviewer 1; esp. concerning the specific remarks 1-10.

Concerning the specific remark #7 regarding, dsRNA accumulation rather than stability" the authors did many additional experiments (Fig. 2 in reviewer comments). However, the use of RNAfold (which did not reveal substantial differences between methylated and unmethylated RNA) is not sufficient and actually inadequate for this question. Wang (ref #43) which is only used in ln273 as a common reference showed that the binding of YTHDF2, as well as IGF2BPs (see review by Wei G 2024, Biochem Soc Trans) are responsible for the stability of m6A RNA. The authors should consider these RNA-protein interactions, which also apply to dsRNA as shown by Yang et al. (2025, Nature Commun) rather than RNAfold studies.

In summary, the present study by Wang et al. demonstrates a thorough investigation about m6A RNA, dsRNA and METTL3 in colorectal cancer cell lines. It is important to confirm known cellular processes between different cancer types using cell lines. However, the reply by the authors to all other publications showing a similar importance of m6A, dsRNA, METTL3 with innate immunity (main questions 1-13 for reviewer 1) was always „colorectal cell line and Alu dsRNA". This is in my opinion not sufficient for Nature Communications, even after a novel paper in PNAS (Li Y et al. 2025), which showed that next to LINE and ERV, also Alus are enriched in METTL3 knock-out cells (although in breast cell lines).

- **We agree with the reviewer that m^6A reader proteins such as YTHDF2 and IGF2BP family members are established regulators of RNA stability, as demonstrated in prior studies, including Wang et al. (Nature, 2014), Wei et al. (Biochem. Soc. Trans., 2024), and more recently Yang et al. (Nat. Commun., 2025), which also highlight the role of RNA–protein interactions in dsRNA regulation. We do not question the relevance of these pathways. However, our study does not aim to dissect the individual kinetic contributions of RNA decay versus production, nor to assign a dominant role to a specific reader-mediated mechanism.**

- **Instead, our experimental framework resolves the biologically relevant steady-state outcome: whether endogenous repeat-derived dsRNAs accumulate to a level and structural state sufficient to engage innate immune sensors and downstream signaling pathways. As clarified in the revised Discussion, RNA accumulation and RNA stability are not viewed as mutually exclusive mechanisms in our model. Rather, m^6A-dependent effects on RNA availability, including transcriptional output, persistence, and decay, are expected to cooperate in determining steady-state dsRNA abundance. We have now explicitly stated this limitation and interpretation in the manuscript and cited established reader-mediated pathways that may plausibly contribute to the observed dsRNA changes.**

- With respect to RNAfold analyses, these were included only as exploratory in silico assessments and were not used to draw mechanistic conclusions about dsRNA stability or reader interactions. Importantly, our conclusions do not rely on RNAfold predictions. Instead, they are supported by orthogonal experimental evidence, including RNase/MDA5 protection assays, MAVS signaling, PKR-mediated cytotoxicity, and functional transfer of immunostimulatory RNA, all of which directly link steady-state dsRNA abundance to innate immune activation.

- Regarding the broader concern that our conclusions rely on "colorectal cell lines and Alu dsRNA," we would emphasize that our study does not claim that Alu elements or CRC represent unique exceptions within m^6A biology. Rather, the novelty lies in demonstrating substantial heterogeneity within a single cancer type, showing that comparable repeat expression does not uniformly translate into dsRNA formation or immune activation. Our data identify IR-Alu elements as the dominant functional source of MDA5-engaging dsRNA specifically in human CRC, based on direct functional assays, not transcript enrichment alone. This mechanistic hierarchy and threshold-dependent activation are not addressed in prior studies.

- We acknowledge the recent work by Li et al. (PNAS, 2025), which reports enrichment of Alu, LINE, and ERV transcripts and interferon induction in METTL3-deficient mammary epithelial cells. As stated in the revised Discussion, this study supports a conserved role for METTL3 in restraining repeat-derived immunostimulatory RNA across epithelial contexts. However, it does not examine differential sensitivity within a cancer type, identify the dominant dsRNA species responsible for immune activation, or address the threshold-dependent nature of innate immune engagement, all of which are central findings of our work.

- In summary, while RNA–protein interactions and reader-mediated decay pathways are clearly relevant to m^6A biology, our study is focused on the experimentally supported endpoint that determines phenotype: steady-state endogenous dsRNA abundance and its capacity to activate innate immune signaling. We believe the revised manuscript now clearly communicates this scope and appropriately situates our findings within the broader literature.

Reviewer #2 (Remarks to the Author):

The authors have addressed all of my concerns and the manuscript is significantly strengthened. I recommend it for publication at Nature Communications.

- We thank the reviewer for their positive evaluation and their constructive comments, which have helped to strengthen the manuscript.

Reviewer #3 (Remarks to the Author):

Thank you for the revised manuscript. The authors have addressed all my concerns.

- **We thank the reviewer for their thoughtful assessment of our revised manuscript and appreciate their acknowledgment that the concerns have been satisfactorily addressed.**

Reviewer #4 (Remarks to the Author):

The authors have properly addressed all my concerns and adequately revised the manuscript. I recommend it for publication.

- **We appreciate the reviewer's positive assessment and recommendation for publication.**
